# Effects of SGLT2 inhibitors on UTIs and genital infections in type 2 diabetes mellitus: a systematic review and meta-analysis

**DOI:** 10.1038/s41598-017-02733-w

**Published:** 2017-06-06

**Authors:** Jiali Liu, Ling Li, Sheyu Li, Pengli Jia, Ke Deng, Wenwen Chen, Xin Sun

**Affiliations:** 1 Chinese Evidence-based Medicine Center, West China Hospital, Sichuan University, Chengdu, Sichuan China; 2 Department of Endocrinology and Metabolism, West China Hospital, Sichuan University, Chengdu, Sichuan China

## Abstract

Previous trial evidence suggested potential risk of serious urinary tract infections (UTIs) and genital infections in type 2 diabetes patients using sodium glucose co-transporter-2 inhibitors (SGLT2) inhibitors. We conducted a systematic review and meta-analysis to assess the effects of SGLT2 inhibitors on UTIs and genital infections in patients with type 2 diabetes. In total, 77 RCTs involving 50,820 participants were eligible. The meta-analyses of randomized controlled trials (RCTs) showed no significant difference in UTIs between SGLT2 inhibitors versus control (2,526/29,086 vs. 1,278/14,940; risk ratio (RR) 1.05, 95% confidence interval (CI) 0.98 to 1.12; moderate quality evidence), but suggested increased risk of genital infections with SGLT2 inhibitors (1,521/24,017 vs. 216/12,552; RR 3.30, 95% CI 2.74 to 3.99; moderate quality evidence). Subgroup analyses by length of follow up (interaction p = 0.005), type of control (interaction p = 0.04) and individual SGLT2 inhibitors (interaction p = 0.03) also showed statistically significant differences in genital infections. The upcoming major trials may provide important additional insights on UTIs, and more efforts are needed to address comparative effects of each individual SGLT2 inhibitors on the infections.

## Introduction

Sodium glucose co-transporter-2 (SGLT2) inhibitors are one of the newest classes of glucose-lowering drugs for patients with type 2 diabetes mellitus (T2DM)^1^. It exerts the glucose lowering effect by down-regulating the renal threshold for glucose excretion and increasing urinary glucose excretion^2^. Meta-analyses of randomized controlled trials (RCTs) have proven that this drug class can reduce glycated haemoglobin (HbA1c) levels, fasting plasma glucose, body weight and blood pressure, without increasing the risk of any hypoglycemic events^3, 4^. The US Food and Drug Administration (FDA) and the European Medicines Agency (EMA) approved dapagliflozin, canagliflozin and empagliflozin for clinical use in patients with T2DM^5–10^, and the other three, including ipragliflozin, luseogliflozin, and tofogliflozin, approved in Japan^11–13^.

T2DM increases the risk of urinary tract infections (UTIs) and non-sexually transmitted genital infections due to the elevated urinary glucose^14^. The pharmacologically-induced urinary glucose with SGLT2 inhibitors may cause additional growth of commensal genital microorganisms. As such, the risk of genital infections and UTIs is likely to be further increased in patients administered with SGLT2^15^. In December 2015, the FDA warned that SGLT2 inhibitors may result serious urinary tract infections^16^. A few systematic reviews and meta-analyses examined this issue; however, the findings were inconsistent^3, 4, 17, 18^. A definitive conclusion is yet to be established.

We therefore conducted a systematic review and meta-analysis, collating the most recent evidence into the prior information, aiming to establish the impact of SGLT2 inhibitors on UTIs and genital infections in patients with type 2 diabetes.

## Methods

We reported the review following the Preferred Reporting Items for Systematic Reviews and Meta-Analyses (PRISMA) statement^19^.

### Data Sources and Searches

We searched the following electronic databases from inception to 26 February 2016 for potentially relevant articles: PubMed, EMBASE, and the Cochrane Central Register of Controlled Trials (CENTRAL), using both Medical Subject Heading (MeSH) and free text terms (Supplementary material: Search strategies). We also searched ClinicalTrials. gov to identify additional relevant trials, including serious adverse events and adverse events with frequency over 5%^20, 21^.

### Eligibility criteria

We included randomized controlled trials (RCTs) of adult type 2 patients that compared SGLT2 inhibitor against placebo, lifestyle modification, or active antidiabetic drugs; had the study duration of at least 12 weeks; and explicitly reported outcome data regarding UTIs, genital infections, events suggestive of UTIs, or events suggestive of genital infections. The last two outcomes were defined as both non-specific signs, symptoms and abnormal laboratory findings suggestive of UTIs or genital infections as well as confirmed infections.

We planned to include non-randomized studies (non-randomized controlled trials, cohort studies and case-control studies), using eligibility criteria for patients, interventions and control similar with the above. No studies, however, proved eligible.

### Study selection

Paired reviewers, independently and in duplicate, screened the searched citations for initial eligibility and full texts for final eligibility, assessed risk of bias, and collected data from included studies, using standardized forms with detailed instructions. Disagreements were resolved through discussion between the two reviewers or arbitrated by a third reviewer.

### Risk of bias assessment

We used the Cochrane Collaboration’s tool^22^ to assess the risk of bias of each RCT. When assessing the risk of bias for such items as random sequence generation, allocation concealment and blinding, we used the option of “probably yes” or “probably no” to replace “unclear”^23^. This approach has proved reliable and valid, and has been used before^24^.

### Data extraction

For each eligible RCT, we collected the following information.

- Study characteristics, including author name, year of publication, study design, sample size, length of follow-up, study phase, number of study sites, source of funding, and countries involved;
- Patient characteristics, including age, gender, duration of diabetes, body mass index (BMI), HbA1c level, and fasting plasma glucose (FPG);
- Intervention and control characteristics, including baseline treatment, and details of SGLT2 inhibitors and control group;
- Outcomes, including the events of urinary tract infections (UTIs), suggestive of UTIs, genital infections and suggestive of genital infections as well as the patients included for analyses in each group.

For a trial, if the outcome data were not explicitly reported in the published study report, but available from the corresponding trial registry (ClinicalTrials.gov), we collected the outcome data from the trial registry. For an extension trial, if the initial treatment intervention was switched, we used the outcome data prior to that point. When a trial published multiple reports or follow up points, we collected all reports into a single study and used the data with longest follow up^25^.

### Data analysis and rating quality of evidence

We separately analyzed UTIs, events suggestive of UTIs, genital infections, and events suggestive of genital infections. We used random-effects Mantel-Haenszel method to calculate relative risk (RR) and 95% confidence intervals (95% CIs) and explored statistical heterogeneity with the Cochran chi-square test (p value) and I-squared statistic (I^2^). We also calculated the number needed for harm (NNH) for each outcome if the finding was significant.

According to the guidance for subgroup analyses^26^, we explored the following subgroup hypotheses to examine variability between studies: type of control (SGLT2 inhibitors vs. placebo, SGLT2 inhibitors vs. active treatment), length of follow up (26 weeks or shorter, 26–52 weeks, over 52 weeks), individual SGLT2 inhibitors (different SGLT2 inhibitors vs. control), and gender (male vs. female). For the analysis, we used the test for interaction by comparing the pooled estimates of across the defined subgroups. We also conducted random-effects multiple meta-regression analyses adjusting for type of control, length of follow up and individual SGLT2 inhibitors. The gender was not included for multiple regression analyses because only a limited number of trials reported this information.

We also conducted sensitivity analyses to examine the stability of our findings using alternative effect measures (odds ratio (OR) vs. RR), analysis models (fixed effect vs. random effects). We explored publication bias by applying funnel plot and Egger’s test. Finally, we used the Grading of Recommendations Assessment, Development and Evaluation (GRADE) system to assess the quality of evidence by outcomes^27^.

## Results

**Figure 1:**
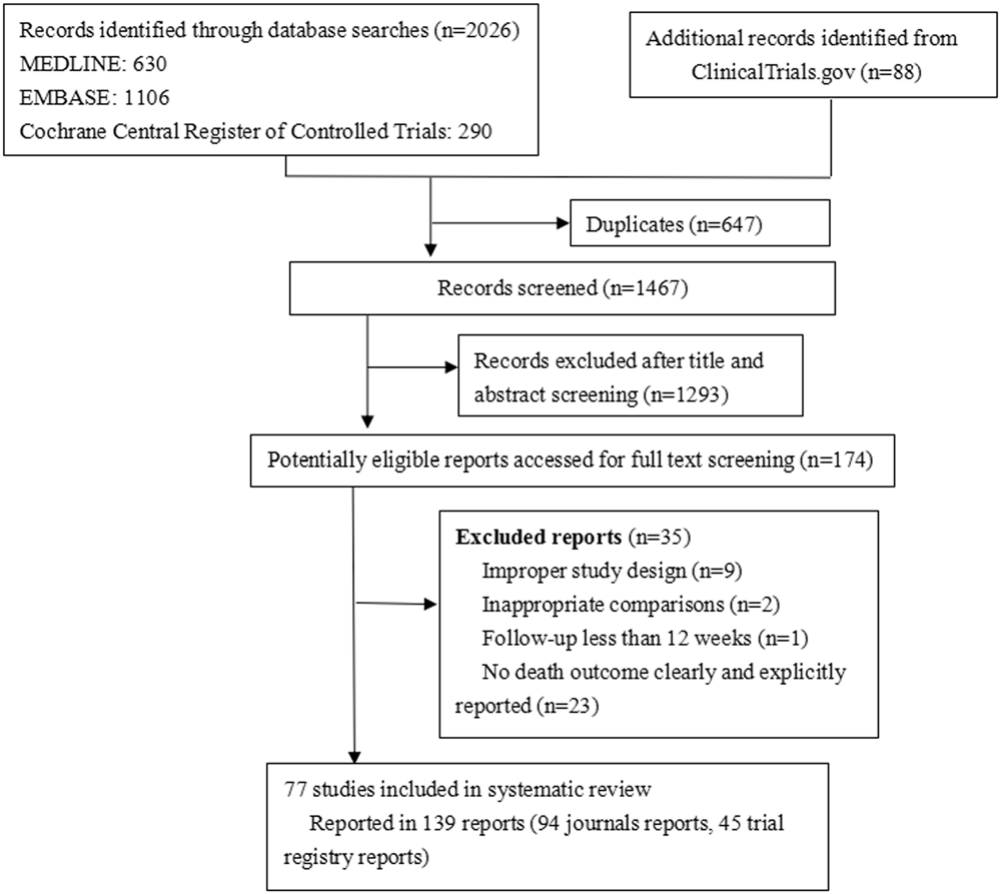
Flow chart of study selection

The process of study selection was shown in Fig.1. We initially identified 2,114 citations. After title and abstract screening, 174 were potentially eligible. Finally, 77 RCTs, published in 143 reports, met the inclusion criteria, and one trial^28^ recruited patients with established cardiovascular disease. No non-randomized studies were eligible.

### Study Characteristics

All 77 RCTs were sponsored by pharmaceutical companies. Among these trials, 58 (75.3%) were international studies, 51 (66.2%) were specifically labeled as phase III trials. Of these 77 RCTs, 77 (100%) adequately generated their randomization sequence; 75 (97.4%) adequately concealed allocation; 74 (96.1%) blinded patients and caregivers; the overall risk of bias was low. The details regarding risk of bias of each study was presented in Supplementary Table 1S.

The patient baseline characteristics of 77 trials were shown in Supplementary Table 2S. In total, those 77 trials enrolled 50,820 participants with sample size ranging from 44 to 7,028. The average duration of diabetes was 0.4 to 16.9 years across trials; the length of follow up ranged from 12 to 161 weeks (median 24 weeks); the mean age of patients ranged from 51.3 to 68.5 years old, mean BMI 24.8 to 35.5 kg/m², mean baseline HbA1c level 7.2% to 9.1%, and mean FPG 7.7 to 10.3 mmol/L.

Among those 77 trials, 28 examined dapagliflozin, 18 empagliflozin, 15 canagliflozin, 8 ipragliflozin, and the other 8 trials tested luseogliflozin, remogliflozin, tofogliflozin, ertugliflozin and sotagliflozin. 35 used SGLT2 inhibitors as monotherapy, 46 as add-on/combination therapy, and four used as both treatment options (Supplementary Table 3S).

Of those 77 trials, 68 reported UTIs, 17 reported events suggestive of UTIs, 56 reported genital infections, and 15 reported events suggestive of genital infections (Table1 and Supplementary Table 3S).

### Effects on urinary tract infections

**Figure 2:**
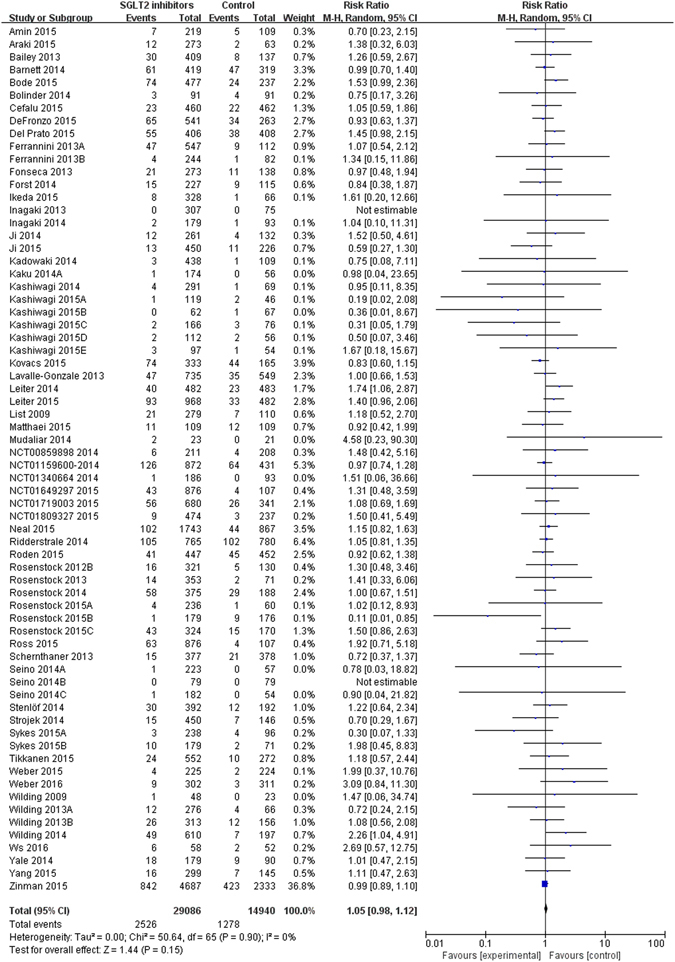
UTIs in type 2 diabetes patients receiving SGLT2 inhibitors versus control in randomized controlled trials.

Those 68 trials reporting UTIs documented 3,804 UTIs events from 44,026 patients who used at least one medication (raw event rate 8.6%). Two trials^29, 30^ reported zero events among participants. The meta-analysis of 68 trials showed no significant difference in urinary tract infections between SGLT2 inhibitors versus control (SGLT2 inhibitors: 2,526/29,086, control: 1,278/14,940; risk ratio (RR) 1.05, 95% confidence interval (CI) 0.98 to 1.12, I^2^ = 0; RD 47 more, 95% CI 19 more to 112 more per 1000 over 5 years) (Table 1 and Fig. 2). There was no evidence of publication bias for UTIs (Supplementary Figure 1S, Egger’s test p = 0.45), and sensitivity analyses did not show important changes in pooled effects. The quality of evidence was moderate due to imprecision (Table 2).

**Table 1:** Risk of UTIs and genital infections among patients with type 2 diabetes receiving SGLT2 inhibitors.

Comparison	Number of studies (Events, patients)	SGLT2 inhibitors (events/patients)	Control (events/patients)	Relative risk (95%CI), interaction test P	P value of multiple meta-regression
**Urinary tract infections**					
**SGLT2 inhibitors vs. control (overall)**	68 (3804, 44026)	2526/29086	1278/14940	1.05 (0.98 to 1.12)	
Subgroup by type of control				Interaction p = 0.36	P = 0.66
SGLT2 inhibitors vs. placebo	58 (3079, 35596)	2101/24338	978/11258	1.03 (0.96 to 1.10)	
SGLT2 inhibitors vs. active drugs	22 (1047, 13370)	671/8463	376/4907	1.10 (0.96 to 1.26)	
Subgroup by length of follow up				Interaction p = 0.78	P = 0.44
26 weeks or shorter	39 (579, 16289)	437/11770	142/4519	1.08 (0.89 to 1.31)	
26-52 weeks	16 (859, 11620)	539/7194	320/4426	1.03 (0.91 to 1.18)	
Over 52 weeks	13 (2398, 16931)	1582/10936	816/5995	1.10 (0.98 to 1.25)	
Subgroup by individual SGLT2 inhibitors				Interaction p = 0.03	P = 0.01
Canagliflozin	15 (687, 11723)	468/7803	219/3920	1.13 (0.97 to 1.33)	
Dapagliflozin	18 (463, 8337)	304/4902	159/3435	1.34 (1.11 to 1.63)	
Empagliflozin	18 (2568, 20306)	1706/13941	862/6365	1.00 (0.93 to 1.08)	
Ipragliflozin	8 (70,1968)	45/1396	25/572	0.75 (0.46 to 1.22)	
Other agents	8 (43, 2210)	31/1622	12/588	0.82 (0.42 to 1.60)	
Subgroup by gender				Interaction p = 0.42	Not applicable
Male	18 (745, 11290)	510/7562	235/3728	1.10 (0.95 to 1.28)	
Female	19 (1811, 7298)	1179/4805	632/2493	1.02 (0.90 to 1.15)	
**Events suggestive of urinary tract infections**					
**SGLT2 inhibitors vs. control (overall)**	17 (499, 7145)	353/4613	146/2532	1.29 (1.06 to 1.57)	
Subgroup by type of control				Interaction p = 0.44	P = 0.50
SGLT2 inhibitors vs. placebo	17 (492, 70631)	353/4613	139/2450	1.31 (1.08 to 1.59)	
SGLT2 inhibitors vs. active drugs	2 (33, 385)	26/303	7/82	0.92 (0.39 to 2.17)	
Subgroup by length of follow up				Interaction p = 0.89	P = 0.90
26 weeks or shorter	7 (68, 1730)	51/1238	17/492	1.20 (0.70 to 2.08)	
26-52 weeks	5 (227, 3354)	142/1898	85/1456	1.22 (0.86 to 1.73)	
Over 52 weeks	5 (204, 2061)	160/1477	44/584	1.37 (0.95 to 1.97)	
Subgroup by gender				Interaction p = 0.29	Not applicable
Male	9 (140, 3051)	99/1828	41/1223	(1.12 to 2.35)	
Female	9 (305, 2324)	209/1487	96/837	1.28 (0.99 to 1.64)	
**Genital infections**					
**SGLT2 inhibitors vs. control (overall)**	56 (1737, 36569)	1521/24017	216/12552	3.30 (2.74 to 3.99)	
Subgroup by type of control				Interaction p = 0.04	P = 0.51
SGLT2 inhibitors vs. placebo	46 (1177, 28153)	1041/19275	136/8878	2.87 (2.27 to 3.62)	
SGLT2 inhibitors vs. active drugs	20 (709, 11978)	620/7649	89/4329	4.06 (3.24 to 5.08)	
Subgroup by length of follow up				Interaction p = 0.005	P = 0.02
26 weeks or shorter	33 (274, 12043)	238/8389	36/3654	2.10 (1.47 to 2.98)	
26-52 weeks	13 (544, 9137)	474/5802	70/3335	3.26 (2.24 to 4.74)	
Over 52 weeks	10 (919, 15396)	809/9826	110/5570	4.23 (3.36 to 5.33)	
Subgroup by individual SGLT2 inhibitors				Interaction p=0.03	P=0.09
Canagliflozin	13 (679, 10258)	607/6668	72/3590	4.45 (3.49 to 5.67)	
Dapagliflozin	11 (163, 4275)	133/2377	30/1898	3.22 (1.95 to 5.32)	
Empagliflozin	16 (823, 17963)	719/12046	104/5917	3.14 (2.29 to 4.30)	
Ipragliflozin	8 (31, 1968)	24/1396	7/572	1.30 (0.57 to 2.97)	
Other agents	8 (41, 2112)	38/1530	3/582	2.13 (0.80 to 5.682)	
Subgroup by gender				Interaction p = 0.74	Not applicable
Male	28 (620, 16478)	550/10844	70/5634	3.62 (2.66 to 4.93)	
Female	29 (848, 11040)	741/7163	107/3877	3.38 (2.61 to 4.37)	
**Events suggestive of genital infections**					
**SGLT2 inhibitors vs. control (overall)**	15 (363, 6712)	328/4311	35/2401	3.92 (2.66 to 5.78)	
Subgroup by type of control				Interaction p = 0.83	P = 0.50
SGLT2 inhibitors vs. placebo	15 (363, 6686)	328/4311	35/2375	3.87 (2.64 to 5.66)	
SGLT2 inhibitors vs. active drugs	1 (2, 50)	2/24	0/26	5.40 (0.27 to 107.09)	
Subgroup by length of follow up				Interaction p = 0.06	P = 0.07
26 weeks or shorter	5 (27, 1297)	23/936	4/361	1.91 (0.73 to 4.98)	
26-52 weeks	5 (154, 3354)	141/1898	13/1456	7.04 (3.47 to 14.27)	
Over 52 weeks	5 (182, 2061)	164/1477	18/584	3.10 (1.91 to 5.01)	
Subgroup by gender				Interaction p = 0.10	Not applicable
Male	9 (103, 3051)	98/1828	5/1223	6.69 (3.14 to 14.29)	
Female	9 (243, 2324)	211/1487	32/837	3.24 (2.10 to 5.01)	

Note: the gender was not included in the multiple meta-regression analyses, because only a limited number of trials reported this information.

Other agents included luseogliflozin, remogliflozin, tofogliflozin, ertugliflozin and sotagliflozin; we combined those trials because the number of trials was too few.

**Table 2:** GRADE evidence profile of SGLT2 inhibitors and urinary tract infections and genital infections in patients with type 2 diabetes.

Quality assessment						Summary of findings					Quality of evidence
No of participants (studies) Follow-up time	Risk of bias	Inconsistency	Indirectness	Imprecision	Publication bias	Study event rates		Relative risk (95% CI)	Anticipated absolute effects (5-year time frame)		
						With control	With SGLT2 inhibitors		Risk with control	Risk difference with SGLT2 inhibitors (95% CI)	
**Urinary tract infections (UTIs)**											
44026 (68) 12-208 weeks	No serious limitations	No serious limitations	No serious limitations	**Serious limitations** ^**1**^	Undetected	1278/14940 (8.6%)	2526/29086 (8.7%)	**RR 1.05** (0.98 to 1.12)	932 per 1000^2^	47 more (19 fewer to 112 more)	⊕⊕⊕Ο **Moderate** due to imprecision
**Events suggestive UTIs**											
7145 (17) 12-104 weeks	No serious limitations	No serious limitations	No serious limitations	No serious limitations	Undetected	146/2532 (5.8%)	353/4613 (7.7%)	**RR 1.29** (1.06 to 1.57)	314 per 1000^3^	91 more (19 more to 179 more)	⊕⊕⊕⊕ **High**
**Genital infections**											
36569 (56) 12-208 weeks	No serious limitations	No serious limitations	No serious limitations	No serious limitations	**Strongly suspected** ^**4**^	216/12552 (1.7%)	1521/24017 (6.3%)	**RR 3.30** (2.74 to 3.99)	184 per 1000^5^	423 more (320 more to 550 more)	⊕⊕⊕Ο **Moderate** due to publication bias
**Events suggestive genital infections**											
6712 (15) 12-104 weeks	No serious limitations	No serious limitations	No serious limitations	No serious limitations	**Strongly suspected** ^**6**^	35/2401 (1.5%)	328/4311 (7.6%)	**RR 3.92** (2.66 to 5.78)	81 per 1000^7^	237 more (134 more to 387 more)	⊕⊕⊕Ο **Moderate** due to publication bias

^1^The meta-analysis failed to meet optimal information size (OIS) criteria.

^2^Baseline risk estimate for UTIs in a 5-year time frame comes from the control arm of included 68 studies with 1278 events in 14940 participants (86 per 1000) over amedian follow up of 24 weeks.

^3^Baseline risk estimate for suggestive UTIs in a 5-year time frame comes from the control arm of included 17 studies with 146 events in 2532 participants (58 per 1000) over a median follow up of 48 weeks.

^4^Funnel plot suggested some asymmetry (Supplementary Figure 3S), and Egger’s test showed publication bias (p = 0.01).

^5^Baseline risk estimate for genital infections in a 5-year time frame comes from the control arm of included 56 studies with 216 events in 12552 participants (17 per 1000) over a median follow up of 24 weeks.

^6^Funnel plot suggested some asymmetry (Supplementary Figure 4S), and Egger’s test showed publication bias (p = 0.001).

^7^Baseline risk estimate for suggestive genital infections in a 5-year time frame comes from the control arm of included 15 studies with 35 events in 2401 participants (15 per 1000) over a median follow up of 48 weeks.

Subgroup analyses by type of individual SGLT2 inhibitors (interaction p = 0.03) showed a statistically significant difference in UTIs (Table 1 and Supplementary Figure 5S), but other analyses by type of control (interaction p = 0.36), length of follow up (interaction p = 0.78), and gender (interaction p = 0.42) showed no statistical differences (Table 1 and Supplementary Figure 6S–8S). The multiple meta-regression analysis showed similar findings (individual SGLT2 inhibitors: p = 0.01; type of control: p = 0.66; length of follow up: p = 0.44) (Table 1). The analysis showed that dapagliflozin alone, with 18 trials involving 8,337 participants, increased the risk of UTIs (RR 1.34, 95% CI 1.11 to 1.63, I^2^ = 0%), but not with other SGLT2 inhibitors (Table 1 and Supplementary Figure 5S).

### Effects on the events suggestive of UTIs

**Figure 3:**
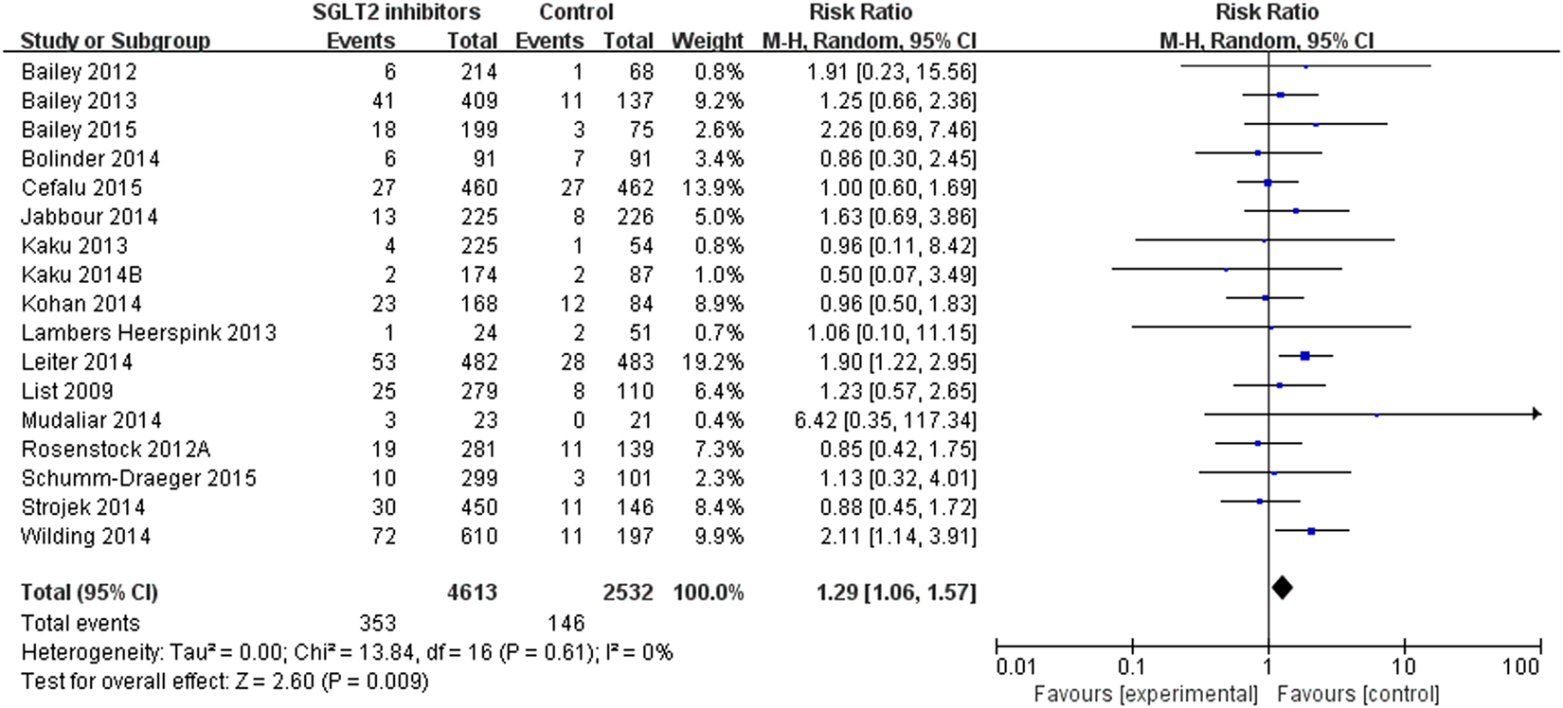
Events suggestive UTIs in type 2 diabetes patients receiving SGLT2 inhibitors versus control in randomized controlled trials.

Seventeen trials reported 499 events suggestive of UTIs in 7145 patients (raw event rate 7.0%), all assessing dapagliflozin. Meta-analysis of these trials showed that dapagliflozin was associated with higher risk of events suggestive of UTIs relative to control (RR 1.29, 95% CI 1.06 to 1.57, I^2^ = 0%; RD 91 more, 95% CI 19 more to 179 more per 1000 over 5 years) (Table 1 and Fig. 3). Sensitivity analyses did not show important changes in pooled effects. There was no evidence of publication bias for events suggestive of UTIs (Supplementary Figure 2S, Egger’s test p = 0.78). We rated the quality of evidence as high (Table 2). Subgroup analyses by type of control (interaction p = 0.44), gender (interaction p = 0.29), length of follow up (interaction p = 0.89) showed no differential effects (Supplementary Figure 9S–11S); meta-regression analyses also found no differences between effect estimates and the type of control (p = 0.50), length of follow up (p = 0.90) (Table 1).

### Effects on genital infections

**Figure 4:**
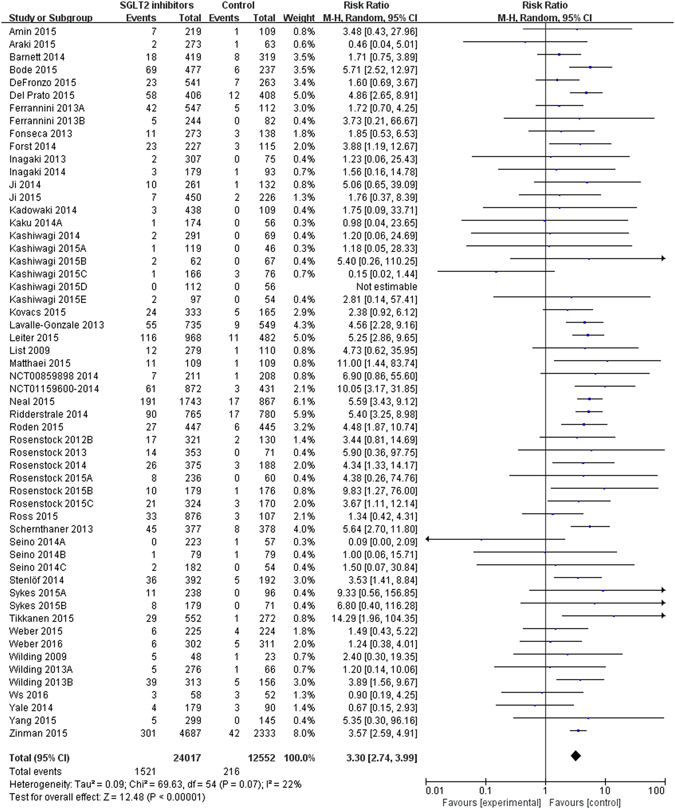
Genital infections in type 2 diabetes patients receiving SGLT2 inhibitors versus control in randomized controlled trials.

Fifty-six trials reported 1,737 genital infections events among 36,569 patients with SGLT2 inhibitors (raw event rate 4.7%). Pooling of those trials found an increased risk of genital infections for SGLT2 inhibitors (versus control: 1,521/24,017 vs. 216/12,552; RR 3.30, 95% CI 2.74 to 3.99, I^2^ = 22%; RD 423 more, 95% CI 320 more to 550 more per 1000 over 5 years) (Table 1 and Fig. 4), with a NNH of 22. There was evidence of suspected publication bias for genital infections (Supplementary Figure 3S, Egger’s test p = 0.01). Sensitivity analyses did not show important changes in pooled effects. We rated the quality of evidence as moderate due to publication bias (Table 2).

Subgroup analyses by length of follow up (interaction p = 0.005; follow up 26 weeks or shorter with RR 2.10, 95% CI 1.47 to 2.98; follow up 26–52 weeks with RR 3.26, 95% CI 2.24 to 4.74; follow up over 52 weeks with RR 4.23, 95% CI 3.36 to 5.33), type of control (interaction p = 0.04; SGLT2 inhibitors vs. placebo, RR 2.87, 95% CI 2.27 to 3.62; SGLT2 inhibitors vs. active drugs, RR 4.06, 95% CI 3.24 to 5.08), individual SGLT2 inhibitors (interaction p = 0.03) showed a statistically significant difference in genital infections (Table 1 and Supplementary Figure 12S–14S). Subgroup analyses by gender (interaction p = 0.74) showed no differential treatment effects (Table 1 and Supplementary Figure 15S). The multiple meta-regression analysis consistently showed that effects differed by length of follow up (p = 0.02), and appeared to be non-statistically different among individual SGLT2 inhibitors (p = 0.09) (Table 1). The analyses of individual drugs suggested increased risk of genital infections for canagliflozin (13 trials, RR 4.45, 95% CI 3.49 to 5.67), dapagliflozin (11 trials, RR 3.22, 95% CI 1.95 to 5.32), and empagliflozin (16 trials, RR 3.14, 95% CI 2.29 to 4.30) (Table 1 and Supplementary Figure 14S).

### Effects on events suggestive of genital infections

**Figure 5:**
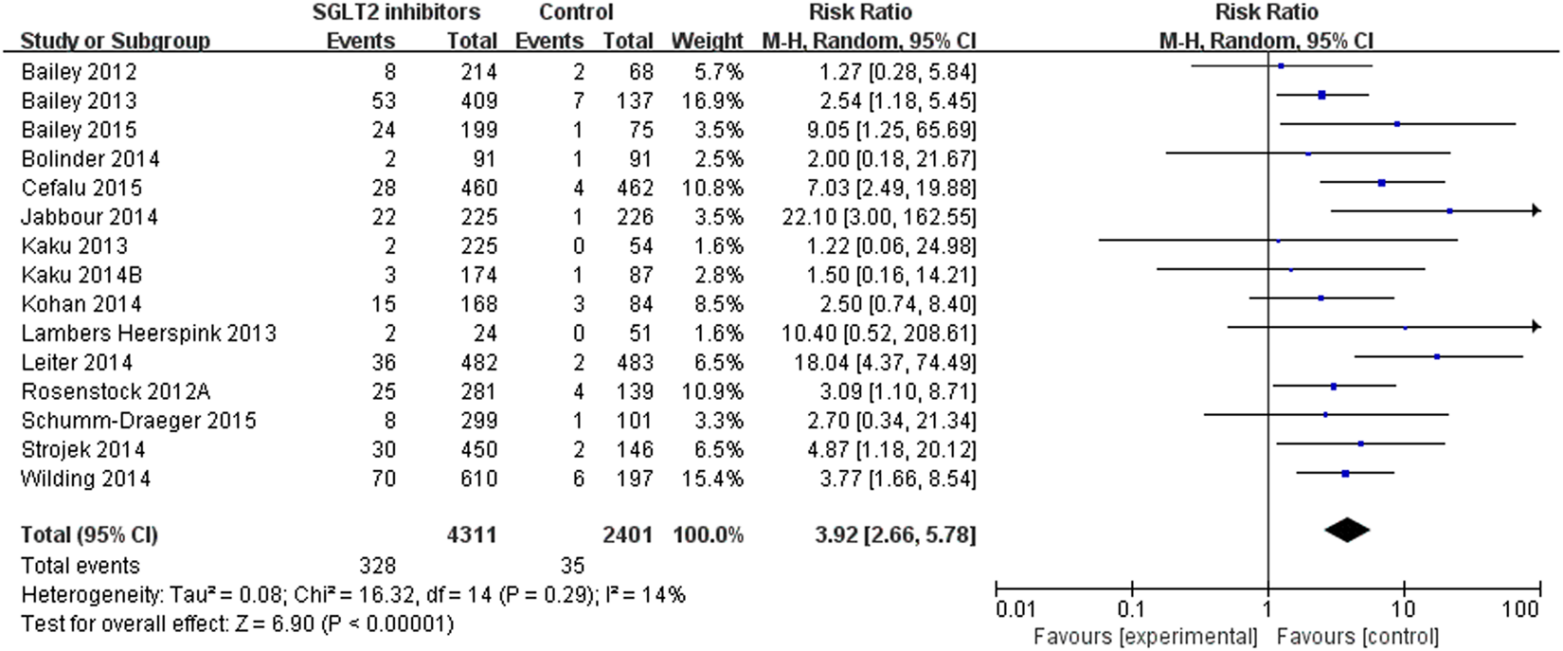
Events suggestive genital infections in type 2 diabetes patients receiving SGLT2 inhibitors versus control in randomized controlled trials

Information on events suggestive of genital infections was available in 15 trials, which reported 363 events exclusively in trials of dapagliflozin versus control (n = 6712, raw event rate 5.4%). Meta-analysis of these trials showed higher risk of events suggestive of genital infections for dapagliflozin compared with control (328/4,311 vs. 35/2,401; RR 3.92, 95% CI 2.66 to 5.78, I^2^ = 14%: RD 237 more, 95% CI 134 more to 387 more per 1000 over 5 years) (Table 1 and Fig. 5), with a NNH of 17. There was evidence of suspected publication bias for events suggestive of genital infections (Supplementary Figure 4S, Egger’s test p = 0.001). Sensitivity analyses did not show important changes in pooled effects. We rated the quality of evidence as moderate due to publication bias (Table 2).

Subgroup analyses by length of follow up (interaction p = 0.06), gender (interaction p = 0.10), type of control (interaction p = 0.83) showed no differential treatment effects (Table 1 and Supplementary Figure 16S–18S). The meta-regression analyses also showed no association between the length of follow up (p = 0.07), type of control (p = 0.50) and effect estimates (Table 1).

## Discussion

### Main findings

In this study, we found that SGLT2 inhibitors may increase the risk of genital infections. The subgroup analyses by length of follow up showed differential effects in genital infections, suggesting that the longer the use of SGLT2 inhibitors, the higher the risk of genital infections. The subgroup analysis by individual SGLT2 agents also showed that differential effects on genital infections. The above findings warrant a careful consideration of benefits and potential undesirable effects of these agents.

The current analysis has yet to establish the effect of SGLT2 inhibitors on the risk of UTIs. The finding of overall analysis was largely influenced by the inclusion of the EMPA-REG OUTCOME trial^28^, which accounted for 35% of the total weight in the analysis, and suggested no increase in UTIs with empagliflozin^31^. Removing the EMPA-REG OUTCOME trial, the pooled analysis suggested that SGLT2 inhibitors seemed to increase risk on UTIs (OR 1.10, 95% CI 1.01 to 1.19, I^2^ = 0%). Given the ongoing large clinical trials, including CANVAS (Canagliflozin Cardiovascular Assessment Study)^32^, DECLARE -TIMI58^33^ (Dapagliflozin Effect on CardiovascuLAR Events), NCT01986881^34^ (Ertugliflozin), we anticipate that inclusion of such trials when available would offer important and confirmatory insights into this issue. In the subgroup analyses on UTIs, however, we found differential effects among SGLT2 inhibitors. The analysis on the events suggestive of UTIs also supported the findings.

Using the GRADE system, we assessed the quality of evidence. We found that there was no serious limitation in consistency and directness because the evidence came from research that directly compared SGLT2 inhibitors versus placebo or active hypoglycemia drugs in type 2 diabetes patients, and the tests showed low heterogeneity. On the basis of the collective information, the quality of evidence for trials reporting UTIs was moderate as a result of failure to meet optimal information size (OIS) criteria, and the quality for trials reporting genital infections and events suggestive genital was moderate due to suspected publication bias.

In summary, the current evidence confirmed that SGLT2 inhibitors increase the risk of genital infections, and also suggested that the effects on genital infections may differ among SGLT2 inhibitors. The effects of SGLT2 on UTIs remain uncertain, and the upcoming large trials may offer confirmatory results.

### Comparison with other studies

Four previous meta-analyses^3, 4, 35, 36^ reported increased risk of both UTIs and genital infections associated with SGLT2 inhibitors. The first^35^, including 17 trials (eleven on dapagliflozin, three on canagliflozin and one on empagliflozin), reported dapagliflozin was associated with UTIs and genital infections (OR 1.32, 95% CI 1.06 to 1.63; OR 3.07, 95% CI 2.32 to 4.05). The second^4^, found that urinary tract infections and genital infections were more common with SGLT2 inhibitors compared versus placebo (21 studies, OR 1.34, 95% CI 1.03 to 1.74; 20 studies, OR 3.50, 95% CI 2.46 to 4.99) and active comparators (8 studies, OR 1.42, 95% CI 1.06 to 1.90; 8 studies, OR 5.06, 95% CI 3.44 to 7.45). The third^36^, including 13 RCTs testing SGLT2 inhibitors versus placebo with at least 52 weeks follow up, found similar results on the incidence of UTIs (OR 1.477, 95% CI 1.172 to 1.861, I^2^ = 46.6%) and genital infections (OR 5.715, 95% CI 4.339 to 7.528, I^2^ = 0.0%). The fourth^3^ included 38 RCTs, and showed an increased risk of UTIs for dapagliflozin 10 mg versus placebo and empagliflozin 25 mg, similarly increased the risk of genital infections for all SGLT2 inhibitors.

In contrast, two other meta-analyses^17, 18^ reported that, compared versus control, SGLT2 inhibitors did not increase the risk of UTIs. The first^17^, including 18 RCTs reporting UTIs, showed no statistically significant differences in the comparison of SGLT2 inhibitors with placebo (RR 1.02, 95% CI 0.54 to 1.91). The second^18^, including data from 6 regulatory submissions and 57 scientific reports respectively, for UTIs, found an increased risk in analyses of regulatory submissions data (1419/19835 in SGLT2 inhibitors vs. 690/10847 in control; RR 1.15, 95% CI 1.06 to 1.26), but not in the data from scientific reports (1852/17096 in SGLT2 inhibitors vs. 972/8965 in control; RR 1.02, 95% CI 0.95 to 1.10).

Compared with the above studies, our study included a number of additional studies and three additional SGLT2 inhibitors (ertugliflozin, remogliflozin, sotagliflozin), including 77 trials of 50820 patients. Our results regarding genital infection were generally consistent with the previous findings. However, our analyses suggested that the impact of SGLT2 inhibitors were uncertain. This inference was made primarily due to the fact that the meta-analyses results are not robust to sensitivity analysis (by excluding the major trial), the quality of evidence is moderate, and that earlier systematic reviews produced inconsistent findings. Our confidence on the effect was lowered. In addition to the data on UTIs and genital infections, we also analyzed events suggestive of UTIs and events suggestive of genital infections data in contrast to earlier systematic reviews; our findings consolidated the undesirable effects of SGLT2 inhibitors on infections.

### Strengths and Limitations

Our study has several strengths. First, in addition to journal reports, we included five unpublished trials from ClinicalTrials.gov, which provided additional outcome data. Second, we used rigorous approach to ensure the data were accurate. Particularly, we checked outcome data reported in ClincialTrials.gov versus journal reports for consistency. Third, we offered comprehensive analysis of outcomes, including UTIs, events suggestive of UTIs, genital infections and events suggestive of genital infections. The outcomes regarding events suggestive of UTIs and genital infections may provide support for our inferences. Fourth, we conducted subgroup analyses to explore the differences in risk of UTIs and genital infections and used the GRADE approach to assess quality of evidence.

Our study also has some limitations. Although the majority of trials used the same classification system for UTIs and genital infections (MedDRA), some other trials may have over-reported UTIs using simple symptoms alone. Second, for the outcomes of UTIs and genital infections, trials of dapagliflozin, canagliflozin and empagliflozin accounted the major body of evidence. The trials were, however, too few for the agents such as ertugliflozin, luseogliflozin, remogliflozin, sotagliflozin and tofogliflozin, and we thus combined those trials. The subgroup analysis examining differential effects among those agents was thus unable to disentangle the effects of those agents with very few trials. For the outcomes regarding events suggestive of UTIs and of genital infections, all the trials focused on dapagliflozin only.

## Conclusion

In summary, the current RCT evidence showed that, SGLT2 inhibitors increase the risk of genital infections, and the effects may differ among SGLT2 inhibitors and trials with different follow up. The impact of SGLT2 inhibitors on the risk of UTIs remains uncertain; the upcoming major trials may provide important insights on this issue. When their results are available, an update meta-analysis is warranted.

## Electronic supplementary material

Supplementary Figures and Tables
