# Supplementary material for: Effects of SGLT2 inhibitors on UTIs and genital infections in type 2 diabetes mellitus: a systematic review and meta-analysis
Source: Sci Rep. 2017 Jun 6;7:2824. doi: 10.1038/s41598-017-02733-w (PMC5460243; doi:10.1038/s41598-017-02733-w)
Supplement: Supplementary file 1 — Supplementary Figures and Tables [file 41598_2017_2733_MOESM1_ESM.pdf]

Effects of SGLT2 inhibitors on UTIs and genital infections in type 2 diabetes mellitus: a systematic review and meta-analysis

Jiali Liu[1], Ling Li[1], Sheyu Li[2], Pengli Jia[1], Ke Deng[1], Wenwen Chen[1] and Xin Sun[1*]

[1] Chinese Evidence-based Medicine Center, West China Hospital, Sichuan University, Chengdu, Sichuan, China

[2] Department of Endocrinology and Metabolism, West China Hospital, Sichuan University, Chengdu, Sichuan, China

[*]**Correspondence author:** Dr**.** Xin Sun, Chinese Evidence-Based Medicine Center, West China Hospital, Sichuan University, 37 Guo Xue Xiang, Chengdu 610041, Sichuan, China

Email: sunx79@hotmail.com

Table 1S. Risk of bias of randomized controlled trials of SGLT2 inhibitors treatment in type 2 diabetes mellitus

Author-Year	Adequate randomization sequence generation	Adequate allocation concealment	Blinding of participants and personnel	Blinding of outcome adjudicators	Free of incomplete outcome data
Amin-2015[1]	Definitely yes	Probably yes	Definitely yes	Probably yes	Definitely yes
Araki-2015[2,3]	Definitely yes	Definitely yes	Definitely no	Definitely no	Definitely yes
Bailey-2012[4]	Definitely yes	Definitely yes	Definitely yes	Probably yes	Definitely yes
Bailey-2013[5-7]	Definitely yes	Definitely yes	Definitely yes	Probably yes	Definitely yes
Bailey-2015[8-10]	Definitely yes	Definitely yes	Definitely yes	Probably yes	Definitely yes
Barnett-2014[11,12]	Definitely yes	Definitely yes	Definitely yes	Probably yes	Definitely yes
Bode-2015[13-15]	Definitely yes	Definitely yes	Definitely yes	Definitely yes	Definitely yes
Bolinder-2014[16-19]	Definitely yes	Definitely yes	Definitely yes	Definitely yes	Definitely yes
Cefalu-2015[20,21]	Probably yes	Probably yes	Definitely yes	Definitely yes	Probably yes
DeFronzo-2015[22-24]	Definitely yes	Definitely yes	Definitely yes	Probably yes	Probably yes
Del Prato-2015[25-28]	Definitely yes	Definitely yes	Definitely yes	Definitely yes	Definitely yes
Ferrannini-2013A[29,30]	Probably yes	Probably no	Definitely no	Definitely no	Definitely yes
Ferrannini-2013B[31,32]	Definitely yes	Definitely yes	Definitely yes	Probably yes	Definitely yes
Fonseca-2013[33]	Probably yes	Probably yes	Definitely yes	Probably yes	Probably yes
Forst-2014[34,35]	Definitely yes	Definitely yes	Definitely yes	Definitely yes	Definitely yes
Ikeda-2015[36]	Probably yes	Probably yes	Definitely yes	Probably yes	Probably yes
Inagaki-2013[37,38]	Definitely yes	Definitely yes	Definitely yes	Definitely yes	Probably yes
Inagaki-2014[39,40]	Definitely yes	Definitely yes	Definitely yes	Probably yes	Definitely yes
Jabbour-2014[41,42]	Probably yes	Probably yes	Definitely yes	Definitely yes	Definitely yes
Ji-2014[43]	Definitely yes	Definitely yes	Definitely yes	Probably yes	Definitely yes
Ji-2015[44,45]	Definitely yes	Probably yes	Definitely yes	Probably yes	Probably yes
Kadowaki-2014[46,47]	Definitely yes	Definitely yes	Definitely yes	Probably yes	Definitely yes
Kaku-2013[48,49]	Definitely yes	Definitely yes	Definitely yes	Probably yes	Definitely yes
Kaku-2014A[50]	Probably yes	Probably yes	Definitely yes	Probably yes	Definitely yes
Kaku-2014B[51]	Probably yes	Probably yes	Definitely yes	Probably yes	Definitely yes
Kashiwagi-2014[52]	Probably yes	Probably yes	Definitely yes	Probably yes	Probably yes
Kashiwagi-2015A[53]	Probably yes	Probably yes	Definitely yes	Probably yes	Definitely yes
Kashiwagi-2015B[54]	Probably yes	Probably yes	Definitely yes	Probably yes	Definitely yes
Kashiwagi-2015C[55]	Definitely yes	Probably yes	Definitely yes	Probably yes	Probably yes
Kashiwagi-2015D[56]	Probably yes	Probably yes	Definitely yes	Probably yes	Definitely yes
Kashiwagi-2015E[57]	Definitely yes	Probably yes	Definitely yes	Probably yes	Probably yes
Kohan-2014[58]	Probably yes	Probably yes	Definitely yes	Probably yes	Definitely yes
Kovacs-2015[59-61]	Definitely yes	Definitely yes	Definitely yes	Probably yes	Definitely yes
Lambers Heerspi-2013[62]	Definitely yes	Probably yes	Definitely yes	Probably yes	Definitely yes
Lavalle-Gonzale-2013[63,64]	Definitely yes	Probably yes	Definitely yes	Probably yes	Definitely yes
Leiter-2014[65,66]	Definitely yes	Definitely yes	Definitely yes	Definitely yes	Definitely yes
Leiter-2015[67-69]	Definitely yes	Definitely yes	Definitely yes	Definitely yes	Definitely yes
List-2009[70]	Probably yes	Probably yes	Definitely yes	Probably yes	Definitely yes
Matthaei-2015[71-73]	Definitely yes	Definitely yes	Definitely yes	Definitely yes	Definitely yes
Mudaliar-2014[74]	Probably yes	Probably yes	Definitely yes	Probably yes	Definitely yes
NCT00859898-2014[75]	Probably yes	Probably yes	Definitely yes	Definitely yes	Definitely yes
NCT01159600-2014[76-80]	Definitely yes	Definitely yes	Definitely yes	Probably yes	Definitely yes
NCT01340664-2014[81]	Probably yes	Probably yes	Definitely yes	Definitely yes	Definitely yes
NCT01649297-2015[82]	Probably yes	Probably yes	Definitely yes	Probably yes	Definitely yes
NCT01719003-2015[83]	Probably yes	Probably yes	Definitely yes	Probably yes	Definitely yes
NCT01809327-2015[84]	Probably yes	Probably yes	Definitely yes	Definitely yes	Definitely yes
Neal-2015[85-88]	Definitely yes	Definitely yes	Definitely yes	Probably yes	Definitely yes
Ridderstrale-2014[89,90]	Definitely yes	Definitely yes	Definitely yes	Probably yes	Probably yes
Roden-2015[91-93]	Definitely yes	Definitely yes	Definitely yes	Probably yes	Definitely yes
Rosenstock-2012A[94]	Probably yes	Probably yes	Definitely yes	Probably yes	Probably yes
Rosenstock-2012B[95-98]	Probably yes	Probably yes	Definitely yes	Definitely yes	Definitely yes

Rosenstock-2013[99,100]	Definitely yes	Definitely yes	Definitely yes	Probably yes	Definitely yes
Rosenstock-2014[101,102]	Definitely yes	Definitely yes	Definitely yes	Probably yes	Definitely yes
Rosenstock-2015A[103]	Probably yes	Probably yes	Definitely yes	Definitely yes	Definitely yes
Rosenstock-2015B[104,105]	Definitely yes	Probably yes	Definitely yes	Probably yes	Definitely yes
Rosenstock-2015C[106,107]	Definitely yes	Definitely yes	Definitely yes	Probably yes	Definitely yes
Ross-2015[108]	Probably yes	Probably yes	Definitely yes	Probably yes	Definitely yes
Schernthaner-2013[109,110]	Definitely yes	Definitely yes	Definitely yes	Probably yes	Probably yes
Schumm-Draeger-2015[111,112]	Definitely yes	Definitely yes	Definitely yes	Definitely yes	Definitely yes
Seino-2014A[113]	Probably yes	Probably yes	Definitely yes	Probably yes	Probably yes
Seino-2014B[114]	Probably yes	Probably yes	Definitely yes	Probably yes	Definitely yes
Seino-2014C[115]	Probably yes	Probably yes	Definitely yes	Probably yes	Probably yes
Stenlöf-2014[116-118]	Probably yes	Probably yes	Definitely yes	Probably yes	Definitely yes
Strojek-2014[119-121]	Definitely yes	Probably yes	Definitely yes	Definitely yes	Definitely yes
Sykes-2015A[122]	Probably yes	Probably yes	Definitely yes	Probably yes	Probably yes
Sykes-2015B[123]	Probably yes	Probably yes	Definitely yes	Probably yes	Probably yes
Tikkanen-2015[124,125]	Definitely yes	Definitely yes	Definitely yes	Probably yes	Probably yes
Weber-2015[126,127]	Definitely yes	Definitely yes	Definitely yes	Probably yes	Definitely yes
Weber-2016[128,129]	Definitely yes	Definitely yes	Definitely yes	Probably yes	Definitely yes
Wilding-2009[130]	Probably yes	Probably yes	Definitely yes	Probably yes	Definitely yes
Wilding-2013A[131]	Probably yes	Probably yes	Definitely yes	Probably yes	Definitely yes
Wilding-2013B[132,133]	Definitely yes	Definitely yes	Definitely yes	Probably yes	Definitely yes
Wilding-2014[134-136]	Definitely yes	Probably yes	Definitely yes	Definitely yes	Definitely yes
Ws-2016[137]	Probably yes	Probably no	Definitely no	Probably no	Definitely yes
Yale-2014[138-140]	Definitely yes	Definitely yes	Definitely yes	Definitely yes	Definitely yes
Yang-2015[141]	Definitely yes	Definitely yes	Definitely yes	Probably yes	Definitely yes
Zinman-2015[142,143]	Definitely yes	Definitely yes	Definitely yes	Probably yes	Probably yes

Table 2S. Baseline characteristics of included randomized controlled trials

Author-Year	International study	Number of countries involved	Number of study sites	Study phase	Total number of patients randomized	Length of follow up (weeks)	Male (n,%)	Mean age (years)	Mean BMI (kg/m²)	Mean HbA1c (%)	Mean FPG (mmol/L)	Mean diabetes duration (years)
Amin-2015[1]	Yes	5	42	II	328	12	205 (62.5)	54.4	30.4	8.1	9.1	6.3
Araki-2015[2,3]	No	1	86	III	336	52	242 (72.0)	61.3	25	8	8.5	NR
Bailey-2012[4]	Yes	7	63	III	282	24	141 (50)	53	31.8	7.9	8.8	1.4
Bailey-2013[5-7]	Yes	5	80	III	546	102	292 (53.5)	53.9	31.5	8	9.1	6.1
Bailey-2015[8-10]	Yes	4	85	III	485	102	229 (47.2)	52.6	32.6	7.9	9	0.4*
Barnett-2014[11,12]	Yes	15	127	III	741	52	430 (58)	63.9	30.7	8	8.1	NR
Bode-2015[13-15]	Yes	17	90	III	716	104	396 (55.3)	63.6	31.6	7.7	8.7	11.7
Bolinder-2014[16-19]	Yes	5	40	III	182	102	100 (55.6)	60.7	31.9	7.2	8.3	5.8
Cefalu-2015[20,21]	Yes	9	NR	III	922	52	624 (68.3)	62.9	32.8	8.1	8.8	12.4
DeFronzo-2015[22-24]	Yes	22	197	III	1363	52	721 (53.8)	55.4	31.3	8	8.7	NR
Del Prato-2015[25-28]	Yes	10	95	III	814	208	441 (55.1)	58	31.5	7.7	9.1	6.5
Ferrannini-2013A[29,30]	Yes	21	132	III	659	78	334 (50.7)	58.6	29.5	7.9	9.9	NR
Ferrannini-2013B[31,32]	Yes	13	75	II	408	12	211 (51.7)	57.5	28.5	7.9	9.7	NR
Fonseca-2013[33]	Yes	5	NR	II	412	12	211 (51.2)	53.6	30.9	7.9	9.2	4.5
Forst-2014[34,35]	Yes	11	74	III	342	52	216 (63.2)	57.4	32.5	7.9	9.2	10.5

Ikeda-2015[36]	Yes	12	NR	II	398	12	209 (52.5)	55.1	30.3	8	8.8	5.5
Inagaki-2013[37,38]	No	1	NR	II	383	12	260 (68.1)	57.4	25.7	8.09	9.2	NR
Inagaki-2014[39,40]	No	1	31	III	272	24	191 (70.5)	58	25.62	8.02	9	5.41
Jabbour-2014[41,42]	Yes	6	NR	III	451	48	245 (54.8)	54.9	NR	8	9.1	5.7
Ji-2014[43]	Yes	4	40	III	393	24	257 (65.4)	51.3	25.6	8.3	9	1.4
Ji-2015[44,45]	Yes	3	36	III	676	18	362 (53.6)	56.2	25.7	8	8.8	6.7
Kadowaki-2014[46,47]	No	1	32	II	547	12	410 (75)	57.5	25.5	7.95	8.7	NR
Kaku-2013[48,49]	No	1	26	II	279	12	215 (77.1)	57.3	NR	8.07	9	4.8
Kaku-2014A[50]	No	1	33	III	235	24	153 (66.8)	57.3	25.5	8.4	9.4	6.4
Kaku-2014B[51]	No	1	NR	III	261	24	155 (59.4)	58.8	25.4	7.5	7.7	4.9
Kashiwagi-2014[52]	No	1	39	II	361	12	233 (64.7)	55.9	25.7	8.3	10	6.7
Kashiwagi-2015A[53]	No	1	67	III	165	52	128 (78)	64.4	25.6	7.5	8.1	9.5
Kashiwagi-2015B[54]	No	1	22	III	131	16	91 (70.5)	59.4	25.5	8.3	9.7	6.7
Kashiwagi-2015C[55]	No	1	35	III	245	24	158 (65.8)	59.7	25.3	8.4	9.9	10.5
Kashiwagi-2015D[56]	No	1	34	III	169	24	99 (58.6)	56.7	25.8	8.3	9.2	7.7
Kashiwagi-2015E[57]	No	1	NR	III	152	24	112 (73.7)	56.1	27.1	8.3	9.5	6.8
Kohan-2014[58]	Yes	13	111	II	252	104	164 (65.1)	67	NR	8.3	8.8	16.9
Kovacs-2015[59-61]	Yes	8	69	III	498	76	241 (48.4)	54.5	29.2	8.1	8.43	NR
Lambers Heerspi-2013[62]	Yes	3	NR	II	75	12	49 (65.3)	56	NR	7.5	8.6	6.3
Lavalle-Gonzale-2013[63,64]	Yes	22	169	III	1284	52	605 (47.1)	55.4	31.8	7.9	9.4	6.9
Leiter-2014[65,66]	Yes	10	173	III	964	52	644 (66.9)	63.8	32.8	8.1	9.1	13.2
Leiter-2015[67-69]	Yes	19	157	III	1450	104	756 (52.1)	56.2	31	7.8	9.2	6.6
List-2009[70]	Yes	4	133	II	389	12	196 (50.4)	54.2	31.7	7.8	8.3	NR
Matthaei-2015[71-73]	Yes	6	45	III	208	52	106 (51)	61	32	8.16	9.7	9.5
Mudaliar-2014[74]	NR	NR	NR	II	44	12	29 (65.9)	54.8	NR	7.5	NR	8.2
NCT00859898-2014[75]	Yes	7	123	III	638	24	308 (48.3)	51.6	NR	9.06	NR	NR
NCT01159600-2014[76-80]	Yes	12	148	III	1307	76	700 (53.7)	56.4	28.7	8	8.5	NR
NCT01340664-2014[81]	Yes	7	60	II	279	18	130 (46.6)	57.4	NR	NR	NR	NR
NCT01649297-2015[82]	Yes	18	139	II	983	16	520 (53.9)	58.2	NR	NR	NR	NR
NCT01719003-2015[83]	Yes	22	190	III	1413	24	747 (56.3)	52.6	NR	NR	NR	NR
NCT01809327-2015[84]	Yes	12	158	III	1186	26	569 (48)	54.9	NR	NR	NR	NR
Neal-2015[85-88]	Yes	24	386	III	4330	52	2858 (66)	62.4	32.1	8.2	9.3	13.4
Ridderstrale-2014[89,90]	Yes	23	173	III	1549	104	853 (55.2)	55.9	30.2	7.9	8.3	NR

Roden-2015[91-93]	Yes	9	124	III	899	76	551 (61.3)	55	28.4	7.88	8.42	NR
Rosenstock-2012A[94]	Yes	7	105	III	420	48	208 (49.5)	53.5	NR	8.4	9.1	5.5
Rosenstock-2012B[95-98]	Yes	12	85	II	451	12	233 (51.7)	52.9	31.5	7.75	9	6
Rosenstock-2013[99,100]	Yes	16	104	II	495	12	250 (50.5)	58.3	31.4	8	9.8	NR
Rosenstock-2014[101,102]	Yes	14	104	III	563	52	256 (45.5)	56.7	34.8	8.3	8.52	NR
Rosenstock-2015A[103]	No	1	52	II	299	12	135 (45.2)	55.9	33.1	8.1	9.6	NR
Rosenstock-2015B[104,105]	Yes	9	NR	III	534	24	268 (50.2)	54	31.7	8.94	10.3	7.6
Rosenstock-2015C[106,107]	Yes	7	97	II	494	78	276 (55.9)	58.8	32.2	8.2	7.9	NR
Ross-2015[108]	Yes	20	52	II	983	16	520 (53.9)	58.2	31.8	7.77	8.9	NR
Schernthaner-2013[109,110]	Yes	17	140	III	756	52	422 (55.8)	56.7	31.6	8.1	9.3	9.6
Schumm-Draeger-2015[111,112]	Yes	7	53	III	400	16	179 (44.9)	57.7	32.6	7.8	8.6	5.2
Seino-2014A[113]	No	1	41	II	282	12	197 (70.4)	58	24.8	7.9	8.6	5
Seino-2014B[114]	No	1	23	III	158	24	116 (73.4)	59.3	25.7	8.2	9	6.3
Seino-2014C[115]	No	1	40	II	239	12	160 (67.8)	57	25	8.1	8.8	6
Stenlöf-2014[116-118]	Yes	17	90	III	587	52	258 (44)	55.4	31.6	8	9.5	4.3
Strojek-2014[119-121]	Yes	7	84	III	597	48	285(48.1)	59.8	29.84	8.11	9.6	7.4
Sykes-2015A[122]	Yes	19	136	II	336	12	188 (58.2)	54.6	31.01	8.12	NR	2.34
Sykes-2015B[123]	Yes	16	NR	II	252	12	118 (49)	52.9	31.4	8.03	NR	2.61
Tikkanen-2015[124,125]	Yes	12	NR	III	825	12	495(60.1)	60.2	32.6	7.9	8.9	NR
Weber-2015[126,127]	Yes	16	311	III	588	12	247 (55.0)	56.5	NR	8	9	7.5
Weber-2016[128,129]	Yes	15	418	III	613	12	350 (57.1)	55.9	NR	8	8.9	7.9
Wilding-2009[130]	Yes	2	26	II	71	12	42 (59.2)	56.7	35.5	8.4	9	12.3
Wilding-2013A[131]	Yes	6	45	II	343	12	175 (51)	57.4	31.7	7.8	8.6	5.9
Wilding-2013B[132,133]	Yes	11	85	III	469	52	239 (51)	56.8	33.1	8.1	9.5	9.6
Wilding-2014[134-136]	Yes	30	126	III	808	104	382 (47.8)	59.3	33.11	8.53	9.9	13.6
Ws-2016[137]	No	1	NR	NR	110	12	66 (60)	54.4	29.8	7.7	8.1	5.5
Yale-2014[138-140]	Yes	19	89	III	272	52	163 (59.9)	68.5	33	8	9.1	16.3
Yang-2015[141]	Yes	3	32	III	445	24	445 (54.3)	53.8	26.1	8.1	9.1	4.9
Zinman-2015[142,143]	Yes	42	590	III	7028	161[#]	5016 (74.5)	63.1	30.6	8.1	8.5	NR

BMI=body mass index; FPG=fasting plasma glucose; NR=not reported.

[*]median diabetes duration (years); [#]median follow up time (weeks).

Table 3S. Interventions tested and event rates in randomized controlled trials in patients with type 2 diabetes mellitus

Author (year)	Medications used across groups	Incretin		Control		Duration of treatment (weeks)
		Type	Events	Type	Events	

Trials reporting urinary tract infections						
Amin-2015[1]	Metformin	Ertugliflozin	7/219	Placebo	4/54	12
		Ertugliflozin	7/219	Sitagliptin	1/55	
Araki-2015[2,3]	SU	Empagliflozin	12/273	Metformin	2/63	52
Bailey-2013[5-7]	Metformin	Dapagliflozin	30/409	Placebo	8/137	102
Barnett-2014[11,12]	Insulin or any combination	Empagliflozin	61/419	Placebo	47/319	52
Bode-2015[13-15]	Stable antihyperglycemic (AHA) regimen	Canagliflozin	74/477	Placebo	24/237	104
Bolinder-2014[16-19]	Metformin	Dapagliflozin	3/91	Placebo	4/91	102
Cefalu-2015[20-21]	None	Dapagliflozin	23/460	Placebo	22/462	52
DeFronzo-2015[22-24]	Metformin ± linagliptin	Empagliflozin	65/541	No additional drugs	34/263	52
	None	Empagliflozin	71/547	Linagliptin	34/263	
Del Prato-2015[25-28]	Metformin	Dapagliflozin	55/406	Glipizide	38/408	208
Ferrannini-2013A[29-30]	None	Empagliflozin	11/215	Metformin	2/56	78
		Empagliflozin	36/332	Sitagliptin	7/56	
Ferrannini-2013B[31,32]	None	Empagliflozin	4/244	Placebo	1/82	12
Fonseca-2013[33]	None	Ipragliflozin	21/273	Placebo	6/69	12
		Ipragliflozin	21/273	Metformin	5/69	
Forst-2014[34,35]	Metformin + pioglitazone	Canagliflozin	15/227	Sitagliptin	9/115	52
Ikeda-2015[36]	Metformin	Tofogliflozin	8/328	Placebo	1/66	12
Inagaki-2013[37,38]	None	Canagliflozin	0/307	Placebo	0/75	12
Inagaki-2014[39,40]	None	Canagliflozin	2/179	Placebo	1/93	24
Ji-2014[43]	None	Dapagliflozin	12/261	Placebo	4/132	24
Ji-2015[44,45]	Metformin ± SU	Canagliflozin	13/450	Placebo	11/226	18
Kadowaki-2014[46,47]	None	Empagliflozin	3/438	Placebo	1/109	12
Kaku-2014A[50]	None	Tofogliflozin	1/174	Placebo	0/56	24
Kashiwagi-2014[52]	None	Ipragliflozin	4/291	Placebo	1/69	12
Kashiwagi-2015A[53]	None	Ipragliflozin	1/119	Placebo	2/46	52
Kashiwagi-2015B[54]	None	Ipragliflozin	0/62	Placebo	1/67	16
Kashiwagi-2015C[55]	SU	Ipragliflozin	2/166	Placebo	3/76	24
Kashiwagi-2015D[56]	Metformin	Ipragliflozin	2/112	Placebo	2/56	24
Kashiwagi-2015E[57]	Pioglitazone	Ipragliflozin	3/97	Placebo	1/54	24
Kovacs-2015[59-61]	Pioglitazone ± metformin	Empagliflozin	74/333	Placebo	44/165	76
Lavalle-Gonzale-2013[63,64]	Metformin	Canagliflozin	47/735	Sitagliptin	35/549	52
Leiter-2014[65,66]	None	Dapagliflozin	40/482	Placebo	23/483	52
Leiter-2015[67-69]	Metfoimin	Canagliflozin	93/968	Glimepiride	33/482	104
List-2009[70]	None	Dapagliflozin	21/279	Placebo	3/54	12
		Dapagliflozin	21/279	Metformin	4/56	
Matthaei-2015[71-73]	Metformin ± SU	Dapagliflozin	11/109	Placebo	12/109	52
Mudaliar-2014[74]	Metformin ± insulin secretagogue	Dapagliflozin	2/23	Placebo	0/21	12
NCT00859898-2014[75]	Metformin XR	Dapagliflozin	6/211	No additional drugs	4/208	24
	None	Dapagliflozin	17/219	Metformin XR	4/208	
NCT01159600-2014[76-80]	Metformin + SU	Empagliflozin	126/872	Placebo	64/431	76
NCT01340664-2014[81]	None	Canagliflozin	1/186	Placebo	0/93	18
NCT01649297-2015[82]	None	Empagliflozin	43/876	Placebo	4/107	16
NCT01719003-2015[83]	Metformin	Empagliflozin	56/680	Placebo	26/341	24
	None	Empagliflozin	25/339	Metformin	26/341	
NCT01809327-2015[84]	Metformin XR	Canagliflozin	9/474	No additional drugs	3/237	26
	None	Canagliflozin	7/475	Metformin XR	3/237	
Neal-2015[85-88]	Insulin Sulfonylurea incretin-mimetic therapy	Canagliflozin	102/1743	Placebo	44/867	52
Ridderstrale-2014[89,90]	Metformin	Empagliflozin	105/765	Glimepiride	102/780	104
Roden-2015[91-93]	None	Empagliflozin	41/447	Placebo	25/229	76
		Empagliflozin	41/447	Sitagliptin	20/223	
Rosenstock-2012B[95-98]	Metformin	Canagliflozin	16/321	Placebo	4/65	12
		Canagliflozin	16/321	Sitagliptin	1/65	
Rosenstock-2013[99,100]	Metformin	Empagliflozin	14/353	Placebo	2/71	12
Rosenstock-2014[101,102]	MDI insulin ± metformin	Empagliflozin	58/375	Placebo	29/188	52
Rosenstock-2015A[103]	Metformin	Sotagliflozin	4/236	Placebo	1/60	12
Rosenstock-2015B[104,105]	Saxagliptin + metfomin	Dapagliflozin	1/179	No additional drugs	9/176	24
	Metformin	Dapagliflozin	7/179	Saxagliptin	9/176	

Rosenstock-2015C[106,107]	Basal insulin ± metformin SU	Empagliflozin	43/324	Placebo	15/170	78
Ross-2015[108]	Metformin immediate release	Empagliflozin	63/876	Placebo	4/107	16
Schernthaner-2013[109,110]	Metformin + SU	Canagliflozin	15/377	Sitagliptin	21/378	52
Seino-2014A[113]	None	Luseogliflozin	1/223	Placebo	0/57	12
Seino-2014B[114]	None	Luseogliflozin	0/79	Placebo	0/79	24
Seino-2014C[115]	None	Luseogliflozin	1/182	Placebo	0/54	12
Stenlöf-2014[116-118]	None	Canagliflozin	30/392	Sitagliptin	12/192	52
Strojek-2014[119-121]	Glimepiride	Dapagliflozin	15/450	Placebo	7/146	48
Sykes-2015A[122]	None	Remogliflozin	3/238	Placebo	0/48	12
		Remogliflozin	3/238	Pioglitazone	4/48	
Sykes-2015B[123]	None	Remogliflozin	10/179	Placebo	0/36	12
		Remogliflozin	10/179	Pioglitazone	2/35	
Tikkanen-2015[124,125]	None	Empagliflozin	24/552	Placebo	10/272	12
Weber-2015[126,127]	Antihypertensive drug	Dapagliflozin	4/225	Placebo	2/224	12
Weber-2016[128,129]	Antidiabetes agents	Dapagliflozin	9/302	Placebo	3/311	12
Wilding-2009[130]	Insulin oral antidiabetic agents	Dapagliflozin	1/48	Placebo	0/23	12
Wilding-2013A[131]	Metformin	Ipragliflozin	12/276	Placebo	4/66	12
Wilding-2013B[132,133]	Metformin + SU	Canagliflozin	26/313	Placebo	12/156	52
Wilding-2014[134-136]	Insulin	Dapagliflozin	49/610	Placebo	7/197	104
Ws-2016[137]	Metformin	Dapagliflozin	6/58	SU	2/52	12
Yale-2014[138-140]	AHA regimens	Canagliflozin	18/179	Placebo	9/90	52
Yang-2015[141]	Metformin	Dapagliflozin	16/299	Placebo	7/145	24
Zinman-2015[142,143]	None	Empagliflozin	842/4687	Placebo	423/2333	161#
**Trials reporting events suggestive of urinary tract infections**						
Bailey-2012[4]	None	Dapagliflozin	6/214	Placebo	1/68	24
Bailey-2013[5-7]	Metformin	Dapagliflozin	41/409	Placebo	11/137	102
Bailey-2015[8-10]	None	Dapagliflozin	18/199	Placebo	3/75	102
Bolinder-2014[16-19]	Metformin	Dapagliflozin	6/91	Placebo	7/91	102
Cefalu-2015[20,21]	None	Dapagliflozin	27/460	Placebo	27/462	52
Jabbour-2014[41,42]	Sitagliptin ± metformin	Dapagliflozin	13/225	Placebo	8/226	48
Kaku-2013[48,49]	None	Dapagliflozin	4/225	Placebo	1/54	12
Kaku-2014B[51]	None	Dapagliflozin	2/174	Placebo	2/87	24
Kohan-2014[58]	None	Dapagliflozin	23/168	Placebo	12/84	104
Lambers Heerspink-2013[62]	None	Dapagliflozin	1/24	Placebo	0/25	12
		Dapagliflozin	1/24	Hydrochlorothiazide	2/26	
Leiter-2014[65,66]	None	Dapagliflozin	53/482	Placebo	28/483	52
List-2009[70]	None	Dapagliflozin	25/279	Placebo	3/54	12
		Dapagliflozin	25/279	Metformin	5/56	
Mudaliar-2014[74]	Metformin ± insulin secretagogue	Dapagliflozin	3/23	Placebo	0/21	12
Rosenstock-2012A[94]	Pioglitazone	Dapagliflozin	19/281	Placebo	11/139	48
Schumm-Draeger-2015[111,112]	Metformin	Dapagliflozin	10/299	Placebo	3/101	16
Strojek-2014[119-121]	Glimepiride	Dapagliflozin	30/450	Placebo	11/146	48
Wilding-2014[134-136]	Insulin	Dapagliflozin	72/610	Placebo	11/197	104
**Trials reporting genital infections**						
Amin-2015[1]	Metformin	Ertugliflozin	7/219	Placebo	1/54	12
		Ertugliflozin	7/219	Sitagliptin	0/55	
Araki-2015[2,3]	SU	Empagliflozin	2/273	Metformin	1/63	52
Barnett-2014[11,12]	Insulin or any combination	Empagliflozin	18/419	Placebo	8/319	52
Bode-2015[13-15]	Stable antihyperglycemic (AHA) regimen	Canagliflozin	69/477	Placebo	6/237	104
DeFronzo-2015[22-24]	Metformin ± linagliptin	Empagliflozin	23/541	No additional drugs	7/263	52
	None	Empagliflozin	36/547	Linagliptin	7/263	
Del Prato-2015[25-28]	Metformin	Dapagliflozin	58/406	Glipizide	12/408	208
Ferrannini-2013A[29,30]	None	Empagliflozin	11/215	Metformin	1/56	78
		Empagliflozin	31/332	Sitagliptin	4/56	
Ferrannini-2013B[31,32]	None	Empagliflozin	5/244	Placebo	0/82	12
Fonseca-2013[33]	None	Ipragliflozin	11/273	Placebo	1/69	12
		Ipragliflozin	11/273	Metformin	2/69	
Forst-2014[34,35]	Metformin + pioglitazone	Canagliflozin	23/227	Sitagliptin	3/115	52
Inagaki-2013[37,38]	None	Canagliflozin	2/307	Placebo	0/75	12

Inagaki-2014[39,40]	None	Canagliflozin	3/179	Placebo	1/93	24
Ji-2014[43]	None	Dapagliflozin	10/261	Placebo	1/132	24
Ji-2015[44,45]	Metformin ± SU	canagliflozin	7/450	Placebo	2/226	18
Kadowaki-2014[46,47]	None	Empagliflozin	3/438	Placebo	0/109	12
Kaku-2014A[50]	None	Tofogliflozin	1/174	Placebo	0/56	24
Kashiwagi-2014[52]	None	Ipragliflozin	2/291	Placebo	0/69	12
Kashiwagi-2015A[53]	None	Ipragliflozin	1/119	Placebo	0/46	52
Kashiwagi-2015B[54]	None	Ipragliflozin	2/62	Placebo	0/67	16
Kashiwagi-2015C[55]	SU	Ipragliflozin	1/166	Placebo	3/76	24
Kashiwagi-2015D[56]	Metformin	Ipragliflozin	0/112	Placebo	0/56	24
Kashiwagi-2015E[57]	Pioglitazone	Ipragliflozin	2/97	Placebo	0/54	24
Kovacs-2015[59-61]	Pioglitazone ± metformin	Empagliflozin	24/333	Placebo	5/165	76
Lavalle-Gonzale-2013[62]	Metformin	Canagliflozin	55/735	Sitagliptin	9/549	52
Leiter-2015[67-69]	Metfoimin	Canagliflozin	116/968	Glimepiride	11/482	104
		Dapagliflozin	12/279	Placebo	0/54	12
List-2009[70]	None	Dapagliflozin	12/279	Metformin	1/56	
Matthaei-2015[71-73]	Metformin ± SU	Dapagliflozin	11/109	Placebo	1/109	52
	Metformin XR	Dapagliflozin	7/211	No additional drugs	1/208	24
NCT00859898-2014[75]	None	Dapagliflozin	11/219	Metformin XR	1/208	
NCT01159600-2014[76-80]	Metformin + SU	Empagliflozin	61/872	Placebo	3/431	76
Neal-2015[85-88]	Insulin Sulfonylurea incretin-mimetic therapy	Canagliflozin	191/1743	Placebo	17/867	52
Ridderstrale-2014[89,90]	Metformin	Empagliflozin	90/765	Glimepiride	17/780	104
		Empagliflozin	27/447	Placebo	4/229	76
Roden-2015[91-93]	None	Empagliflozin	27/447	Sitagliptin	2/223	
		Canagliflozin	17/321	Placebo	1/65	12
Rosenstock-2012B[95-98]	Metformin	Canagliflozin	17/321	sitagliptin	1/65	
Rosenstock-2013[99,100]	Metformin	Empagliflozin	14/353	Placebo	0/71	12
Rosenstock-2014[101,102]	MDI insulin ± metformin	Empagliflozin	26/375	Placebo	3/188	52
Rosenstock-2015A[103]	Metformin	Sotagliflozin	8/236	Placebo	0/60	12
	Saxagliptin + metfomin	Dapagliflozin	0/179	No additional drugs	1/176	24
Rosenstock-2015B[104,105]	Metformin	Dapagliflozin	10/179	Saxagliptin	1/176	
Rosenstock-2015C[106,107]	Basal insulin ± metformin SU	Empagliflozin	21/324	Placebo	3/170	78
Ross-2015[108]	Metformin immediate release	Empagliflozin	33/876	Placebo	3/107	16
Schernthaner-2013[109,110]	Metformin + SU	Canagliflozin	45/377	Sitagliptin	8/378	52
Seino-2014A[113]	None	Luseogliflozin	0/223	Placebo	1/57	12
Seino-2014B[114]	None	Luseogliflozin	1/79	Placebo	1/79	24
Seino-2014C[115]	None	Luseogliflozin	2/182	Placebo	0/54	12
Stenlöf-2014[116-118]	None	Canagliflozin	36/392	Sitagliptin	5/192	52
		Remogliflozin	11/238	Placebo	0/48	12
Sykes-2015A[122]	None	Remogliflozin	11/238	Pioglitazone	0/48	
		Remogliflozin	8/179	Placebo	0/36	12
Sykes-2015B[123]	None	Remogliflozin	8/179	Pioglitazone	0/35	
Tikkanen-2015[124,125]	None	Empagliflozin	29/552	Placebo	1/272	12
Weber-2015[126,127]	Antihypertensive drug	Dapagliflozin	6/225	Placebo	4/224	12
Weber-2016[128,129]	Antidiabetes agents	Dapagliflozin	6/302	Placebo	5/311	12
Wilding-2009[130]	Insulin oral antidiabetic agents	Dapagliflozin	5/48	Placebo	1/23	12
Wilding-2013A[131]	Metformin	Ipragliflozin	5/276	Placebo	1/66	12
Wilding-2013B[132,133]	Metformin + SU	Canagliflozin	39/313	Placebo	5/156	52
Ws-2016[137]	Metformin	Dapagliflozin	3/58	SU	3/52	12
Yale-2014[138-140]	AHA regimens	Canagliflozin	4/179	Placebo	3/90	52
Yang-2015[141]	Metformin	Dapagliflozin	5/299	Placebo	0/145	24
Zinman-2015[141]	None	Empagliflozin	301/4687	Placebo	42/2333	161[#]
**Trials reporting events suggestive of genital infections**						
Bailey-2012[4]	None	Dapagliflozin	8/214	Placebo	2/68	24
Bailey-2013[5-7]	Metformin	Dapagliflozin	53/409	Placebo	7/137	102
Bailey-2015[8-10]	None	Dapagliflozin	24/199	Placebo	1/75	102
Bolinder-2014[16-19]	Metformin	Dapagliflozin	2/91	Placebo	1/91	102
Cefalu-2015[22-24]	None	Dapagliflozin	28/460	Placebo	4/462	52

Jabbour-2014[41,42]	Sitagliptin ± metformin	Dapagliflozin	22/225	Placebo	1/226	48
Kaku-2013[48,49]	None	Dapagliflozin	2/225	Placebo	0/54	12
Kaku-2014B[51]	None	Dapagliflozin	3/174	Placebo	1/87	24
Kohan-2014[58]	None	Dapagliflozin	15/168	Placebo	3/84	104
Lambers Heerspink-2013[62]	None	Dapagliflozin	2/24	Placebo	0/25	12
		Dapagliflozin	2/24	Hydrochlorothiazide	0/26	
Leiter-2014[65,66]	None	Dapagliflozin	36/482	Placebo	2/483	52
Rosenstock-2012A[94]	Pioglitazone	Dapagliflozin	25/281	Placebo	4/139	48
Schumm-Draeger-2015[111,112]	Metformin	Dapagliflozin	8/299	Placebo	1/101	16
Strojek-2014[119-121]	Glimepiride	Dapagliflozin	30/450	Placebo	2/146	48
Wilding-2014[134-136]	Insulin	Dapagliflozin	70/610	Placebo	6/197	104

SU=sulphonylurea; #median follow up time (weeks)

Reference of included trials

1 Amin, N. B. *et al.* Dose-ranging efficacy and safety study of ertugliflozin, a sodium-glucose co-transporter 2 inhibitor, in patients with type 2 diabetes on a background of metformin. *Diabetes, obesity & metabolism.* **17,** 591-598 (2015).

2 Araki, E. *et al.* Long-term treatment with empagliflozin as add-on to oral antidiabetes therapy in Japanese patients with type 2 diabetes mellitus. *Diabetes, obesity & metabolism.* **17,** 665-674 (2015).

3 Eli Lilly and Company, Boehringer Ingelheim. Empagliflozin (BI 10773) Comprehensive add-on Study in Japanese Subjects With Type 2 Diabetes Mellitus. National Library of Medicine (US) http://clinicaltrials.gov/show/NCT01368081 (2014). (Date of access: 10 November 2016).

4 Bailey, C. J., Iqbal, N., T'Joen, C. & List, J. F. Dapagliflozin monotherapy in drug-naive patients with diabetes: a randomized-controlled trial of low-dose range. *Diabetes, obesity & metabolism.* **14,** 951-959 (2012).

5 Bailey, C. J. *et al.* Dapagliflozin add-on to metformin in type 2 diabetes inadequately controlled with metformin: a randomized, double-blind, placebo-controlled 102-week trial. *BMC medicine.* **11,** 43 (2013).

6 Bailey, C. J., Gross, J. L., Pieters, A., Bastien, A. & List, J. F. Effect of dapagliflozin in patients with type 2 diabetes who have inadequate glycaemic control with metformin: a randomised, double-blind, placebo-controlled trial. *Lancet (London, England).* **375,** 2223-2233 (2010).

7 Bristol-Myers Squibb, AstraZeneca. A Phase III Study of BMS-512148 (Dapagliflozin) in Patients With Type 2 Diabetes Who Are Not Well Controlled on Metformin Alone. National Library of Medicine (US) http://clinicaltrials.gov/show/NCT00528879 (2014). (Date of access: 10 November 2016).

8 Bailey, C. J. *et al.* Efficacy and safety of dapagliflozin monotherapy in people with Type 2 diabetes: a randomized double-blind placebo-controlled 102-week trial. *Diabetic medicine : a journal of the British Diabetic Association.* **32,** 531-541 (2015).

9 Ferrannini, E., Ramos, S. J., Salsali, A., Tang, W. & List, J. F. Dapagliflozin monotherapy in type 2 diabetic patients with inadequate glycemic control by diet and exercise: a randomized, double-blind, placebo-controlled, phase 3 trial. *Diabetes care.* **33,** 2217-2224 (2010).

10 Bristol-Myers Squibb, AstraZeneca. A Phase III Study of BMS-512148 (Dapagliflozin) in Patients With Type 2 Diabetes Who Are Not Well Controlled With Diet and Exercise. National Library of Medicine (US) http://clinicaltrials.gov/show/NCT00528372 (2014). (Date of access: 10 November 2016).

11 Barnett, A. H. *et al.* Efficacy and safety of empagliflozin added to existing antidiabetes treatment in patients with type 2 diabetes and chronic kidney disease: a randomised, double-blind, placebo-controlled trial. *The lancet. Diabetes & endocrinology.* **2,** 369-384 (2014).

12 Eli Lilly and Company, Boehringer Ingelheim. Efficacy and Safety of Empagliflozin (BI 10773) in Patients With Type 2 Diabetes and Renal Impairment. National Library of Medicine (US) http://clinicaltrials.gov/show/NCT01368081 (2014). (Date of access: 10 November 2016).

13 Bode, B. *et al.* Long-term efficacy and safety of canagliflozin over 104 weeks in patients aged 55-80 years with type 2 diabetes. *Diabetes, obesity & metabolism.* **17,** 294-303 (2015).

14 Bode, B., Stenlof, K., Sullivan, D., Fung, A. & Usiskin, K. Efficacy and safety of canagliflozin treatment in older subjects with type 2 diabetes mellitus: a randomized trial. *Hospital practice (1995).* **41,** 72-84 (2013).

15 Janssen Research & Development, LLC. A Safety and Efficacy Study of Canagliflozin in Older Patients (55 to 80 Years of Age) With Type 2 Diabetes Mellitus. National Library of Medicine (US) http://clinicaltrials.gov/show/NCT01368081 (2014). (Date of access: 10 November 2016).

16 Bolinder, J. *et al.* Dapagliflozin maintains glycaemic control while reducing weight and body fat mass over 2 years in patients with type 2 diabetes mellitus inadequately controlled on metformin. *Diabetes, obesity & metabolism.* **16,** 159-169 (2014).

17 Bolinder, J. *et al.* Effects of dapagliflozin on body weight, total fat mass, and regional adipose tissue distribution in patients with type 2 diabetes mellitus with inadequate glycemic control on metformin. *The Journal of clinical endocrinology and metabolism.* **97,** 1020-1031 (2012).

18 Ljunggren, O. *et al.* Dapagliflozin has no effect on markers of bone formation and resorption or bone mineral density in patients with inadequately controlled type 2 diabetes mellitus on metformin. *Diabetes, obesity & metabolism.* **14,** 990-999 (2012).

19 Bristol-Myers Squibb, AstraZeneca. Evaluation of the Effect of Dapagliflozin in Combination With Metformin on Body Weight in Subjects With Type 2 Diabetes. National Library of Medicine (US) http://clinicaltrials.gov/show/NCT00855166 (2013). (Date of access: 10 November 2016).

20 Cefalu, W. T. *et al.* Dapagliflozin's Effects on Glycemia and Cardiovascular Risk Factors in High-Risk Patients With Type 2 Diabetes: A 24-Week, Multicenter, Randomized, Double-Blind, Placebo-Controlled Study With a 28-Week Extension. *Diabetes care.* **38,** 1218-1227 (2015).

21 Bristol-Myers Squibb, AstraZeneca. Efficacy and Safety in Patients With Type 2 Diabetes Mellitus, Cardiovascular Disease and Hypertension. National Library of Medicine (US) http://clinicaltrials.gov/show/NCT01031680 (2013). (Date of access: 10 November 2016).

22 DeFronzo, R. A. *et al.* Combination of empagliflozin and linagliptin as second-line therapy in subjects with type 2 diabetes inadequately controlled on metformin. *Diabetes care.* **38,** 384-393 (2015).

23 Lewin, A. *et al.* Initial combination of empagliflozin and linagliptin in subjects with type 2 diabetes. *Diabetes care.* **38,** 394-402 (2015).

24 Eli Lilly and Company, Boehringer Ingelheim. Efficacy and Safety of Empagliflozin (BI 10773) / Linagliptin (BI 1356) Fixed Dose Combination in Treatment naïve and Metformin Treated Type 2 Diabetes Patients. National Library of Medicine (US) http://clinicaltrials.gov/show/NCT01422876 (2015). (Date of access: 10 November 2016).

25 Del Prato, S. *et al.* Long-term glycaemic response and tolerability of dapagliflozin versus a sulphonylurea as add-on therapy to metformin in patients with type 2 diabetes: 4-year data. *Diabetes, obesity & metabolism.* **17,** 581-590 (2015).

26 Nauck, M. A. *et al.* Durability of glycaemic efficacy over 2 years with dapagliflozin versus glipizide as add-on therapies in patients whose type 2 diabetes mellitus is inadequately controlled with metformin. *Diabetes, obesity & metabolism.* **16,** 1111-1120 (2014).

27 Nauck, M. A. *et al.* Dapagliflozin versus glipizide as add-on therapy in patients with type 2 diabetes who have inadequate glycemic control with metformin: a randomized, 52-week, double-blind, active-controlled noninferiority trial. *Diabetes care.* **34,** 2015-2022 (2011).

28 Bristol-Myers Squibb, AstraZeneca. Efficacy and Safety of Dapagliflozin in Combination With Metformin in Type 2 Diabetes Patients. National Library of Medicine (US) http://clinicaltrials.gov/show/NCT00660907 (2013). (Date of access: 10 November 2016).

29 Ferrannini, E. *et al.* Long-term safety and efficacy of empagliflozin, sitagliptin, and metformin: an active-controlled, parallel-group, randomized, 78-week open-label extension study in patients with type 2 diabetes. *Diabetes care.* **36,** 4015-4021 (2013).

30 Boehringer Ingelheim. Empagliflozin (BI 10773) in Type Two Diabetes (T2D) Patients, Open Label Extension. National Library of Medicine (US) http://clinicaltrials.gov/show/NCT00881530 (2014). (Date of access: 10 November 2016).

31 Ferrannini, E. *et al.* A Phase IIb, randomized, placebo-controlled study of the SGLT2 inhibitor empagliflozin in patients with type 2 diabetes. *Diabetes, obesity & metabolism.* **15,** 721-728 (2013).

32 Boehringer Ingelheim. 12 Weeks Treatment With 3 Different Doses of BI 10773 in Type 2 Diabetic Patients. National Library of Medicine (US) http://clinicaltrials.gov/show/NCT00789035 (2014). (Date of access: 10 November 2016).

33 Fonseca, V. A. *et al.* Active- and placebo-controlled dose-finding study to assess the efficacy, safety, and tolerability of multiple doses of ipragliflozin in patients with type 2 diabetes mellitus. *Journal of diabetes and its complications.* **27,** 268-273 (2013).

34 Forst, T. *et al.* Efficacy and safety of canagliflozin over 52 weeks in patients with type 2 diabetes on background metformin and pioglitazone. *Diabetes, obesity & metabolism.* **16,** 467-477 (2014).

35 Janssen Research & Development, LLC. The CANTATA-MP Trial (CANagliflozin Treatment and Trial Analysis - Metformin and Pioglitazone). National Library of Medicine (US) http://clinicaltrials.gov/show/NCT01106690 (2013). (Date of access: 10 November 2016).

36 Ikeda, S. *et al.* A novel and selective sodium-glucose cotransporter-2 inhibitor, tofogliflozin, improves glycaemic control and lowers body weight in patients with type 2 diabetes mellitus. *Diabetes, obesity & metabolism.* **17,** 984-993 (2015).

37 Inagaki, N. *et al.* Efficacy and safety of canagliflozin in Japanese patients with type 2 diabetes: a randomized, double-blind, placebo-controlled, 12-week study. *Diabetes, obesity & metabolism.* **15,** 1136-1145 (2013).

38 Mitsubishi Tanabe Pharma Corporation. An Efficacy, Safety, and Tolerability Study for TA-7284 in Patients With Type 2 Diabetes. National Library of Medicine (US) http://clinicaltrials.gov/show/NCT01022112 (2014). (Date of access: 10 November 2016).

39 Inagaki, N. *et al.* Efficacy and safety of canagliflozin monotherapy in Japanese patients with type 2 diabetes inadequately controlled with diet and exercise: a 24-week, randomized, double-blind, placebo-controlled, Phase III study. *Expert opinion on pharmacotherapy.* **15,** 1501-1515 (2014).

40 Mitsubishi Tanabe Pharma Corporation. Efficacy and Safety Study of TA-7284 in Patients With Type 2 Diabetes. National Library of Medicine (US) http://clinicaltrials.gov/show/NCT01413204 (2014). (Date of access: 10 November 2016).

41 Jabbour, S. A., Hardy, E., Sugg, J. & Parikh, S. Dapagliflozin is effective as add-on therapy to sitagliptin with or without metformin: a 24-week, multicenter, randomized, double-blind, placebo-controlled study. *Diabetes care.* **37,** 740-750 (2014).

42 Bristol-Myers Squibb, AstraZeneca. Dapagliflozin DPPIV Inhibitor add-on Study. National Library of Medicine (US) http://clinicaltrials.gov/show/NCT00984867 (2013). (Date of access: 10 November 2016).

43 Ji, L. *et al.* Dapagliflozin as monotherapy in drug-naive Asian patients with type 2 diabetes mellitus: a randomized, blinded, prospective phase III study. *Clinical therapeutics.* **36,** 84-100 e109 (2014).

44 Ji, L. *et al.* Canagliflozin in Asian patients with type 2 diabetes on metformin alone or metformin in combination with sulphonylurea. *Diabetes, obesity & metabolism.* **17,** 23-31 (2015).

45 Janssen Research & Development, LLC. A Efficacy, Safety, and Tolerability Study of Canagliflozin in Patients With Type 2 Diabetes Mellitus With Inadequate Glycemic Control on Metformin Alone or in Combination With a Sulphonylurea. National Library of Medicine (US) http://clinicaltrials.gov/show/NCT01381900 (2013). (Date of access: 10 November 2016).

46 Kadowaki, T. *et al.* Empagliflozin monotherapy in Japanese patients with type 2 diabetes mellitus: a randomized, 12-week, double-blind, placebo-controlled, phase II trial. *Advances in therapy.* **31,** 621-638 (2014).

47 Eli Lilly and Company, Boehringer Ingelheim. Empagliflozin (BI 10773) Dose Finder Study in Japanese Patients With Type 2 Diabetes Mellitus. National Library of Medicine (US) http://clinicaltrials.gov/show/NCT01368081 (2014). (Date of access: 10 November 2016).

48 Kaku, K. *et al.* Efficacy and safety of dapagliflozin as a monotherapy for type 2 diabetes mellitus in Japanese patients with inadequate glycaemic control: a phase II multicentre, randomized, double-blind, placebo-controlled trial. *Diabetes, obesity & metabolism.* **15,** 432-440 (2013).

49 Bristol-Myers Squibb, AstraZeneca. Trial to Evaluate the Efficacy and Safety of Dapagliflozin in Japanese Type 2 Diabetes Mellitus Patients. National Library of Medicine (US) http://clinicaltrials.gov/show/NCT01368081 (2014). (Date of access: 10 November 2016).

50 Kaku, K. *et al.* Efficacy and safety of monotherapy with the novel sodium/glucose cotransporter-2 inhibitor tofogliflozin in Japanese patients with type 2 diabetes mellitus: a combined Phase 2 and 3 randomized, placebo-controlled, double-blind, parallel-group comparative study. *Cardiovascular diabetology.* **13,** 65 (2014).

51 Kaku, K. *et al.* Efficacy and safety of dapagliflozin monotherapy in Japanese patients with type 2 diabetes inadequately controlled by diet and exercise. *Diabetes, obesity & metabolism.* **16,** 1102-1110 (2014).

52 Kashiwagi, A., Kazuta, K., Yoshida, S. & Nagase, I. Randomized, placebo-controlled, double-blind glycemic control trial of novel sodium-dependent glucose cotransporter 2 inhibitor ipragliflozin in Japanese patients with type 2 diabetes mellitus. *Journal of diabetes investigation.* **5,** 382-391 (2014).

53 Kashiwagi, A. *et al.* A randomized, double-blind, placebo-controlled study on long-term efficacy and safety of ipragliflozin treatment in patients with type 2 diabetes mellitus and renal impairment: results of the long-term ASP1941 safety evaluation in patients with type 2 diabetes with renal impairment (LANTERN) study. *Diabetes, obesity & metabolism.* **17,** 152-160 (2015).

54 Kashiwagi, A. *et al.* Ipragliflozin improves glycemic control in Japanese patients with type 2 diabetes mellitus: the BRIGHTEN study: BRIGHTEN: double-blind randomized study of ipragliflozin to show its efficacy as monotherapy in T2DM patients. *Diabetology International.* **6,** 8-18 (2015).

55 Kashiwagi, A. *et al.* Efficacy and safety of ipragliflozin as an add-on to a sulfonylurea in Japanese patients with inadequately controlled type 2 diabetes: results of the randomized, placebo-controlled, double-blind, phase III EMIT study. *Diabetology International.* **6,** 125-138 (2015).

56 Kashiwagi, A. *et al.* Ipragliflozin in combination with metformin for the treatment of Japanese patients with type 2 diabetes: ILLUMINATE, a randomized, double-blind, placebo-controlled study. *Diabetes, obesity & metabolism.* **17,** 304-308 (2015).

57 Kashiwagi, A. *et al.* Efficacy and safety of ipragliflozin as an add-on to pioglitazone in Japanese patients with inadequately controlled type 2 diabetes: a randomized, double-blind, placebo-controlled study (the SPOTLIGHT study). *Diabetology International.* **6,** 104-116 (2015).

58 Kohan, D. E., Fioretto, P., Tang, W. & List, J. F. Long-term study of patients with type 2 diabetes and moderate renal impairment shows that dapagliflozin reduces weight and blood pressure but does not improve glycemic control. *Kidney international.* **85,** 962-971 (2014).

59 Kovacs, C. S. *et al.* Empagliflozin as Add-on Therapy to Pioglitazone With or Without Metformin in Patients With Type 2 Diabetes Mellitus. *Clinical therapeutics.* **37,** 1773-1788 e1771 (2015).

60 Kovacs, C. S. *et al.* Empagliflozin improves glycaemic and weight control as add-on therapy to pioglitazone or pioglitazone plus metformin in patients with type 2 diabetes: a 24-week, randomized, placebo-controlled trial. *Diabetes, obesity & metabolism.* **16,** 147-158 (2014).

61 Eli Lilly and Company, Boehringer Ingelheim. Efficacy and Safety of Empagliflozin (BI 10773) in Type 2 Diabetes Patients on a Background of Pioglitazone Alone or With Metformin. National Library of Medicine (US) http://clinicaltrials.gov/show/NCT01210001 (2014). (Date of access: 10 November 2016).

62 Lambers Heerspink, H. J., de Zeeuw, D., Wie, L., Leslie, B. & List, J. Dapagliflozin a glucose-regulating drug with diuretic properties in subjects with type 2 diabetes. *Diabetes, obesity & metabolism.* **15,** 853-862 (2013).

63 Lavalle-Gonzalez, F. J. *et al.* Efficacy and safety of canagliflozin compared with placebo and sitagliptin in patients with type 2 diabetes on background metformin monotherapy: a randomised trial. *Diabetologia.* **56,** 2582-2592 (2013).

64 Janssen Research & Development, LLC. The CANTATA-D Trial (CANagliflozin Treatment and Trial Analysis - DPP-4 Inhibitor Comparator Trial). National Library of Medicine (US) http://clinicaltrials.gov/show/NCT01106677 (2013). (Date of access: 10 November 2016).

65 Leiter, L. A. *et al.* Dapagliflozin added to usual care in individuals with type 2 diabetes mellitus with preexisting cardiovascular disease: a 24-week, multicenter, randomized, double-blind, placebo-controlled study with a 28-week extension. *Journal of the American Geriatrics Society.* **62,** 1252-1262 (2014).

66 Bristol-Myers Squibb, AstraZeneca. Efficacy and Safety in Patients With Type 2 Diabetes Mellitus and Cardiovascular Disease. National Library of Medicine (US) http://clinicaltrials.gov/show/NCT01042977 (2013). (Date of access: 10 November 2016).

67 Cefalu, W. T. *et al.* Efficacy and safety of canagliflozin versus glimepiride in patients with type 2 diabetes inadequately controlled with metformin (CANTATA-SU): 52 week results from a randomised, double-blind, phase 3 non-inferiority trial. *Lancet (London, England).* **382,** 941-950 (2013).

68 Leiter, L. A. *et al.* Canagliflozin provides durable glycemic improvements and body weight reduction over 104 weeks versus glimepiride in patients with type 2 diabetes on metformin: a randomized, double-blind, phase 3 study. *Diabetes care.* **38,** 355-364 (2015).

69 Janssen Research & Development, LLC. CANagliflozin Treatment And Trial Analysis-Sulfonylurea (CANTATA-SU) SGLT2 Add-on to Metformin Versus Glimepiride. National Library of Medicine (US) http://clinicaltrials.gov/show/NCT00968812 (2013). (Date of access: 10 November 2016).

70 List, J. F., Woo, V., Morales, E., Tang, W. & Fiedorek, F. T. Sodium-glucose cotransport inhibition with dapagliflozin in type 2 diabetes. *Diabetes care.* **32,** 650-657 (2009).

71 Matthaei, S., Bowering, K., Rohwedder, K., Grohl, A. & Parikh, S. Dapagliflozin improves glycemic control and reduces body weight as add-on therapy to metformin plus sulfonylurea: a 24-week randomized, double-blind clinical trial. *Diabetes care.* **38,** 365-372 (2015).

72 Matthaei, S. *et al.* Durability and tolerability of dapagliflozin over 52 weeks as add-on to metformin and sulphonylurea in type 2 diabetes. *Diabetes, obesity & metabolism.* **17,** 1075-1084 (2015).

73 Bristol-Myers Squibb, AstraZeneca. Evaluation of Safety and Efficacy of Dapagliflozin in Subjects With Type 2 Diabetes Who Have Inadequate Glycaemic Control on Background Combination of Metformin and Sulfonylurea. National Library of Medicine (US) http://clinicaltrials.gov/show/NCT01392677 (2013). (Date of access: 10 November 2016).

74 Mudaliar, S. *et al.* Changes in insulin sensitivity and insulin secretion with the sodium glucose cotransporter 2 inhibitor dapagliflozin. *Diabetes technology & therapeutics.* **16,** 137-144 (2014).

75 Bristol-Myers Squibb, AstraZeneca. Study of Dapagliflozin in Combination With Metformin XR to Initiate the Treatment of Type 2 Diabetes. National Library of Medicine (US) http://clinicaltrials.gov/show/NCT00859898 (2014). (Date of access: 10 November 2016).

76 Haering, H. U. *et al.* Empagliflozin as add-on to metformin plus sulphonylurea in patients with type 2 diabetes. *Diabetes research and clinical practice.* **110,** 82-90 (2015).

77 Haring, H. U. *et al.* Empagliflozin as add-on to metformin in patients with type 2 diabetes: a 24-week, randomized, double-blind, placebo-controlled trial. *Diabetes care.* **37,** 1650-1659 (2014).

78 Haring, H. U. *et al.* Empagliflozin as add-on to metformin plus sulfonylurea in patients with type 2 diabetes: a 24-week, randomized, double-blind, placebo-controlled trial. *Diabetes care.* **36,** 3396-3404 (2013).

79 Merker, L. *et al.* Empagliflozin as add-on to metformin in people with Type 2 diabetes. *Diabetic Medicine.* **32,** 1555-1567 (2015).

80 Eli Lilly and Company, Boehringer Ingelheim. Efficacy and Safety Study With Empagliflozin (BI 10773) vs. Placebo as add-on to Metformin or Metformin Plus Sulfonylurea Over 24 Weeks in Patients With Type 2 Diabetes. National Library of Medicine (US) http://clinicaltrials.gov/show/NCT01159600 (2014). (Date of access: 10 November 2016).

81 Janssen Research & Development, LLC. An Efficacy, Safety, and Tolerability Study of Canagliflozin in the Treatment of Patients With Type 2 Diabetes Mellitus With Inadequate Glycemic Control on Metformin Monotherapy. National Library of Medicine (US) http://clinicaltrials.gov/show/NCT01340664 (2014). (Date of access: 10 November 2016).

82 Eli Lilly and Company, Boehringer Ingelheim. A 16 Weeks Study on Efficacy and Safety of Two Doses of Empagliflozin (BI 10773) (Once Daily Versus Twice Daily) in Patients With Type 2 Diabetes Mellitus and Preexisting Metformin Therapy. National Library of Medicine (US) http://clinicaltrials.gov/show/NCT01649297 (2015). (Date of access: 10 November 2016).

83 Eli Lilly and Company, Boehringer Ingelheim. Safety and Efficacy Study of Empagliflozin and Metformin for 24 Weeks in Treatment Naive Patients With Type 2 Diabetes. National Library of Medicine (US) http://clinicaltrials.gov/show/NCT01719003 (2015). (Date of access: 10 November 2016).

84 Janssen Research & Development, LLC. A Study to Evaluate the Effectiveness, Safety, and Tolerability of Canagliflozin in Combination With Metformin in the Treatment of Patients With Type 2 Diabetes Mellitus With Inadequate Glycemic Control With Diet and Exercise. National Library of Medicine (US) http://clinicaltrials.gov/show/NCT01809327 (2015). (Date of access: 10 November 2016).

85 Fulcher, G. *et al.* Efficacy and safety of canagliflozin when used in conjunction with incretin-mimetic therapy in patients with type 2 diabetes. *Diabetes, Obesity and Metabolism.* **18,** 82-91 (2016).

86 Fulcher, G. *et al.* Efficacy and Safety of Canagliflozin Used in Conjunction with Sulfonylurea in Patients with Type 2 Diabetes Mellitus: A Randomized, Controlled Trial. *Diabetes therapy : research, treatment and education of diabetes and related disorders.* **6,** 289-302 (2015).

87 Neal, B. *et al.* Rationale, design, and baseline characteristics of the Canagliflozin Cardiovascular Assessment Study (CANVAS)--a randomized placebo-controlled trial. *American heart journal.* **166,** 217-223 e211 (2013).

88 Neal, B. *et al.* Efficacy and safety of canagliflozin, an inhibitor of sodium-glucose cotransporter 2, when used in conjunction with insulin therapy in patients with type 2 diabetes. *Diabetes care.* **38,** 403-411 (2015).

89 Ridderstrale, M. *et al.* Comparison of empagliflozin and glimepiride as add-on to metformin in patients with type 2 diabetes: a 104-week randomised, active-

controlled, double-blind, phase 3 trial. *The lancet. Diabetes & endocrinology.* **2,** 691-700 (2014).

90 Eli Lilly and Company, Boehringer Ingelheim. Efficacy and Safety of Empagliflozin (BI 10773) With Metformin in Patients With Type 2 Diabetes. National Library of Medicine (US) http://clinicaltrials.gov/show/NCT01167881 (2014). (Date of access: 10 November 2016).

91 Roden, M. *et al.* Safety, tolerability and effects on cardiometabolic risk factors of empagliflozin monotherapy in drug-naive patients with type 2 diabetes: a double-blind extension of a Phase III randomized controlled trial. *Cardiovascular diabetology.* **14,** 154 (2015).

92 Roden, M. *et al.* Empagliflozin monotherapy with sitagliptin as an active comparator in patients with type 2 diabetes: a randomised, double-blind, placebo-controlled, phase 3 trial. *The lancet. Diabetes & endocrinology.* **1,** 208-219 (2013).

93 Eli Lilly and Company, Boehringer Ingelheim. Efficacy and Safety of Empagliflozin (BI 10773) Versus Placebo and Sitagliptin Over 24 Weeks in Patients With Type 2 Diabetes. National Library of Medicine (US) http://clinicaltrials.gov/show/NCT01177813 (2014). (Date of access: 10 November 2016).

94 Rosenstock, J., Vico, M., Wei, L., Salsali, A. & List, J. F. Effects of dapagliflozin, an SGLT2 inhibitor, on HbA(1c), body weight, and hypoglycemia risk in patients with type 2 diabetes inadequately controlled on pioglitazone monotherapy. *Diabetes care.* **35,** 1473-1478 (2012).

95 Nicolle, L. E., Capuano, G., Ways, K. & Usiskin, K. Effect of canagliflozin, a sodium glucose co-transporter 2 (SGLT2) inhibitor, on bacteriuria and urinary tract infection in subjects with type 2 diabetes enrolled in a 12-week, phase 2 study. *Current medical research and opinion.* **28,** 1167-1171 (2012).

96 Nyirjesy, P., Zhao, Y., Ways, K. & Usiskin, K. Evaluation of vulvovaginal symptoms and Candida colonization in women with type 2 diabetes mellitus treated with canagliflozin, a sodium glucose co-transporter 2 inhibitor. *Current medical research and opinion.* **28,** 1173-1178 (2012).

97 Rosenstock, J. *et al.* Dose-ranging effects of canagliflozin, a sodium-glucose cotransporter 2 inhibitor, as add-on to metformin in subjects with type 2 diabetes. *Diabetes care.* **35,** 1232-1238 (2012).

98 Johnson & Johnson Pharmaceutical Research & Development, L.L.C. An Efficacy, Safety, and Tolerability Study of Canagliflozin (JNJ-28431754) in Patients With Type 2 Diabetes. National Library of Medicine (US) http://clinicaltrials.gov/show/NCT00642278 (2013). (Date of access: 10 November 2016).

99 Rosenstock, J. *et al.* Efficacy and safety of empagliflozin, a sodium glucose cotransporter 2 (SGLT2) inhibitor, as add-on to metformin in type 2 diabetes with mild hyperglycaemia. *Diabetes, obesity & metabolism.* **15,** 1154-1160 (2013).

100 Boehringer Ingelheim. BI 10773 add-on to Metformin in Patients With Type 2 Diabetes. National Library of Medicine (US) http://clinicaltrials.gov/show/NCT00749190 (2014). (Date of access: 10 November 2016).

101 Rosenstock, J. *et al.* Improved glucose control with weight loss, lower insulin doses, and no increased hypoglycemia with empagliflozin added to titrated multiple daily injections of insulin in obese inadequately controlled type 2 diabetes. *Diabetes care.* **37,** 1815-1823 (2014).

102 Eli Lilly and Company, Boehringer Ingelheim. Safety and Efficacy of BI 10773 as add-on to Insulin Regimen in Patients With Type 2 Diabetes Mellitus. National Library of Medicine (US) http://clinicaltrials.gov/show/NCT01306214 (2014). (Date of access: 10 November 2016).

103 Rosenstock, J. *et al.* Greater dose-ranging effects on A1C levels than on glucosuria with LX4211, a dual inhibitor of SGLT1 and SGLT2, in patients with type 2 diabetes on metformin monotherapy. *Diabetes care.* **38,** 431-438 (2015).

104 Rosenstock, J. *et al.* Dual add-on therapy in type 2 diabetes poorly controlled with metformin monotherapy: a randomized double-blind trial of saxagliptin plus dapagliflozin addition versus single addition of saxagliptin or dapagliflozin to metformin. *Diabetes care.* **38,** 376-383 (2015).

105 AstraZeneca. Safety and Efficacy of Combination Saxagliptin & Dapagliflozin Added to Metformin to Treat Subjects With Type 2 Diabetes. National Library of Medicine (US) http://clinicaltrials.gov/show/NCT01606007 (2014). (Date of access: 10 November 2016).

106 Rosenstock, J. *et al.* Impact of empagliflozin added on to basal insulin in type 2 diabetes inadequately controlled on basal insulin: A 78-week randomized, double-blind, placebo-controlled trial. *Diabetes, Obesity and Metabolism.* **17,** 936-948 (2015).

107 Eli Lilly and Company, Boehringer Ingelheim. Efficacy and Safety of BI 10773 in Combination With Insulin in Patients With Type 2 Diabetes. National Library of Medicine (US) http://clinicaltrials.gov/show/NCT01011868 (2014). (Date of access: 10 November 2016).

108 Ross, S. *et al.* Efficacy and safety of empagliflozin twice daily versus once daily in patients with type 2 diabetes inadequately controlled on metformin: a 16-week, randomized, placebo-controlled trial. *Diabetes, obesity & metabolism.* **17,** 699-702 (2015).

109 Schernthaner, G. *et al.* Canagliflozin compared with sitagliptin for patients with type 2 diabetes who do not have adequate glycemic control with metformin plus sulfonylurea: a 52-week randomized trial. *Diabetes care.* **36,** 2508-2515 (2013).

110 Janssen Research & Development, LLC. The CANTATA-D2 Trial (CANagliflozin Treatment And Trial Analysis - DPP-4 Inhibitor Second Comparator Trial). National Library of Medicine (US) http://clinicaltrials.gov/show/NCT01137812 (2013). (Date of access: 10 November 2016).

111 Schumm-Draeger, P. M. *et al.* Twice-daily dapagliflozin co-administered with metformin in type 2 diabetes: a 16-week randomized, placebo-controlled clinical trial. *Diabetes, obesity & metabolism.* **17,** 42-51 (2015).

112 Bristol-Myers Squibb, AstraZeneca. Evaluation of Dapagliflozin Taken Twice-daily. National Library of Medicine (US) http://clinicaltrials.gov/show/NCT01217892 (2013). (Date of access: 10 November 2016).

113 Seino, Y. *et al.* Dose-finding study of luseogliflozin in Japanese patients with type 2 diabetes mellitus: a 12-week, randomized, double-blind, placebo-controlled, phase II study. *Current medical research and opinion.* **30,** 1231-1244 (2014).

114 Seino, Y. *et al.* Efficacy and safety of luseogliflozin as monotherapy in Japanese patients with type 2 diabetes mellitus: a randomized, double-blind, placebo-controlled, phase 3 study. *Current medical research and opinion.* **30,** 1245-1255 (2014).

115 Seino, Y., Sasaki, T., Fukatsu, A., Sakai, S. & Samukawa, Y. Efficacy and safety of luseogliflozin monotherapy in Japanese patients with type 2 diabetes mellitus: a 12-week, randomized, placebo-controlled, phase II study. *Current medical research and opinion.* **30,** 1219-1230 (2014).

116 Stenlof, K. *et al.* Efficacy and safety of canagliflozin monotherapy in subjects with type 2 diabetes mellitus inadequately controlled with diet and exercise. *Diabetes, obesity & metabolism.* **15,** 372-382 (2013).

117 Stenlof, K. *et al.* Long-term efficacy and safety of canagliflozin monotherapy in patients with type 2 diabetes inadequately controlled with diet and exercise: findings from the 52-week CANTATA-M study. *Current medical research and opinion.* **30,** 163-175 (2014).

118 Janssen Research & Development, LLC. The CANTATA-M (CANagliflozin Treatment and Trial Analysis - Monotherapy) Trial. National Library of Medicine (US) http://clinicaltrials.gov/show/NCT01081834 (2013). (Date of access: 10 November 2016).

119 Strojek, K. *et al.* Effect of dapagliflozin in patients with type 2 diabetes who have inadequate glycaemic control with glimepiride: a randomized, 24-week, double-blind, placebo-controlled trial. *Diabetes, obesity & metabolism.* **13,** 928-938 (2011).

120 Strojek, K. *et al.* Dapagliflozin added to glimepiride in patients with type 2 diabetes mellitus sustains glycemic control and weight loss over 48 weeks: a randomized, double-blind, parallel-group, placebo-controlled trial. *Diabetes therapy : research, treatment and education of diabetes and related disorders.* **5,** 267-283 (2014).

121 Bristol-Myers Squibb, AstraZeneca. Efficacy and Safety of Dapagliflozin in Combination With Glimepiride (a Sulphonylurea) in Type 2 Diabetes Patients. National Library of Medicine (US) http://clinicaltrials.gov/show/NCT00680745 (2011). (Date of access: 10 November 2016).

122 Sykes, A. P. *et al.* Randomized trial showing efficacy and safety of twice-daily remogliflozin etabonate for the treatment of type 2 diabetes. *Diabetes, obesity*

& metabolism. **17,** 94-97 (2015).

123 Sykes, A. P. *et al.* Randomized efficacy and safety trial of once-daily remogliflozin etabonate for the treatment of type 2 diabetes. *Diabetes, obesity & metabolism.* **17,** 98-101 (2015).

124 Tikkanen, I. *et al.* Empagliflozin reduces blood pressure in patients with type 2 diabetes and hypertension. *Diabetes care.* **38,** 420-428 (2015).

125 Eli Lilly and Company, Boehringer Ingelheim. 12 Week Efficacy and Safety Study of Empagliflozin (BI 10773) in Hypertensive Patients With Type 2 Diabetes Mellitus. National Library of Medicine (US) http://clinicaltrials.gov/show/NCT01370005 (2014). (Date of access: 10 November 2016).

126 Weber, M. A. *et al.* Blood pressure and glycaemic effects of dapagliflozin versus placebo in patients with type 2 diabetes on combination antihypertensive therapy: a randomised, double-blind, placebo-controlled, phase 3 study. *The lancet. Diabetes & endocrinology.* (2015).

127 Bristol-Myers Squibb, AstraZeneca. A Study of BMS-512148 (Dapagliflozin) in Patients With Type 2 Diabetes With Inadequately Controlled Hypertension on an ACEI or ARB and an Additional Antihypertensive Medication. National Library of Medicine (US) http://clinicaltrials.gov/show/NCT01195662 (2014). (Date of access: 10 November 2016).

128 Weber, M. A. *et al.* Effects of dapagliflozin on blood pressure in hypertensive diabetic patients on renin-angiotensin system blockade. *Blood pressure.* **25,** 93-103 (2016).

129 AstraZeneca, Bristol-Myers Squibb. A Study of BMS-512148 (Dapagliflozin) in Patients With Type 2 Diabetes and Inadequately Controlled Hypertension on an Angiotensin-Converting Enzyme Inhibitor or Angiotensin Receptor Blocker. National Library of Medicine (US) http://clinicaltrials.gov/show/NCT01137474 (2014). (Date of access: 10 November 2016).

130 Wilding, J. P. *et al.* A study of dapagliflozin in patients with type 2 diabetes receiving high doses of insulin plus insulin sensitizers: applicability of a novel insulin-independent treatment. *Diabetes care.* **32,** 1656-1662 (2009).

131 Wilding, J. P. *et al.* Efficacy and safety of ipragliflozin in patients with type 2 diabetes inadequately controlled on metformin: a dose-finding study. *Diabetes, obesity & metabolism.* **15,** 403-409 (2013).

132 Wilding, J. P. *et al.* Efficacy and safety of canagliflozin in patients with type 2 diabetes mellitus inadequately controlled with metformin and sulphonylurea: a randomised trial. *International journal of clinical practice.* **67,** 1267-1282 (2013).

133 Janssen Research & Development, LLC. The CANTATA-MSU Trial (CANagliflozin Treatment And Trial Analysis - Metformin and SUlphonylurea). National Library of Medicine (US) http://clinicaltrials.gov/show/NCT01106625 (2013). (Date of access: 10 November 2016).

134 Wilding, J. P., Woo, V., Rohwedder, K., Sugg, J. & Parikh, S. Dapagliflozin in patients with type 2 diabetes receiving high doses of insulin: efficacy and safety over 2 years. *Diabetes, obesity & metabolism.* **16,** 124-136 (2014).

135 Wilding, J. P. *et al.* Long-term efficacy of dapagliflozin in patients with type 2 diabetes mellitus receiving high doses of insulin: a randomized trial. *Annals of internal medicine.* **156,** 405-415 (2012).

136 Bristol-Myers Squibb, AstraZeneca. Efficacy and Safety of Dapagliflozin, Added to Therapy of Patients With Type 2 Diabetes With Inadequate Glycemic Control on Insulin. National Library of Medicine (US) http://clinicaltrials.gov/show/NCT00673231 (2013). (Date of access: 10 November 2016).

137 Ws, W. J. *et al.* Switching from sulphonylurea to an SGLT2 inhibitor in the fasting month of Ramadan is associated with a reduction in hypoglycaemia. *Diabetes, obesity & metabolism.* (2016).

138 Yale, J. F. *et al.* Efficacy and safety of canagliflozin over 52 weeks in patients with type 2 diabetes mellitus and chronic kidney disease. *Diabetes, obesity & metabolism.* **16,** 1016-1027 (2014).

139 Yale, J. F. *et al.* Efficacy and safety of canagliflozin in subjects with type 2 diabetes and chronic kidney disease. *Diabetes, obesity & metabolism.* **15,** 463-473 (2013).

140 Janssen Research & Development, LLC. An Efficacy, Safety, and Tolerability Study of Canagliflozin in Patients With Type 2 Diabetes Mellitus Who Have Moderate Renal Impairment. National Library of Medicine (US) http://clinicaltrials.gov/show/NCT01064414 (2013). (Date of access: 10 November 2016).

141 Yang, W. *et al.* Efficacy and safety of dapagliflozin in Asian patients with type 2 diabetes after metformin failure: A randomized controlled trial. *Journal of diabetes.* (2015).

142 Zinman, B. *et al.* Rationale, design, and baseline characteristics of a randomized, placebo-controlled cardiovascular outcome trial of empagliflozin (EMPA-REG OUTCOME). *Cardiovascular diabetology.* **13,** 102 (2014).

143 Zinman, B. *et al.* Empagliflozin, Cardiovascular Outcomes, and Mortality in Type 2 Diabetes. *The New England journal of medicine.* **373,** 2117-2128 (2015).

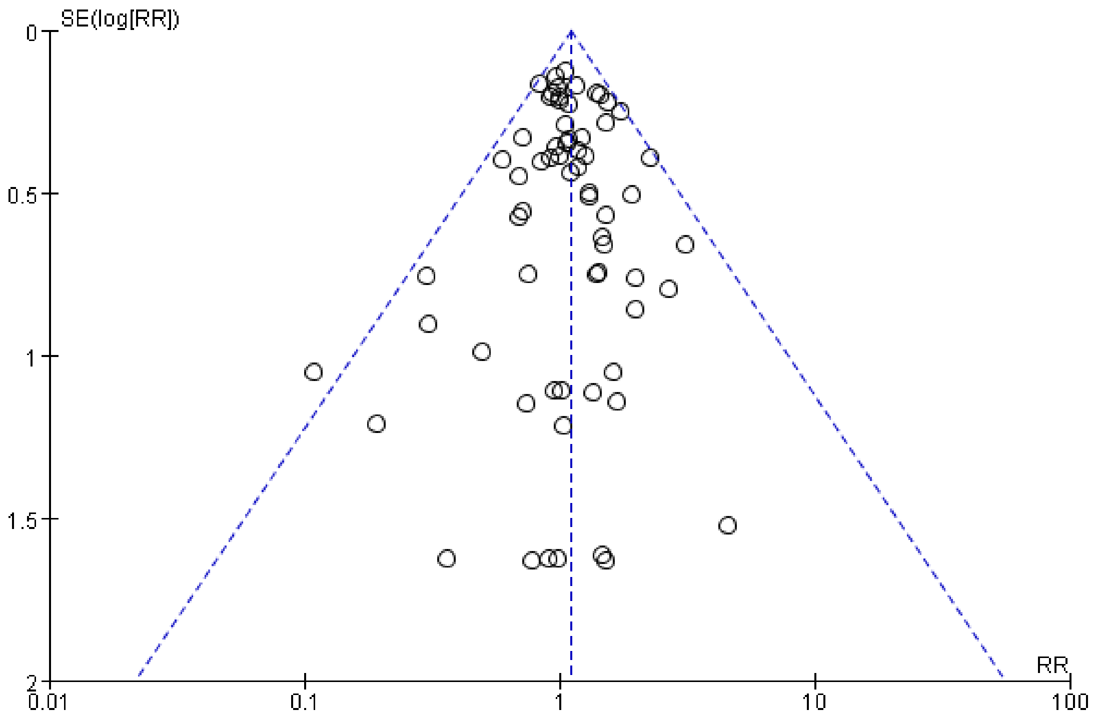

Figure 1S. Funnel plot of urinary tract infections among patients receiving SGLT2 inhibitors versus control in randomized controlled trials

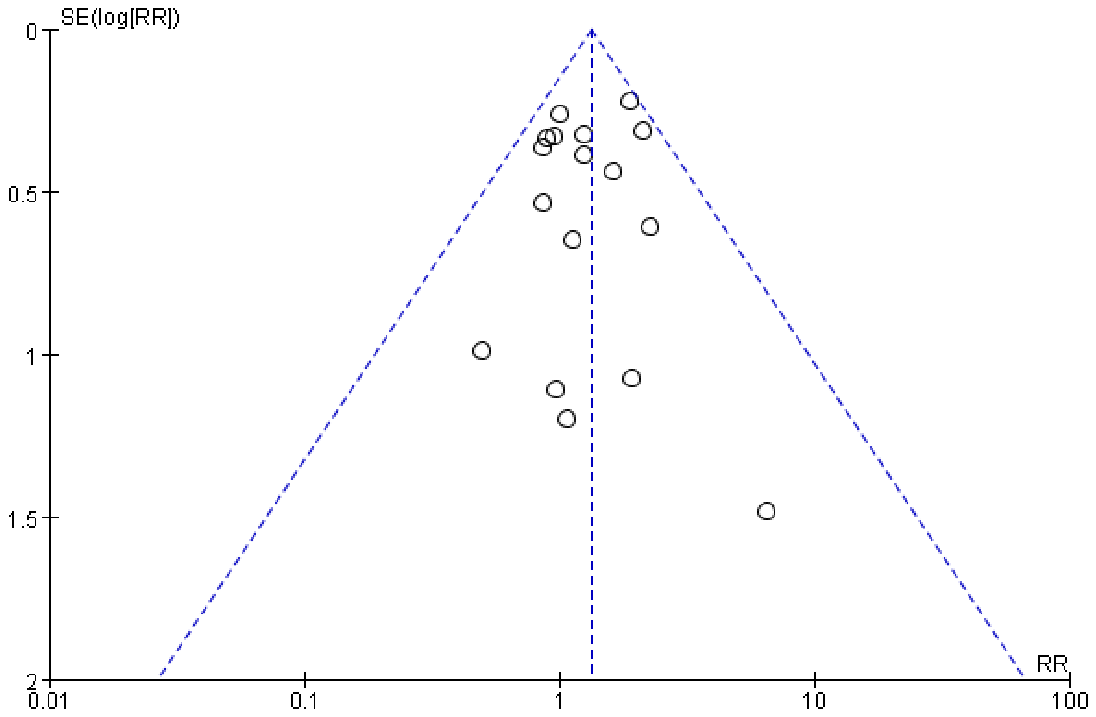

Figure 2S. Funnel plot of events suggestive of UTIs among patients receiving SGLT2 inhibitors versus control in randomized controlled trials

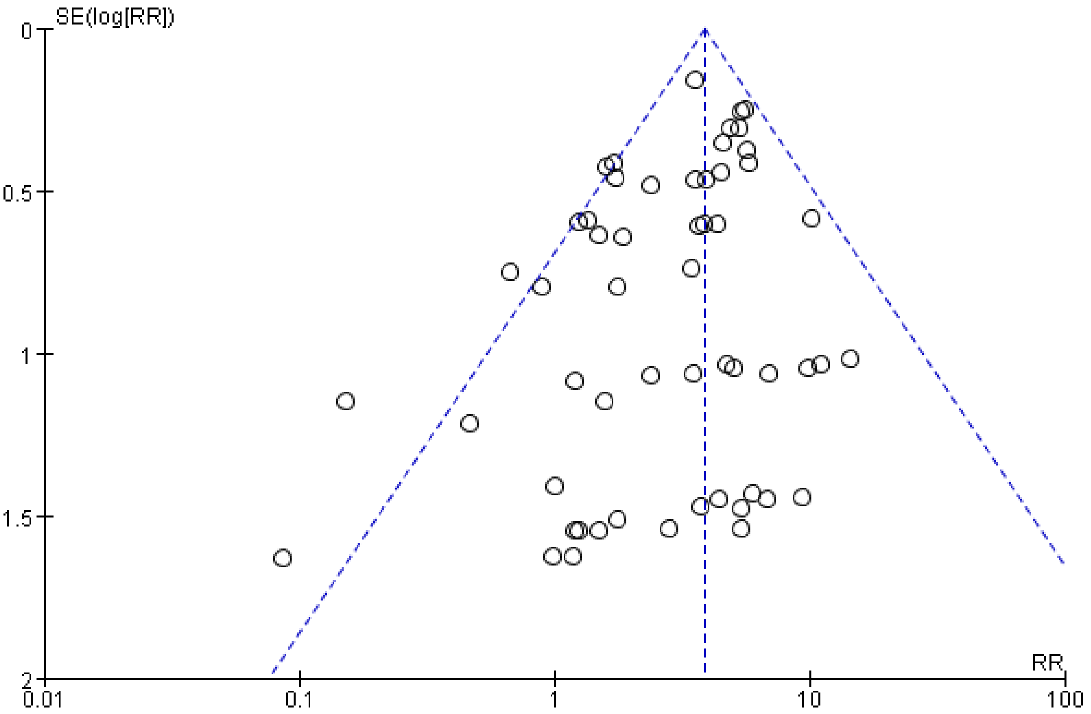

Figure 3S. Funnel plot of genital infections among patients receiving SGLT2 inhibitors versus control in randomized controlled trials

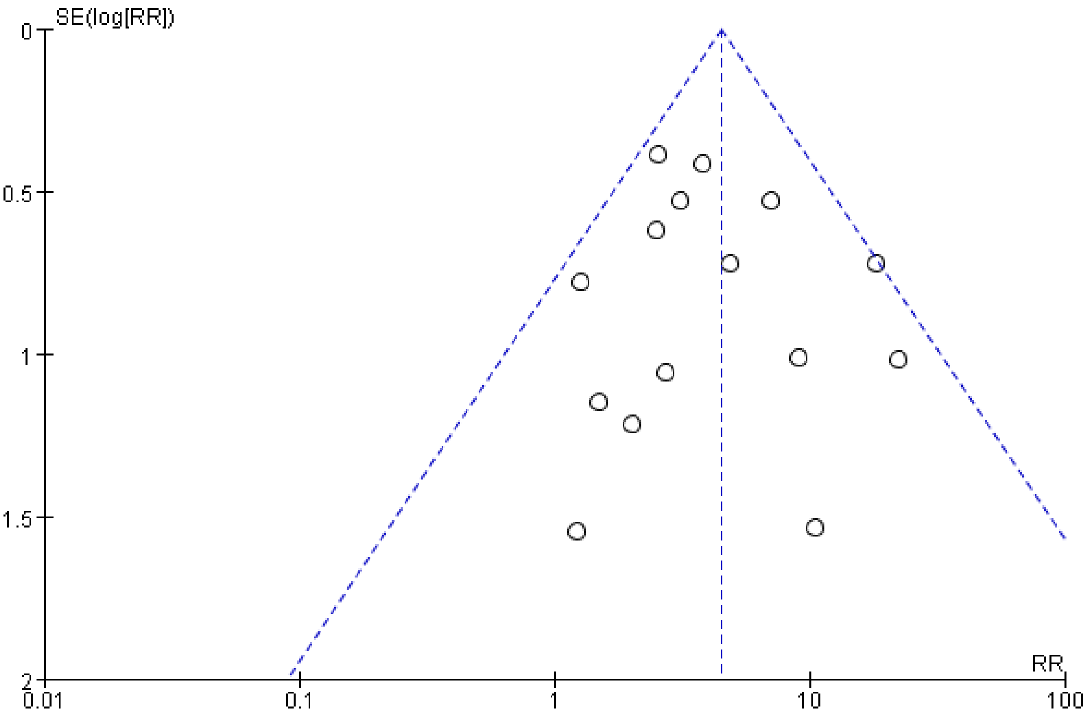

Figure 4S. Funnel plot of events suggestive of genital infections among patients receiving SGLT2 inhibitors versus control in randomized controlled trials

	SGLT2 inhibitors		Control			Risk Ratio
Study or Subgroup	Events	Total	Events	Total	Weight	M-H, Random, 95% CI
**1.1.1 Canagliflozin**						
Bode 2015	74	477	24	237	2.2%	1.53 [0.99, 2.36]
Forst 2014	15	227	9	115	0.6%	0.84 [0.38, 1.87]
Inagaki 2013	0	307	0	75		Not estimable
Inagaki 2014	2	179	1	93	0.1%	1.04 [0.10, 11.31]
Ji 2015	13	450	11	226	0.7%	0.59 [0.27, 1.30]
Lavalle-Gonzale 2013	47	735	35	549	2.3%	1.00 [0.66, 1.53]
Leiter 2015	93	968	33	482	2.8%	1.40 [0.96, 2.06]
NCT01340664 2014	1	186	0	93	0.0%	1.51 [0.06, 36.66]
NCT01809327 2015	16	949	3	237	0.3%	1.33 [0.39, 4.53]
Neal 2015	102	1743	44	867	3.5%	1.15 [0.82, 1.63]
Rosenstock 2012B	16	321	5	130	0.4%	1.30 [0.48, 3.46]
Schernthaner 2013	15	377	21	378	1.0%	0.72 [0.37, 1.37]
Stenlöf 2014	30	392	12	192	1.0%	1.22 [0.64, 2.34]
Wilding 2013B	26	313	12	156	1.0%	1.08 [0.56, 2.08]
Yale 2014	18	179	9	90	0.7%	1.01 [0.47, 2.15]
**Subtotal (95% CI)**		**7803**		**3920**	**16.5%**	**1.13 [0.97, 1.33]**
Total events	468		219			
Heterogeneity: Tau² = 0.00; Chi² = 8.79, df = 13 (P = 0.79); I² = 0%						
Test for overall effect: Z = 1.57 (P = 0.12)						
**1.1.2 Dapagliflozin**						
Bailey 2013	30	409	8	137	0.7%	1.26 [0.59, 2.67]
Bolinder 2014	3	91	4	91	0.2%	0.75 [0.17, 3.26]
Cefalu 2015	23	460	22	462	1.3%	1.05 [0.59, 1.86]
Del Prato 2015	55	406	38	408	2.7%	1.45 [0.98, 2.15]
Ji 2014	12	261	4	132	0.3%	1.52 [0.50, 4.61]
Leiter 2014	40	482	23	483	1.7%	1.74 [1.06, 2.87]
List 2009	21	279	7	110	0.6%	1.18 [0.52, 2.70]
Matthaei 2015	11	109	12	109	0.7%	0.92 [0.42, 1.99]
Mudaliar 2014	2	23	0	21	0.0%	4.58 [0.23, 90.30]
NCT00859898 2014	6	211	4	208	0.3%	1.48 [0.42, 5.16]
Rosenstock 2015B	1	179	9	176	0.1%	0.11 [0.01, 0.85]
Strojek 2014	15	450	7	146	0.5%	0.70 [0.29, 1.67]
Weber 2015	4	225	2	224	0.1%	1.99 [0.37, 10.76]
Weber 2016	9	302	3	311	0.2%	3.09 [0.84, 11.30]
Wilding 2009	1	48	0	23	0.0%	1.47 [0.06, 34.74]
Wilding 2014	49	610	7	197	0.7%	2.26 [1.04, 4.91]
Ws 2016	6	58	2	52	0.2%	2.69 [0.57, 12.75]
Yang 2015	16	299	7	145	0.5%	1.11 [0.47, 2.63]
**Subtotal (95% CI)**		**4902**		**3435**	**10.9%**	**1.34 [1.11, 1.63]**
Total events	304		159			
Heterogeneity: Tau² = 0.00; Chi² = 16.69, df = 17 (P = 0.48); I² = 0%						
Test for overall effect: Z = 2.98 (P = 0.003)						
**1.1.3 Empagliflozin**						
Araki 2015	12	273	2	63	0.2%	1.38 [0.32, 6.03]
Barnett 2014	61	419	47	319	3.3%	0.99 [0.70, 1.40]
DeFronzo 2015	65	541	34	263	2.7%	0.93 [0.63, 1.37]
Ferrannini 2013A	47	547	9	112	0.9%	1.07 [0.54, 2.12]
Ferrannini 2013B	4	244	1	82	0.1%	1.34 [0.15, 11.86]
Kadowaki 2014	3	438	1	109	0.1%	0.75 [0.08, 7.11]
Kovacs 2015	74	333	44	165	3.9%	0.83 [0.60, 1.15]
NCT01159600-2014	126	872	64	431	5.3%	0.97 [0.74, 1.28]
NCT01649297 2015	43	876	4	107	0.4%	1.31 [0.48, 3.59]
NCT01719003 2015	81	1019	26	341	2.3%	1.04 [0.68, 1.59]
Ridderstrale 2014	105	765	102	780	6.4%	1.05 [0.81, 1.35]
Roden 2015	41	447	45	452	2.5%	0.92 [0.62, 1.38]
Rosenstock 2013	14	353	2	71	0.2%	1.41 [0.33, 6.06]
Rosenstock 2014	58	375	29	188	2.4%	1.00 [0.67, 1.51]
Rosenstock 2015C	43	324	15	170	1.3%	1.50 [0.86, 2.63]
Ross 2015	63	876	4	107	0.4%	1.92 [0.71, 5.18]
Tikkanen 2015	24	552	10	272	0.8%	1.18 [0.57, 2.44]
Zinman 2015	842	4687	423	2333	36.7%	0.99 [0.89, 1.10]
**Subtotal (95% CI)**		**13941**		**6365**	**70.0%**	**1.00 [0.93, 1.08]**
Total events	1706		862			
Heterogeneity: Tau² = 0.00; Chi² = 6.59, df = 17 (P = 0.99); I² = 0%						
Test for overall effect: Z = 0.00 (P = 1.00)						
**1.1.5 Ipragliflozin**						
Fonseca 2013	21	273	11	138	0.8%	0.97 [0.48, 1.94]
Kashiwagi 2014	4	291	1	69	0.1%	0.95 [0.11, 8.35]
Kashiwagi 2015A	1	119	2	46	0.1%	0.19 [0.02, 2.08]
Kashiwagi 2015B	0	62	1	67	0.0%	0.36 [0.01, 8.67]
Kashiwagi 2015C	2	166	3	76	0.1%	0.31 [0.05, 1.79]
Kashiwagi 2015D	2	112	2	56	0.1%	0.50 [0.07, 3.46]
Kashiwagi 2015E	3	97	1	54	0.1%	1.67 [0.18, 15.67]
Wilding 2013A	12	276	4	66	0.3%	0.72 [0.24, 2.15]
**Subtotal (95% CI)**		**1396**		**572**	**1.7%**	**0.75 [0.46, 1.22]**
Total events	45		25			
Heterogeneity: Tau² = 0.00; Chi² = 3.66, df = 7 (P = 0.82); I² = 0%						
Test for overall effect: Z = 1.17 (P = 0.24)						
**1.1.6 Other agents**						
Amin 2015	7	219	5	109	0.3%	0.70 [0.23, 2.15]
Ikeda 2015	8	328	1	66	0.1%	1.61 [0.20, 12.66]
Kaku 2014A	1	174	0	56	0.0%	0.98 [0.04, 23.65]
Seino 2014A	1	223	0	57	0.0%	0.78 [0.03, 18.82]
Seino 2014B	0	79	0	79		Not estimable
Seino 2014C	1	182	0	54	0.0%	0.90 [0.04, 21.82]
Sykes 2015A	3	238	4	96	0.2%	0.30 [0.07, 1.33]
Sykes 2015B	10	179	2	71	0.2%	1.98 [0.45, 8.83]
**Subtotal (95% CI)**		**1622**		**588**	**0.9%**	**0.82 [0.42, 1.60]**
Total events	31		12			
Heterogeneity: Tau² = 0.00; Chi² = 3.64, df = 6 (P = 0.73); I² = 0%						
Test for overall effect: Z = 0.59 (P = 0.55)						
**Total (95% CI)**		**29664**		**14880**	**100.0%**	**1.05 [0.98, 1.12]**
Total events	2554		1277			
Heterogeneity: Tau² = 0.00; Chi² = 50.47, df = 64 (P = 0.89); I² = 0%						
Test for overall effect: Z = 1.42 (P = 0.16)						
Test for subgroup differences: Chi² = 11.07, df = 4 (P = 0.03), I² = 63.9%						

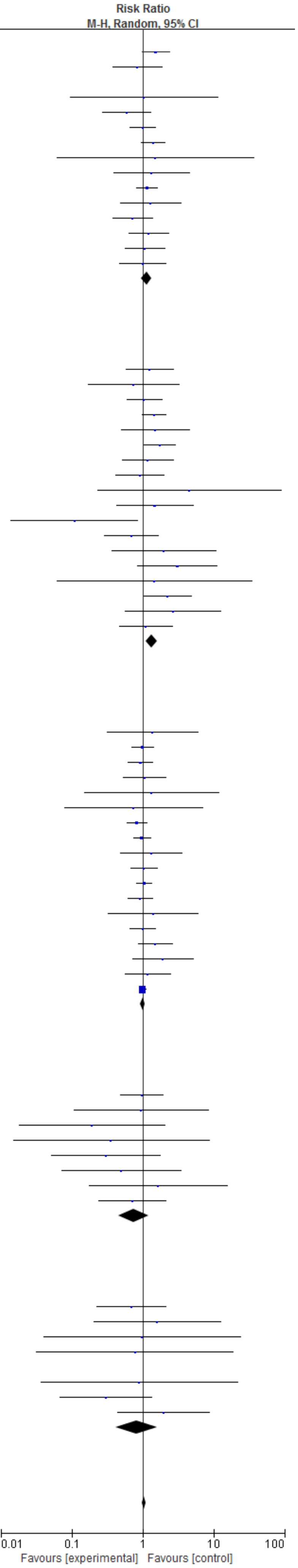

Figure 5S. Subgroup analysis of UTIs by individual SGLT2 inhibitors in randomized controlled trials

Study or Subgroup	SGLT2 inhibitors Events	SGLT2 inhibitors Total	Control Events	Control Total	Weight	Risk Ratio M-H, Random, 95% CI
**1.2.1 SGLT-2 vs. placebo**						
Amin 2015	7	219	4	54	0.3%	0.43 [0.13, 1.42]
Bailey 2013	30	409	8	137	0.7%	1.26 [0.59, 2.67]
Barnett 2014	61	419	47	319	3.1%	0.99 [0.70, 1.40]
Bode 2015	74	477	24	237	2.1%	1.53 [0.99, 2.36]
Bolinder 2014	3	91	4	91	0.2%	0.75 [0.17, 3.26]
Cefalu 2015	23	460	22	462	1.2%	1.05 [0.59, 1.86]
DeFronzo 2015	65	541	34	263	2.6%	0.93 [0.63, 1.37]
Ferrannini 2013B	4	244	1	82	0.1%	1.34 [0.15, 11.86]
Fonseca 2013	21	273	6	69	0.5%	0.88 [0.37, 2.11]
Ikeda 2015	8	328	1	66	0.1%	1.61 [0.20, 12.66]
Inagaki 2013	0	307	0	75		Not estimable
Inagaki 2014	2	179	1	93	0.1%	1.04 [0.10, 11.31]
Ji 2014	12	261	4	132	0.3%	1.52 [0.50, 4.61]
Ji 2015	13	450	11	226	0.6%	0.59 [0.27, 1.30]
Kadowaki 2014	3	438	1	109	0.1%	0.75 [0.08, 7.11]
Kaku 2014A	1	174	0	56	0.0%	0.98 [0.04, 23.65]
Kashiwagi 2014	4	291	1	69	0.1%	0.95 [0.11, 8.35]
Kashiwagi 2015A	1	119	2	46	0.1%	0.19 [0.02, 2.08]
Kashiwagi 2015B	0	62	1	67	0.0%	0.36 [0.01, 8.67]
Kashiwagi 2015C	2	166	3	76	0.1%	0.31 [0.05, 1.79]
Kashiwagi 2015D	2	112	2	56	0.1%	0.50 [0.07, 3.46]
Kashiwagi 2015E	3	97	1	54	0.1%	1.67 [0.18, 15.67]
Kovacs 2015	74	333	44	165	3.7%	0.83 [0.60, 1.15]
Leiter 2014	40	482	23	483	1.6%	1.74 [1.06, 2.87]
List 2009	21	279	3	54	0.3%	1.35 [0.42, 4.38]
Matthaei 2015	11	109	12	109	0.6%	0.92 [0.42, 1.99]
Mudaliar 2014	2	23	0	21	0.0%	4.58 [0.23, 90.30]
NCT00859898 2014	6	211	4	208	0.2%	1.48 [0.42, 5.16]
NCT01159600-2014	126	872	64	431	5.0%	0.97 [0.74, 1.28]
NCT01340664 2014	1	186	0	93	0.0%	1.51 [0.06, 36.66]
NCT01649297 2015	43	876	4	107	0.4%	1.31 [0.48, 3.59]
NCT01719003 2015	56	680	26	341	1.9%	1.08 [0.69, 1.69]
NCT01809327 2015	9	474	3	237	0.2%	1.50 [0.41, 5.49]
Neal 2015	102	1743	44	867	3.3%	1.15 [0.82, 1.63]
Roden 2015	41	447	25	229	1.7%	0.84 [0.52, 1.35]
Rosenstock 2012B	16	321	4	65	0.3%	0.81 [0.28, 2.34]
Rosenstock 2013	14	353	2	71	0.2%	1.41 [0.33, 6.06]
Rosenstock 2014	58	375	29	188	2.3%	1.00 [0.67, 1.51]
Rosenstock 2015A	4	236	1	60	0.1%	1.02 [0.12, 8.93]
Rosenstock 2015B	1	179	9	176	0.1%	0.11 [0.01, 0.85]
Rosenstock 2015C	43	324	15	170	1.2%	1.50 [0.86, 2.63]
Ross 2015	63	876	4	107	0.4%	1.92 [0.71, 5.18]
Seino 2014A	1	223	0	57	0.0%	0.78 [0.03, 18.82]
Seino 2014B	0	79	0	79		Not estimable
Seino 2014C	1	182	0	54	0.0%	0.90 [0.04, 21.82]
Strojek 2014	15	450	7	146	0.5%	0.70 [0.29, 1.67]
Sykes 2015A	3	238	0	48	0.0%	1.44 [0.08, 27.34]
Sykes 2015B	10	179	0	36	0.0%	4.32 [0.26, 72.05]
Tikkanen 2015	24	552	10	272	0.7%	1.18 [0.57, 2.44]
Weber 2015	4	225	2	224	0.1%	1.99 [0.37, 10.76]
Weber 2016	9	302	3	311	0.2%	3.09 [0.84, 11.30]
Wilding 2009	1	48	0	23	0.0%	1.47 [0.06, 34.74]
Wilding 2013A	12	276	4	66	0.3%	0.72 [0.24, 2.15]
Wilding 2013B	26	313	12	156	0.9%	1.08 [0.56, 2.08]
Wilding 2014	49	610	7	197	0.6%	2.26 [1.04, 4.91]
Yale 2014	18	179	9	90	0.7%	1.01 [0.47, 2.15]
Yang 2015	16	299	7	145	0.5%	1.11 [0.47, 2.63]
Zinman 2015	842	4687	423	2333	34.5%	0.99 [0.89, 1.10]
**Subtotal (95% CI)**		**24338**		**11258**	**75.3%**	**1.03 [0.96, 1.10]**
Total events	2101		978			
Heterogeneity: Tau² = 0.00; Chi² = 41.86, df = 55 (P = 0.90); I² = 0%						
Test for overall effect: Z = 0.72 (P = 0.47)						
**1.2.2 SGLT-2 vs. active drugs**						
Amin 2015	7	219	1	55	0.1%	1.76 [0.22, 13.99]
Araki 2015	12	273	2	63	0.2%	1.38 [0.32, 6.03]
DeFronzo 2015	71	547	34	263	2.6%	1.00 [0.69, 1.47]
Del Prato 2015	55	406	38	408	2.5%	1.45 [0.98, 2.15]
Ferrannini 2013A	47	547	9	112	0.8%	1.07 [0.54, 2.12]
Fonseca 2013	21	273	5	69	0.4%	1.06 [0.42, 2.71]
Forst 2014	15	227	9	115	0.6%	0.84 [0.38, 1.87]
Lavalle-Gonzale 2013	47	735	35	549	2.1%	1.00 [0.66, 1.53]
Leiter 2015	93	968	33	482	2.6%	1.40 [0.96, 2.06]
List 2009	21	279	4	56	0.4%	1.05 [0.38, 2.95]
NCT00859898 2014	17	219	4	208	0.3%	4.04 [1.38, 11.80]
NCT01719003 2015	25	339	26	341	1.4%	0.97 [0.57, 1.64]
NCT01809327 2015	7	475	3	237	0.2%	1.16 [0.30, 4.46]
Ridderstrale 2014	105	765	102	780	6.0%	1.05 [0.81, 1.35]
Roden 2015	41	447	20	223	1.5%	1.02 [0.61, 1.70]
Rosenstock 2012B	16	321	1	65	0.1%	3.24 [0.44, 24.00]
Rosenstock 2015B	7	179	9	176	0.4%	0.76 [0.29, 2.01]
Schernthaner 2013	15	377	21	378	0.9%	0.72 [0.37, 1.37]
Stenlöf 2014	30	392	12	192	0.9%	1.22 [0.64, 2.34]
Sykes 2015A	3	238	4	48	0.2%	0.15 [0.03, 0.65]
Sykes 2015B	10	179	2	35	0.2%	0.98 [0.22, 4.27]
Ws 2016	6	58	2	52	0.2%	2.69 [0.57, 12.75]
**Subtotal (95% CI)**		**8463**		**4907**	**24.7%**	**1.10 [0.96, 1.26]**
Total events	671		376			
Heterogeneity: Tau² = 0.01; Chi² = 22.62, df = 21 (P = 0.36); I² = 7%						
Test for overall effect: Z = 1.42 (P = 0.16)						
**Total (95% CI)**		**32801**		**16165**	**100.0%**	**1.05 [0.98, 1.11]**
Total events	2772		1354			
Heterogeneity: Tau² = 0.00; Chi² = 65.47, df = 77 (P = 0.82); I² = 0%						
Test for overall effect: Z = 1.39 (P = 0.16)						
Test for subgroup differences: Chi² = 0.85, df = 1 (P = 0.36), I² = 0%						

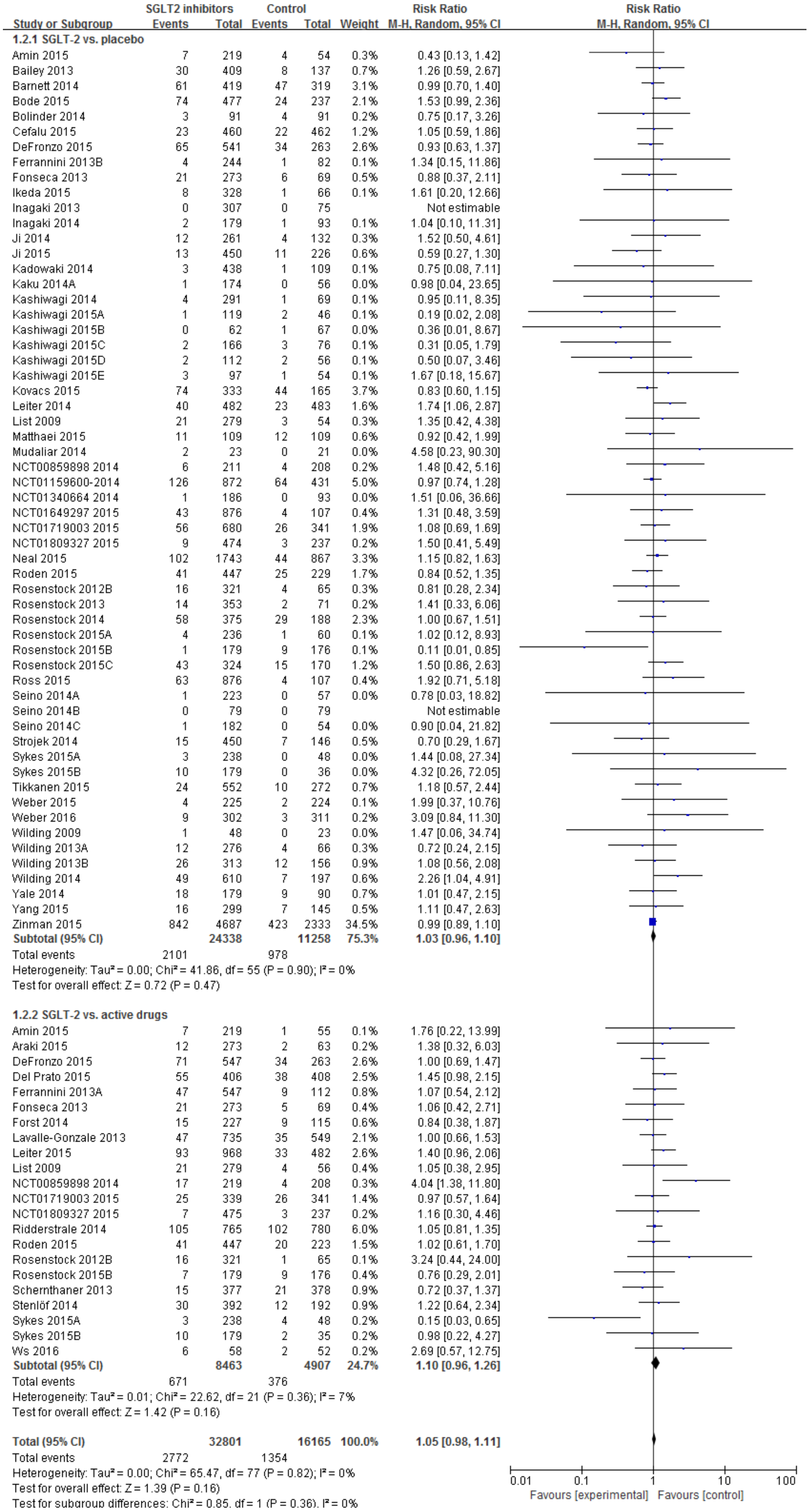

Figure 6S. Subgroup analysis of UTIs by type of control in randomized controlled trials

Study or Subgroup	SGLT2 inhibitors Events	SGLT2 inhibitors Total	Control Events	Control Total	Weight	Risk Ratio M-H, Random, 95% CI
**1.3.1 Follow up 26 weeks or shorter**						
Amin 2015	7	219	5	109	0.3%	0.70 [0.23, 2.15]
Ferrannini 2013B	4	244	1	82	0.1%	1.34 [0.15, 11.86]
Fonseca 2013	21	273	11	138	0.8%	0.97 [0.48, 1.94]
Ikeda 2015	8	328	1	66	0.1%	1.61 [0.20, 12.66]
Inagaki 2013	0	307	0	75		Not estimable
Inagaki 2014	2	179	1	93	0.1%	1.04 [0.10, 11.31]
Ji 2014	12	261	4	132	0.3%	1.52 [0.50, 4.61]
Ji 2015	13	450	11	226	0.7%	0.59 [0.27, 1.30]
Kadowaki 2014	3	438	1	109	0.1%	0.75 [0.08, 7.11]
Kaku 2014A	1	174	0	56	0.0%	0.98 [0.04, 23.65]
Kashiwagi 2014	4	291	1	69	0.1%	0.95 [0.11, 8.35]
Kashiwagi 2015B	0	62	1	67	0.0%	0.36 [0.01, 8.67]
Kashiwagi 2015C	2	166	3	76	0.1%	0.31 [0.05, 1.79]
Kashiwagi 2015D	2	112	2	56	0.1%	0.50 [0.07, 3.46]
Kashiwagi 2015E	3	97	1	54	0.1%	1.67 [0.18, 15.67]
List 2009	21	279	7	110	0.6%	1.18 [0.52, 2.70]
Mudaliar 2014	2	23	0	21	0.0%	4.58 [0.23, 90.30]
NCT00859898 2014	6	211	4	208	0.3%	1.48 [0.42, 5.16]
NCT01340664 2014	1	186	0	93	0.0%	1.51 [0.06, 36.66]
NCT01649297 2015	43	876	4	107	0.4%	1.31 [0.48, 3.59]
NCT01719003 2015	81	1019	26	341	2.3%	1.04 [0.68, 1.59]
NCT01809327 2015	16	949	3	237	0.3%	1.33 [0.39, 4.53]
Rosenstock 2012B	16	321	5	130	0.4%	1.30 [0.48, 3.46]
Rosenstock 2013	14	353	2	71	0.2%	1.41 [0.33, 6.06]
Rosenstock 2015A	4	236	1	60	0.1%	1.02 [0.12, 8.93]
Rosenstock 2015B	1	179	9	176	0.1%	0.11 [0.01, 0.85]
Ross 2015	63	876	4	107	0.4%	1.92 [0.71, 5.18]
Seino 2014A	1	223	0	57	0.0%	0.78 [0.03, 18.82]
Seino 2014B	0	79	0	79		Not estimable
Seino 2014C	1	182	0	54	0.0%	0.90 [0.04, 21.82]
Sykes 2015A	3	238	4	96	0.2%	0.30 [0.07, 1.33]
Sykes 2015B	10	179	2	71	0.2%	1.98 [0.45, 8.83]
Tikkanen 2015	24	552	10	272	0.8%	1.18 [0.57, 2.44]
Weber 2015	4	225	2	224	0.1%	1.99 [0.37, 10.76]
Weber 2016	9	302	3	311	0.2%	3.09 [0.84, 11.30]
Wilding 2009	1	48	0	23	0.0%	1.47 [0.06, 34.74]
Wilding 2013A	12	276	4	66	0.3%	0.72 [0.24, 2.15]
Ws 2016	6	58	2	52	0.2%	2.69 [0.57, 12.75]
Yang 2015	16	299	7	145	0.5%	1.11 [0.47, 2.63]
**Subtotal (95% CI)**		**11770**		**4519**	**10.8%**	**1.08 [0.89, 1.31]**
Total events	437		142			
Heterogeneity: $Tau^2 = 0.00$; $Chi^2 = 23.12$, df = 36 (P = 0.95); $I^2 = 0\%$						
Test for overall effect: Z = 0.73 (P = 0.46)						
**1.3.2 Follow up 26-52 weeks**						
Araki 2015	12	273	2	63	0.2%	1.38 [0.32, 6.03]
Barnett 2014	61	419	47	319	3.3%	0.99 [0.70, 1.40]
Cefalu 2015	23	460	22	462	1.3%	1.05 [0.59, 1.86]
DeFronzo 2015	65	541	34	263	2.7%	0.93 [0.63, 1.37]
Forst 2014	15	227	9	115	0.6%	0.84 [0.38, 1.87]
Kashiwagi 2015A	1	119	2	46	0.1%	0.19 [0.02, 2.08]
Lavalle-Gonzale 2013	47	735	35	549	2.3%	1.00 [0.66, 1.53]
Leiter 2014	40	482	23	483	1.7%	1.74 [1.06, 2.87]
Matthaei 2015	11	109	12	109	0.7%	0.92 [0.42, 1.99]
Neal 2015	102	1743	44	867	3.5%	1.15 [0.82, 1.63]
Rosenstock 2014	58	375	29	188	2.4%	1.00 [0.67, 1.51]
Schernthaner 2013	15	377	21	378	1.0%	0.72 [0.37, 1.37]
Stenlöf 2014	30	392	12	192	1.0%	1.22 [0.64, 2.34]
Strojek 2014	15	450	7	146	0.5%	0.70 [0.29, 1.67]
Wilding 2013B	26	313	12	156	1.0%	1.08 [0.56, 2.08]
Yale 2014	18	179	9	90	0.7%	1.01 [0.47, 2.15]
**Subtotal (95% CI)**		**7194**		**4426**	**22.9%**	**1.03 [0.91, 1.18]**
Total events	539		320			
Heterogeneity: $Tau^2 = 0.00$; $Chi^2 = 9.74$, df = 15 (P = 0.84); $I^2 = 0\%$						
Test for overall effect: Z = 0.50 (P = 0.62)						
**1.3.3 Follow up over 52 weeks**						
Bailey 2013	30	409	8	137	0.7%	1.26 [0.59, 2.67]
Bode 2015	74	477	24	237	2.2%	1.53 [0.99, 2.36]
Bolinder 2014	3	91	4	91	0.2%	0.75 [0.17, 3.26]
Del Prato 2015	55	406	38	408	2.7%	1.45 [0.98, 2.15]
Ferrannini 2013A	47	547	9	112	0.9%	1.07 [0.54, 2.12]
Kovacs 2015	74	333	44	165	3.9%	0.83 [0.60, 1.15]
Leiter 2015	93	968	33	482	2.8%	1.40 [0.96, 2.06]
NCT01159600-2014	126	872	64	431	5.3%	0.97 [0.74, 1.28]
Ridderstrale 2014	105	765	102	780	6.4%	1.05 [0.81, 1.35]
Roden 2015	41	447	45	452	2.5%	0.92 [0.62, 1.38]
Rosenstock 2015C	43	324	15	170	1.3%	1.50 [0.86, 2.63]
Wilding 2014	49	610	7	197	0.7%	2.26 [1.04, 4.91]
Zinman 2015	842	4687	423	2333	36.7%	0.99 [0.89, 1.10]
**Subtotal (95% CI)**		**10936**		**5995**	**66.3%**	**1.10 [0.98, 1.25]**
Total events	1582		816			
Heterogeneity: $Tau^2 = 0.01$; $Chi^2 = 17.51$, df = 12 (P = 0.13); $I^2 = 31\%$						
Test for overall effect: Z = 1.59 (P = 0.11)						
**Total (95% CI)**		**29900**		**14940**	**100.0%**	**1.05 [0.98, 1.12]**
Total events	2558		1278			
Heterogeneity: $Tau^2 = 0.00$; $Chi^2 = 50.47$, df = 65 (P = 0.91); $I^2 = 0\%$						
Test for overall effect: Z = 1.42 (P = 0.16)						
Test for subgroup differences: $Chi^2 = 0.50$, df = 2 (P = 0.78), $I^2 = 0\%$						

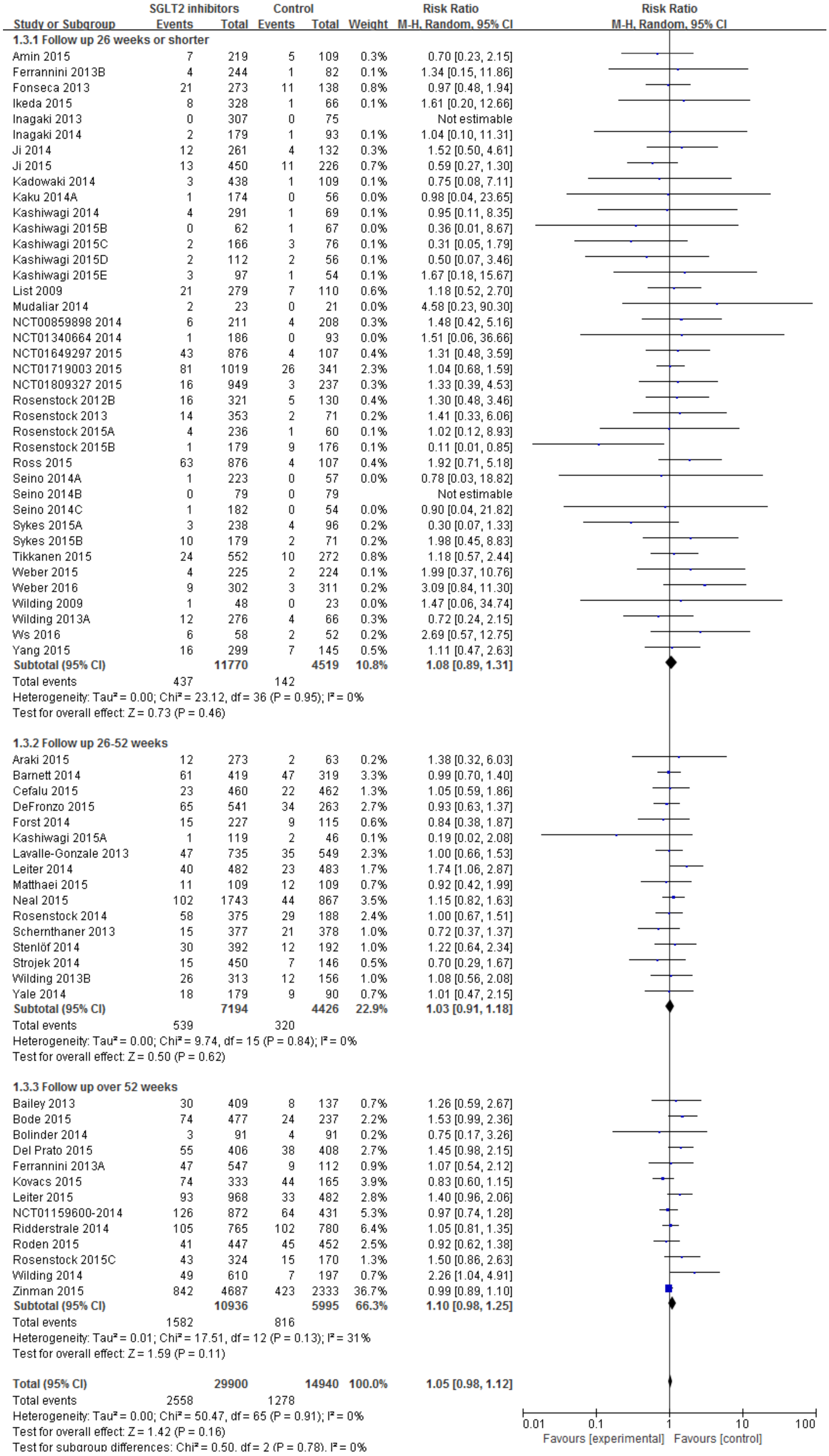

Figure 7S. Subgroup analysis of UTIs by length of follow up in randomized controlled trials

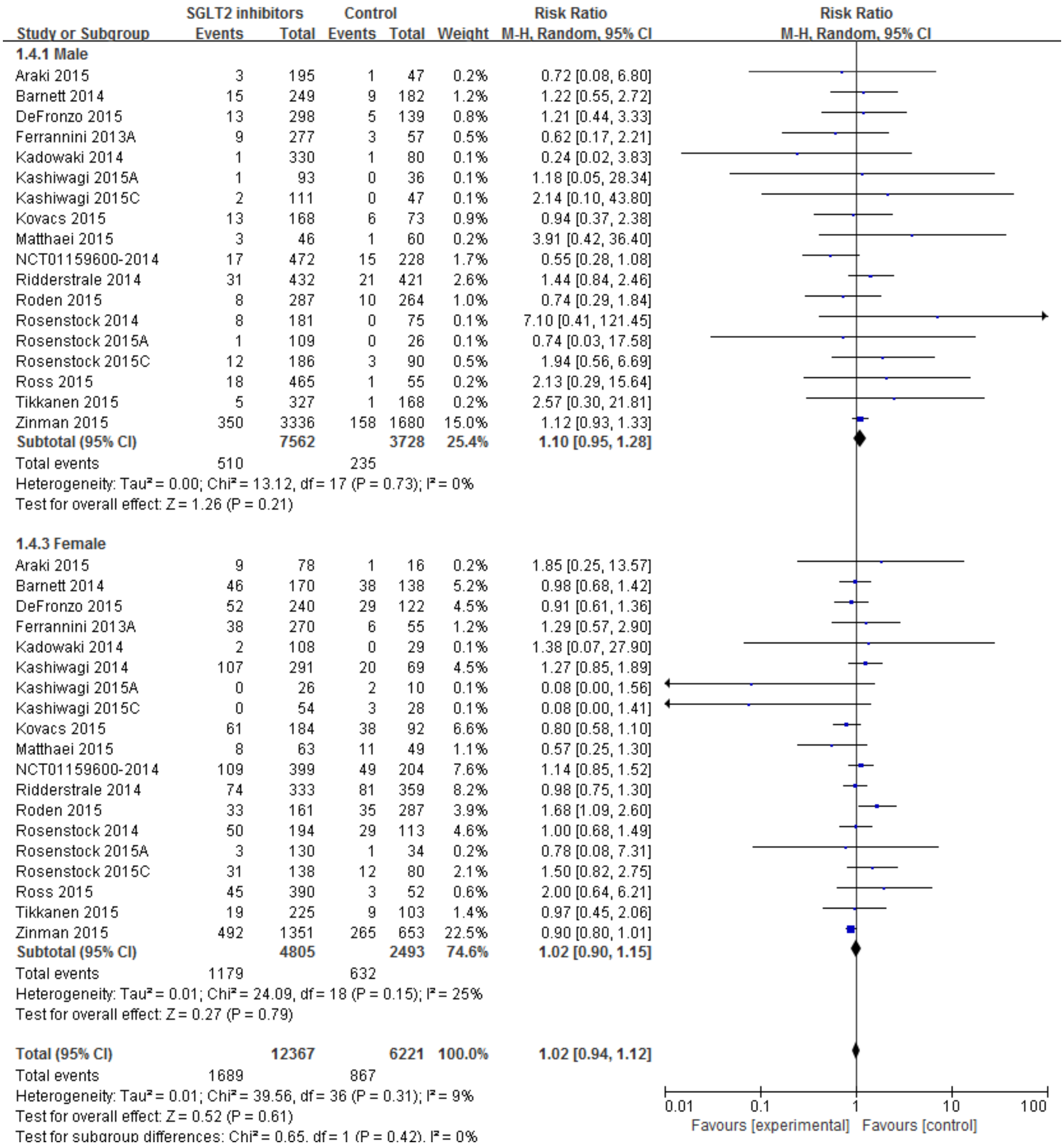

Figure 8S. Subgroup analysis of UTIs by gender in randomized controlled trials

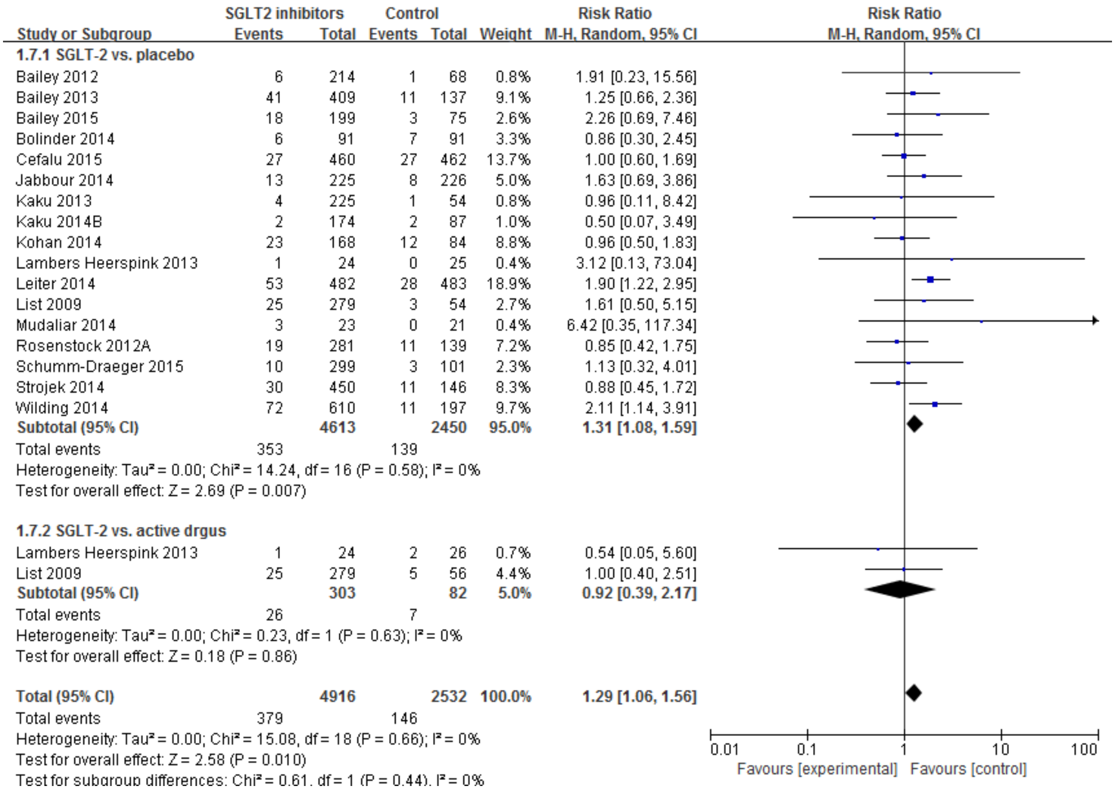

Figure 9S. Subgroup analysis of events suggestive of UTIs by type of control in randomized controlled trials

Study or Subgroup	SGLT2 inhibitors Events	SGLT2 inhibitors Total	Control Events	Control Total	Weight	Risk Ratio M-H, Random, 95% CI
**1.8.1 Male**						
Bailey 2012	2	104	1	37	0.7%	0.71 [0.07, 7.62]
Bailey 2013	8	216	3	76	2.3%	0.94 [0.26, 3.45]
Bailey 2015	5	101	0	31	0.5%	3.45 [0.20, 60.72]
Bolinder 2014	2	49	1	51	0.7%	2.08 [0.19, 22.23]
Cefalu 2015	10	311	10	316	5.3%	1.02 [0.43, 2.41]
Del Prato 2015	20	221	13	220	8.7%	1.53 [0.78, 3.00]
Leiter 2014	23	323	8	323	6.3%	2.88 [1.31, 6.33]
Strojek 2014	9	215	1	71	0.9%	2.97 [0.38, 23.05]
Wilding 2014	20	288	4	98	3.6%	1.70 [0.60, 4.86]
**Subtotal (95% CI)**		**1828**		**1223**	**29.1%**	**1.63 [1.12, 2.35]**
Total events	99		41			
Heterogeneity: Tau² = 0.00; Chi² = 5.01, df = 8 (P = 0.76); I² = 0%						
Test for overall effect: Z = 2.59 (P = 0.010)						
**1.8.3 Female**						
Bailey 2012	4	110	0	31	0.5%	2.59 [0.14, 46.93]
Bailey 2013	33	193	8	61	7.7%	1.30 [0.64, 2.67]
Bailey 2015	13	98	3	44	2.7%	1.95 [0.58, 6.49]
Bolinder 2014	4	42	6	40	2.8%	0.63 [0.19, 2.08]
Cefalu 2015	17	149	17	146	9.9%	0.98 [0.52, 1.84]
Del Prato 2015	35	179	25	181	17.9%	1.42 [0.88, 2.26]
Leiter 2014	30	159	20	160	14.5%	1.51 [0.90, 2.54]
Strojek 2014	21	235	10	75	7.9%	0.67 [0.33, 1.36]
Wilding 2014	52	322	7	99	6.9%	2.28 [1.07, 4.87]
**Subtotal (95% CI)**		**1487**		**837**	**70.9%**	**1.28 [0.99, 1.64]**
Total events	209		96			
Heterogeneity: Tau² = 0.01; Chi² = 8.81, df = 8 (P = 0.36); I² = 9%						
Test for overall effect: Z = 1.89 (P = 0.06)						
**Total (95% CI)**		**3315**		**2060**	**100.0%**	**1.37 [1.13, 1.68]**
Total events	308		137			
Heterogeneity: Tau² = 0.00; Chi² = 14.99, df = 17 (P = 0.60); I² = 0%						
Test for overall effect: Z = 3.13 (P = 0.002)						
Test for subgroup differences: Chi² = 1.12, df = 1 (P = 0.29), I² = 11.0%						

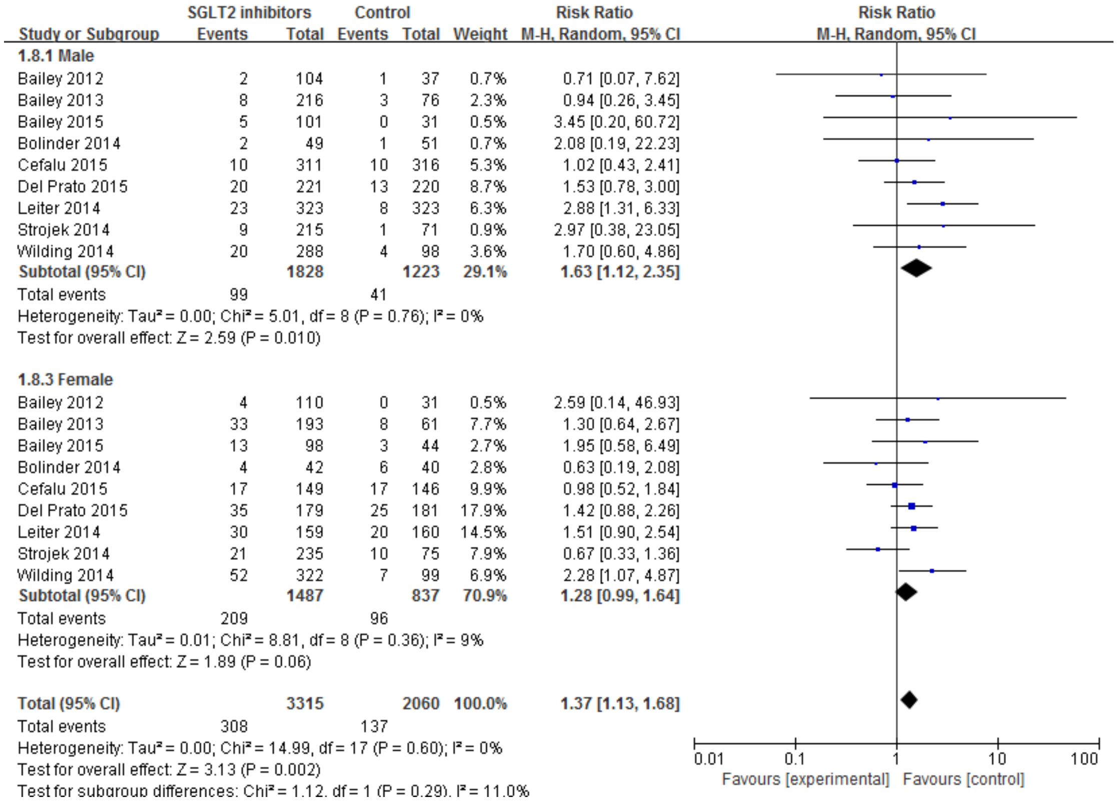

Figure 10S. Subgroup analysis of events suggestive of UTIs by gender in randomized controlled trials

Study or Subgroup	SGLT2 inhibitors Events	SGLT2 inhibitors Total	Control Events	Control Total	Weight	Risk Ratio M-H, Random, 95% CI
**1.9.1 Follow up 26 weeks or shorter**						
Bailey 2012	6	214	1	68	0.8%	1.91 [0.23, 15.56]
Kaku 2013	4	225	1	54	0.8%	0.96 [0.11, 8.42]
Kaku 2014B	2	174	2	87	1.0%	0.50 [0.07, 3.49]
Lambers Heerspink 2013	1	24	2	51	0.7%	1.06 [0.10, 11.15]
List 2009	25	279	8	110	6.4%	1.23 [0.57, 2.65]
Mudaliar 2014	3	23	0	21	0.4%	6.42 [0.35, 117.34]
Schumm-Draeger 2015	10	299	3	101	2.3%	1.13 [0.32, 4.01]
**Subtotal (95% CI)**		**1238**		**492**	**12.4%**	**1.20 [0.70, 2.08]**
Total events	51		17			
Heterogeneity: Tau² = 0.00; Chi² = 2.34, df = 6 (P = 0.89); I² = 0%						
Test for overall effect: Z = 0.66 (P = 0.51)						
**1.9.2 Follow up 26-52 weeks**						
Cefalu 2015	27	460	27	462	13.9%	1.00 [0.60, 1.69]
Jabbour 2014	13	225	8	226	5.0%	1.63 [0.69, 3.86]
Leiter 2014	53	482	28	483	19.2%	1.90 [1.22, 2.95]
Rosenstock 2012A	19	281	11	139	7.3%	0.85 [0.42, 1.75]
Strojek 2014	30	450	11	146	8.4%	0.88 [0.45, 1.72]
**Subtotal (95% CI)**		**1898**		**1456**	**53.7%**	**1.22 [0.86, 1.73]**
Total events	142		85			
Heterogeneity: Tau² = 0.06; Chi² = 6.63, df = 4 (P = 0.16); I² = 40%						
Test for overall effect: Z = 1.10 (P = 0.27)						
**1.9.3 Follow up over 52 weeks**						
Bailey 2013	41	409	11	137	9.2%	1.25 [0.66, 2.36]
Bailey 2015	18	199	3	75	2.6%	2.26 [0.69, 7.46]
Bolinder 2014	6	91	7	91	3.4%	0.86 [0.30, 2.45]
Kohan 2014	23	168	12	84	8.9%	0.96 [0.50, 1.83]
Wilding 2014	72	610	11	197	9.9%	2.11 [1.14, 3.91]
**Subtotal (95% CI)**		**1477**		**584**	**33.9%**	**1.37 [0.95, 1.97]**
Total events	160		44			
Heterogeneity: Tau² = 0.03; Chi² = 4.70, df = 4 (P = 0.32); I² = 15%						
Test for overall effect: Z = 1.67 (P = 0.09)						
**Total (95% CI)**		**4613**		**2532**	**100.0%**	**1.29 [1.06, 1.57]**
Total events	353		146			
Heterogeneity: Tau² = 0.00; Chi² = 13.84, df = 16 (P = 0.61); I² = 0%						
Test for overall effect: Z = 2.60 (P = 0.009)						
Test for subgroup differences: Chi² = 0.24, df = 2 (P = 0.89), I² = 0%						

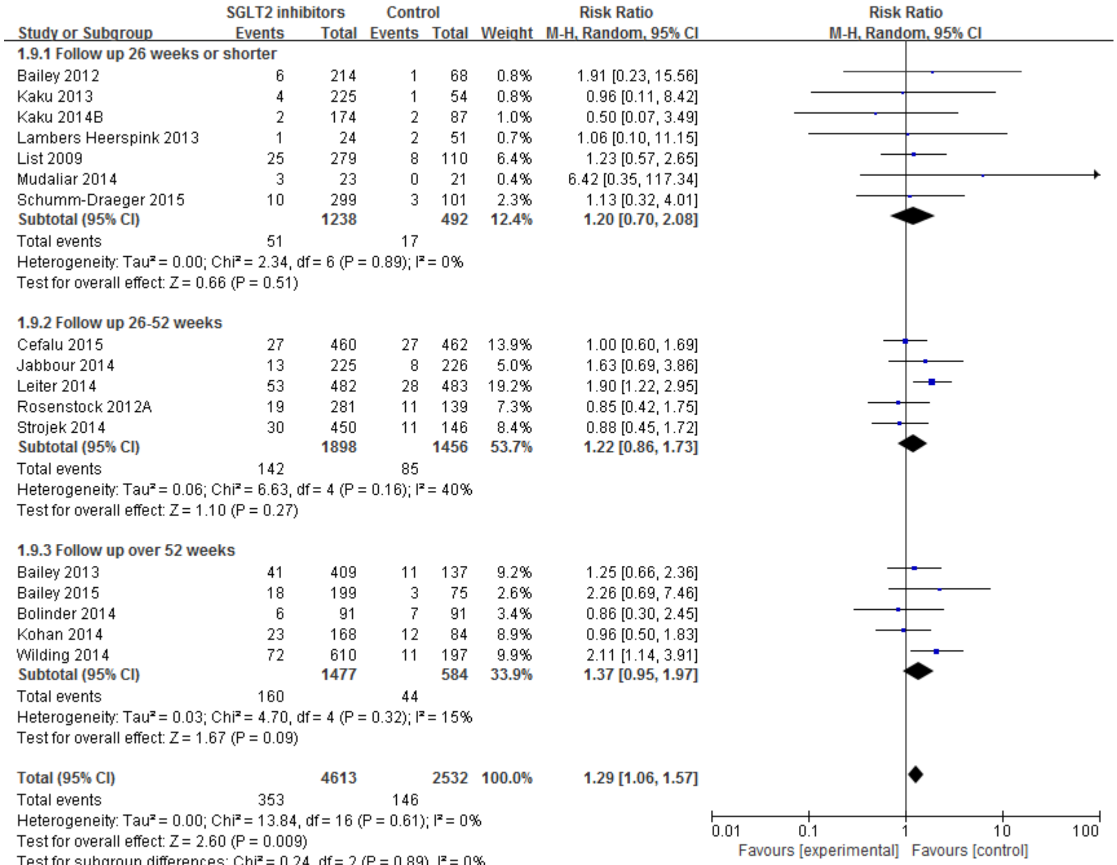

Figure 11S. Subgroup analysis of events suggestive of UTIs by length of follow up in randomized controlled trials

Study or Subgroup	SGLT2 inhibitors Events	SGLT2 inhibitors Total	Control Events	Control Total	Weight	Risk Ratio M-H, Random, 95% CI
**2.1.1 Follow up 26 weeks or shorter**						
Amin 2015	7	219	1	109	0.8%	3.48 [0.43, 27.96]
Ferrannini 2013B	5	244	0	82	0.4%	3.73 [0.21, 66.67]
Fonseca 2013	11	273	3	138	1.8%	1.85 [0.53, 6.53]
Inagaki 2013	2	307	0	75	0.4%	1.23 [0.06, 25.43]
Inagaki 2014	3	179	1	93	0.7%	1.56 [0.16, 14.78]
Ji 2014	10	261	1	132	0.8%	5.06 [0.65, 39.09]
Ji 2015	7	450	2	226	1.3%	1.76 [0.37, 8.39]
Kadowaki 2014	3	438	0	109	0.4%	1.75 [0.09, 33.71]
Kaku 2014A	1	174	0	56	0.3%	0.98 [0.04, 23.65]
Kashiwagi 2014	2	291	0	69	0.4%	1.20 [0.06, 24.69]
Kashiwagi 2015B	2	62	0	67	0.4%	5.40 [0.26, 110.25]
Kashiwagi 2015C	1	166	3	76	0.7%	0.15 [0.02, 1.44]
Kashiwagi 2015D	0	112	0	56		Not estimable
Kashiwagi 2015E	2	97	0	54	0.4%	2.81 [0.14, 57.41]
List 2009	12	279	1	110	0.8%	4.73 [0.62, 35.95]
NCT00859898 2014	7	211	1	208	0.8%	6.90 [0.86, 55.60]
Rosenstock 2012B	17	321	2	130	1.4%	3.44 [0.81, 14.69]
Rosenstock 2013	14	353	0	71	0.4%	5.90 [0.36, 97.75]
Rosenstock 2015A	8	236	0	60	0.4%	4.38 [0.26, 74.76]
Rosenstock 2015B	10	179	1	176	0.8%	9.83 [1.27, 76.00]
Ross 2015	33	876	3	107	2.1%	1.34 [0.42, 4.31]
Seino 2014A	0	223	1	57	0.3%	0.09 [0.00, 2.09]
Seino 2014B	1	79	1	79	0.4%	1.00 [0.06, 15.71]
Seino 2014C	2	182	0	54	0.4%	1.50 [0.07, 30.84]
Sykes 2015A	11	238	0	96	0.4%	9.33 [0.56, 156.85]
Sykes 2015B	8	179	0	71	0.4%	6.80 [0.40, 116.28]
Tikkanen 2015	29	552	1	272	0.8%	14.29 [1.96, 104.35]
Weber 2015	6	225	4	224	1.8%	1.49 [0.43, 5.22]
Weber 2016	6	302	5	311	2.0%	1.24 [0.38, 4.01]
Wilding 2009	5	48	1	23	0.7%	2.40 [0.30, 19.35]
Wilding 2013A	5	276	1	66	0.7%	1.20 [0.14, 10.06]
Ws 2016	3	58	3	52	1.3%	0.90 [0.19, 4.25]
Yang 2015	5	299	0	145	0.4%	5.35 [0.30, 96.16]
**Subtotal (95% CI)**		**8389**		**3654**	**25.1%**	**2.10 [1.47, 2.98]**
Total events	238		36			
Heterogeneity: Tau² = 0.00; Chi² = 26.87, df = 31 (P = 0.68); I² = 0%						
Test for overall effect: Z = 4.12 (P < 0.0001)						
**2.1.2 Follow up 26-52 weeks**						
Araki 2015	2	273	1	63	0.6%	0.46 [0.04, 5.01]
Barnett 2014	18	419	8	319	3.5%	1.71 [0.75, 3.89]
DeFronzo 2015	23	541	7	263	3.4%	1.60 [0.69, 3.67]
Forst 2014	23	227	3	115	2.0%	3.88 [1.19, 12.67]
Kashiwagi 2015A	1	119	0	46	0.3%	1.18 [0.05, 28.33]
Lavalle-Gonzale 2013	55	735	9	549	4.3%	4.56 [2.28, 9.16]
Matthaei 2015	11	109	1	109	0.8%	11.00 [1.44, 83.74]
Neal 2015	191	1743	17	867	6.1%	5.59 [3.43, 9.12]
Rosenstock 2014	26	375	3	188	2.0%	4.34 [1.33, 14.17]
Schernthaner 2013	45	377	8	378	4.0%	5.64 [2.70, 11.80]
Stenlöf 2014	36	392	5	192	3.0%	3.53 [1.41, 8.84]
Wilding 2013B	39	313	5	156	3.0%	3.89 [1.56, 9.67]
Yale 2014	4	179	3	90	1.4%	0.67 [0.15, 2.93]
**Subtotal (95% CI)**		**5802**		**3335**	**34.4%**	**3.26 [2.24, 4.74]**
Total events	474		70			
Heterogeneity: Tau² = 0.19; Chi² = 21.77, df = 12 (P = 0.04); I² = 45%						
Test for overall effect: Z = 6.19 (P < 0.00001)						
**2.1.3 Follow up over 52 weeks**						
Bode 2015	69	477	6	237	3.5%	5.71 [2.52, 12.97]
Del Prato 2015	58	406	12	408	5.0%	4.86 [2.65, 8.91]
Ferrannini 2013A	42	547	5	112	3.0%	1.72 [0.70, 4.25]
Kovacs 2015	24	333	5	165	2.9%	2.38 [0.92, 6.12]
Leiter 2015	116	968	11	482	5.0%	5.25 [2.86, 9.65]
NCT01159600-2014	61	872	3	431	2.1%	10.05 [3.17, 31.85]
Ridderstrale 2014	90	765	17	780	5.9%	5.40 [3.25, 8.98]
Roden 2015	27	447	6	452	3.2%	4.55 [1.90, 10.91]
Rosenstock 2015C	21	324	3	170	2.0%	3.67 [1.11, 12.14]
Zinman 2015	301	4687	42	2333	8.0%	3.57 [2.59, 4.91]
**Subtotal (95% CI)**		**9826**		**5570**	**40.5%**	**4.23 [3.36, 5.33]**
Total events	809		110			
Heterogeneity: Tau² = 0.02; Chi² = 10.70, df = 9 (P = 0.30); I² = 16%						
Test for overall effect: Z = 12.24 (P < 0.00001)						
**Total (95% CI)**		**24017**		**12559**	**100.0%**	**3.30 [2.74, 3.99]**
Total events	1521		216			
Heterogeneity: Tau² = 0.09; Chi² = 69.66, df = 54 (P = 0.07); I² = 22%						
Test for overall effect: Z = 12.48 (P < 0.00001)						
Test for subgroup differences: Chi² = 10.68, df = 2 (P = 0.005), I² = 81.3%						

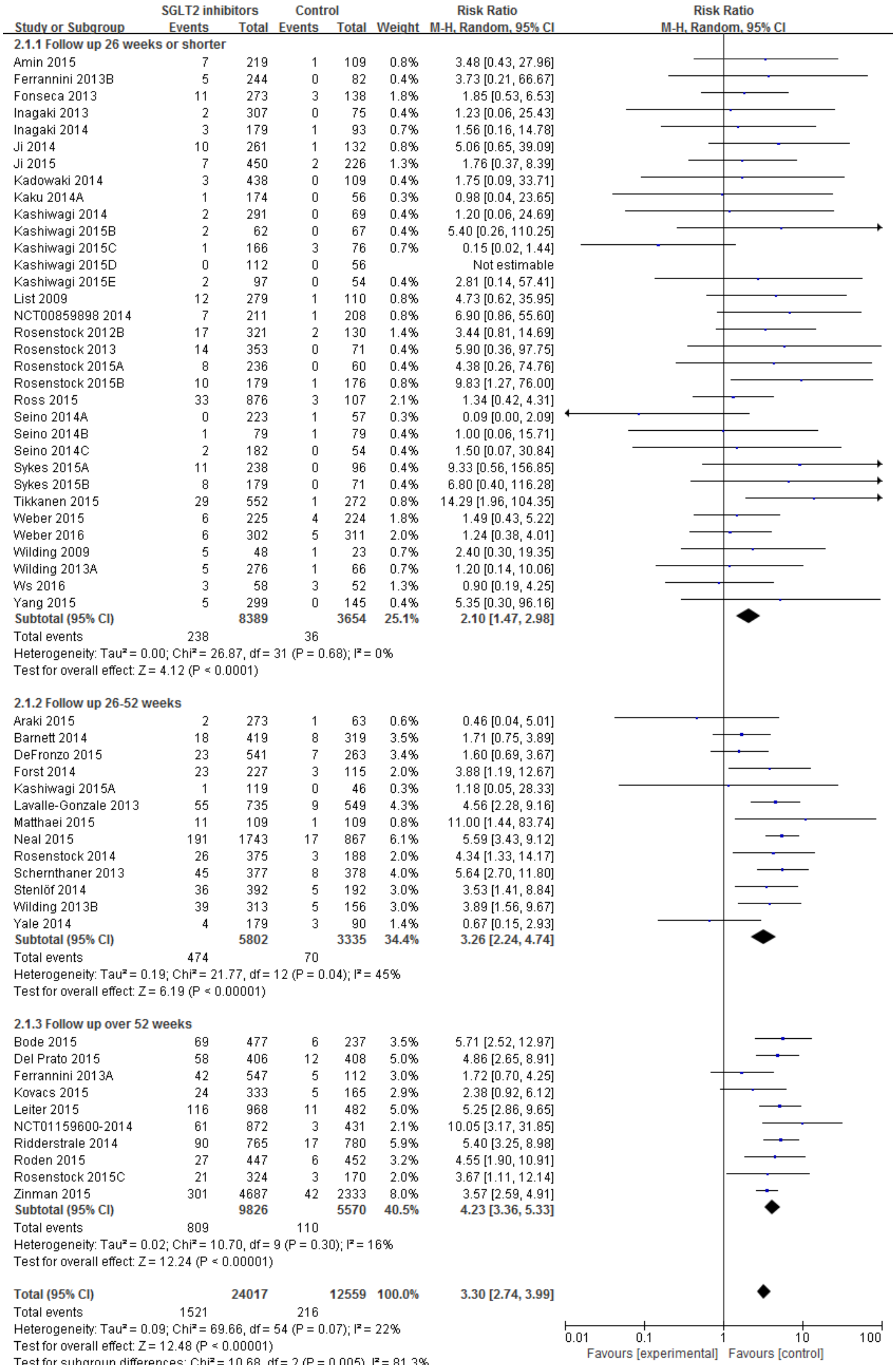

Figure 12S. Subgroup analysis of genital infections by length of follow up in randomized controlled trials

Study or Subgroup	SGLT2 inhibitors Events	SGLT2 inhibitors Total	Control Events	Control Total	Weight	Risk Ratio M-H, Random, 95% CI
**2.2.1 SGLT-2 vs. placebo**						
Amin 2015	7	219	1	55	0.6%	1.76 [0.22, 13.99]
Barnett 2014	18	419	8	319	3.2%	1.71 [0.75, 3.89]
Bode 2015	69	477	6	237	3.2%	5.71 [2.52, 12.97]
DeFronzo 2015	23	547	7	263	3.1%	1.58 [0.69, 3.63]
Ferrannini 2013B	5	244	0	82	0.3%	3.73 [0.21, 66.67]
Fonseca 2013	11	273	1	69	0.7%	2.78 [0.37, 21.17]
Inagaki 2013	2	307	0	75	0.3%	1.23 [0.06, 25.43]
Inagaki 2014	3	179	1	93	0.5%	1.56 [0.16, 14.78]
Ji 2014	10	261	1	132	0.7%	5.06 [0.65, 39.09]
Ji 2015	7	450	2	226	1.1%	1.76 [0.37, 8.39]
Kadowaki 2014	3	438	0	109	0.3%	1.75 [0.09, 33.71]
Kaku 2014A	1	174	0	56	0.3%	0.98 [0.04, 23.65]
Kashiwagi 2014	2	291	0	69	0.3%	1.20 [0.06, 24.69]
Kashiwagi 2015A	1	119	0	46	0.3%	1.18 [0.05, 28.33]
Kashiwagi 2015B	2	62	0	67	0.3%	5.40 [0.26, 110.25]
Kashiwagi 2015C	1	166	3	76	0.5%	0.15 [0.02, 1.44]
Kashiwagi 2015D	0	112	0	56		Not estimable
Kashiwagi 2015E	2	97	0	54	0.3%	2.81 [0.14, 57.41]
Kovacs 2015	24	333	5	165	2.6%	2.38 [0.92, 6.12]
List 2009	12	279	0	54	0.4%	4.91 [0.30, 81.72]
Matthaei 2015	11	109	1	109	0.7%	11.00 [1.44, 83.74]
NCT00859898 2014	7	211	1	208	0.6%	6.90 [0.86, 55.60]
NCT01159600-2014	61	872	3	431	1.9%	10.05 [3.17, 31.85]
Neal 2015	191	1743	17	867	6.3%	5.59 [3.43, 9.12]
Roden 2015	27	447	4	229	2.2%	3.46 [1.22, 9.76]
Rosenstock 2012B	17	321	1	65	0.7%	3.44 [0.47, 25.41]
Rosenstock 2013	14	353	0	71	0.4%	5.90 [0.36, 97.75]
Rosenstock 2014	26	375	3	188	1.8%	4.34 [1.33, 14.17]
Rosenstock 2015A	8	236	0	60	0.3%	4.38 [0.26, 74.76]
Rosenstock 2015B	0	179	1	176	0.3%	0.33 [0.01, 7.99]
Rosenstock 2015C	21	324	3	170	1.7%	3.67 [1.11, 12.14]
Ross 2015	33	876	3	107	1.8%	1.34 [0.42, 4.31]
Seino 2014A	0	223	1	57	0.3%	0.09 [0.00, 2.09]
Seino 2014B	1	79	1	79	0.4%	1.00 [0.06, 15.71]
Seino 2014C	2	182	0	54	0.3%	1.50 [0.07, 30.84]
Sykes 2015A	11	238	0	48	0.4%	4.72 [0.28, 78.69]
Sykes 2015B	8	179	0	36	0.4%	3.49 [0.21, 59.22]
Tikkanen 2015	29	552	1	272	0.7%	14.29 [1.96, 104.35]
Weber 2015	6	225	4	224	1.6%	1.49 [0.43, 5.22]
Weber 2016	6	302	5	311	1.8%	1.24 [0.38, 4.01]
Wilding 2009	5	48	1	23	0.6%	2.40 [0.30, 19.35]
Wilding 2013A	5	276	1	66	0.6%	1.20 [0.14, 10.06]
Wilding 2013B	39	313	5	156	2.7%	3.89 [1.56, 9.67]
Yale 2014	4	179	3	90	1.2%	0.67 [0.15, 2.93]
Yang 2015	5	299	0	145	0.3%	5.35 [0.30, 96.16]
Zinman 2015	301	4687	42	2333	8.9%	3.57 [2.59, 4.91]
**Subtotal (95% CI)**		**19275**		**8878**	**58.0%**	**2.87 [2.27, 3.62]**
Total events	1041		136			
Heterogeneity: Tau² = 0.08; Chi² = 52.43, df = 44 (P = 0.18); I² = 16%						
Test for overall effect: Z = 8.84 (P < 0.00001)						
**2.2.2 SGLT-2 vs. active drugs**						
Amin 2015	7	219	0	55	0.3%	3.82 [0.22, 65.85]
Araki 2015	2	273	1	63	0.5%	0.46 [0.04, 5.01]
DeFronzo 2015	36	547	7	263	3.4%	2.47 [1.12, 5.48]
Del Prato 2015	58	406	12	408	4.9%	4.86 [2.65, 8.91]
Ferrannini 2013A	42	547	5	112	2.8%	1.72 [0.70, 4.25]
Fonseca 2013	11	273	2	69	1.2%	1.39 [0.32, 6.13]
Forst 2014	23	227	3	115	1.8%	3.88 [1.19, 12.67]
Lavalle-Gonzale 2013	55	735	9	549	4.1%	4.56 [2.28, 9.16]
Leiter 2015	116	968	11	482	4.9%	5.25 [2.86, 9.65]
List 2009	12	279	1	56	0.7%	2.41 [0.32, 18.15]
NCT00859898 2014	11	219	1	208	0.7%	10.45 [1.36, 80.21]
Ridderstrale 2014	90	765	17	780	6.0%	5.40 [3.25, 8.98]
Roden 2015	27	447	2	223	1.3%	6.73 [1.62, 28.07]
Rosenstock 2012B	17	321	1	65	0.7%	3.44 [0.47, 25.41]
Rosenstock 2015B	10	179	1	176	0.7%	9.83 [1.27, 76.00]
Schernthaner 2013	45	377	8	378	3.8%	5.64 [2.70, 11.80]
Stenlöf 2014	36	392	5	192	2.7%	3.53 [1.41, 8.84]
Sykes 2015A	11	238	0	48	0.4%	4.72 [0.28, 78.69]
Sykes 2015B	8	179	0	35	0.4%	3.40 [0.20, 57.59]
Ws 2016	3	58	3	52	1.1%	0.90 [0.19, 4.25]
**Subtotal (95% CI)**		**7649**		**4329**	**42.0%**	**4.06 [3.24, 5.08]**
Total events	620		89			
Heterogeneity: Tau² = 0.01; Chi² = 19.42, df = 19 (P = 0.43); I² = 2%						
Test for overall effect: Z = 12.23 (P < 0.00001)						
**Total (95% CI)**		**26924**		**13207**	**100.0%**	**3.30 [2.78, 3.91]**
Total events	1661		225			
Heterogeneity: Tau² = 0.06; Chi² = 74.82, df = 64 (P = 0.17); I² = 14%						
Test for overall effect: Z = 13.77 (P < 0.00001)						
Test for subgroup differences: Chi² = 4.41, df = 1 (P = 0.04), I² = 77.3%						

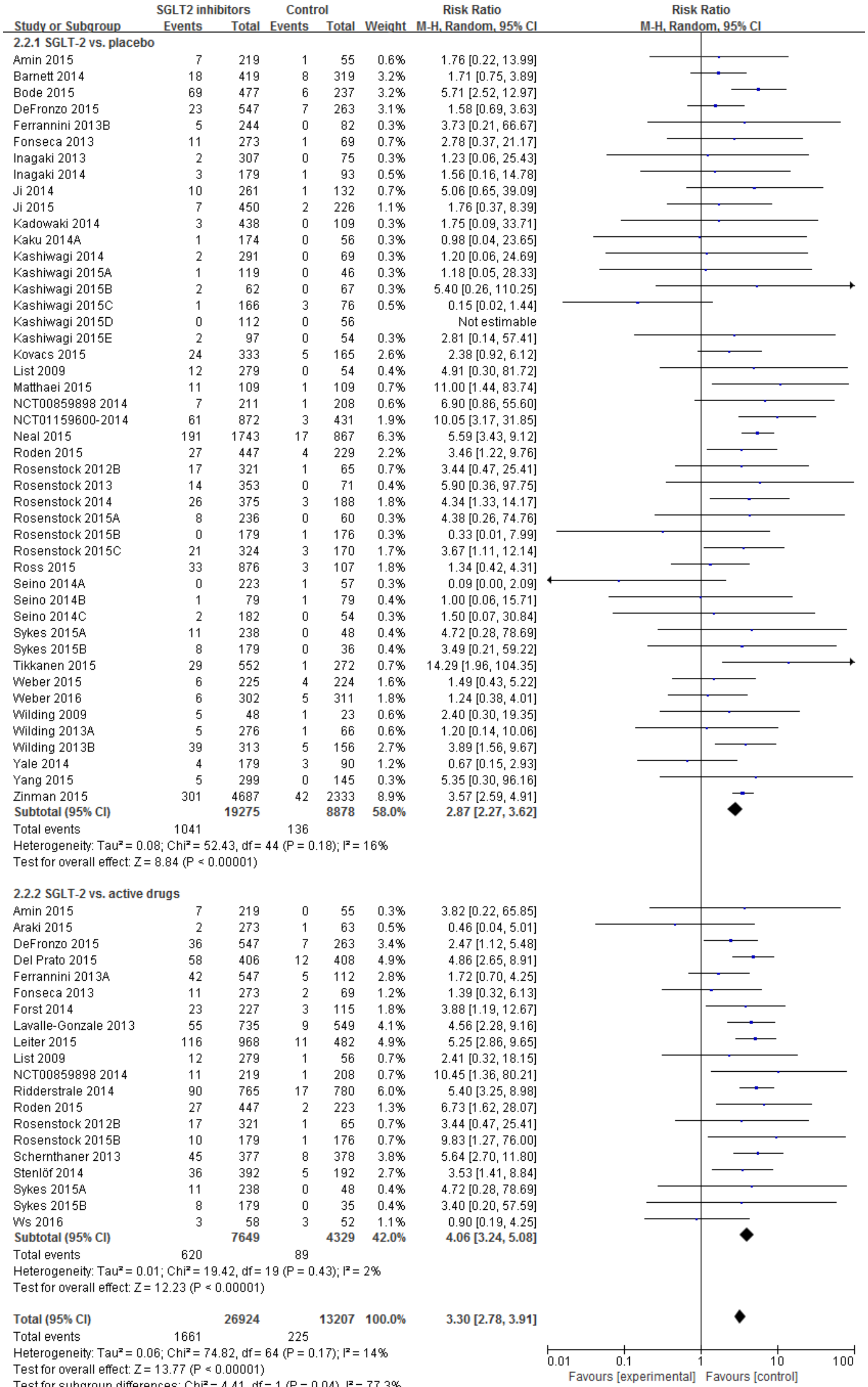

Figure 13S. Subgroup analysis of genital infections by type of control in randomized controlled trials

Study or Subgroup	SGLT2 inhibitors Events	SGLT2 inhibitors Total	Control Events	Control Total	Weight	Risk Ratio M-H, Random, 95% CI
**2.3.1 Canagliflozin**						
Bode 2015	69	477	6	237	3.5%	5.71 [2.52, 12.97]
Forst 2014	23	227	3	115	2.0%	3.88 [1.19, 12.67]
Inagaki 2013	2	307	0	75	0.4%	1.23 [0.06, 25.43]
Inagaki 2014	3	179	1	93	0.7%	1.56 [0.16, 14.78]
Ji 2015	7	450	2	226	1.3%	1.76 [0.37, 8.39]
Lavalle-Gonzale 2013	55	735	9	549	4.3%	4.56 [2.28, 9.16]
Leiter 2015	116	968	11	482	5.0%	5.25 [2.86, 9.65]
Neal 2015	191	1743	17	867	6.1%	5.59 [3.43, 9.12]
Rosenstock 2012B	17	321	2	130	1.4%	3.44 [0.81, 14.69]
Schernthaner 2013	45	377	8	378	4.0%	5.64 [2.70, 11.80]
Stenlöf 2014	36	392	5	192	3.0%	3.53 [1.41, 8.84]
Wilding 2013B	39	313	5	156	3.0%	3.89 [1.56, 9.67]
Yale 2014	4	179	3	90	1.4%	0.67 [0.15, 2.93]
**Subtotal (95% CI)**		**6668**		**3590**	**35.9%**	**4.45 [3.49, 5.67]**
Total events	607		72			
Heterogeneity: Tau² = 0.00; Chi² = 11.66, df = 12 (P = 0.47); I² = 0%						
Test for overall effect: Z = 12.07 (P < 0.00001)						
**2.3.2 Dapagliflozin**						
Del Prato 2015	58	406	12	408	5.0%	4.86 [2.65, 8.91]
Ji 2014	10	261	1	132	0.8%	5.06 [0.65, 39.09]
List 2009	12	279	1	110	0.8%	4.73 [0.62, 35.95]
Matthaei 2015	11	109	1	109	0.8%	11.00 [1.44, 83.74]
NCT00859898 2014	7	211	1	208	0.8%	6.90 [0.86, 55.60]
Rosenstock 2015B	10	179	1	176	0.8%	9.83 [1.27, 76.00]
Weber 2015	6	225	4	224	1.8%	1.49 [0.43, 5.22]
Weber 2016	6	302	5	311	2.0%	1.24 [0.38, 4.01]
Wilding 2009	5	48	1	23	0.7%	2.40 [0.30, 19.35]
Ws 2016	3	58	3	52	1.3%	0.90 [0.19, 4.25]
Yang 2015	5	299	0	145	0.4%	5.35 [0.30, 96.16]
**Subtotal (95% CI)**		**2377**		**1898**	**15.2%**	**3.22 [1.95, 5.32]**
Total events	133		30			
Heterogeneity: Tau² = 0.12; Chi² = 12.15, df = 10 (P = 0.28); I² = 18%						
Test for overall effect: Z = 4.56 (P < 0.00001)						
**2.3.3 Empagliflozin**						
Araki 2015	2	273	1	63	0.6%	0.46 [0.04, 5.01]
Barnett 2014	18	419	8	319	3.5%	1.71 [0.75, 3.89]
DeFronzo 2015	23	541	7	263	3.4%	1.60 [0.69, 3.67]
Ferrannini 2013A	42	547	5	112	3.0%	1.72 [0.70, 4.25]
Ferrannini 2013B	5	244	0	82	0.4%	3.73 [0.21, 66.67]
Kadowaki 2014	3	438	0	109	0.4%	1.75 [0.09, 33.71]
Kovacs 2015	24	333	5	165	2.9%	2.38 [0.92, 6.12]
NCT01159600-2014	61	872	3	431	2.1%	10.05 [3.17, 31.85]
Ridderstrale 2014	90	765	17	780	5.9%	5.40 [3.25, 8.98]
Roden 2015	27	447	6	452	3.2%	4.55 [1.90, 10.91]
Rosenstock 2013	14	353	0	71	0.4%	5.90 [0.36, 97.75]
Rosenstock 2014	26	375	3	188	2.0%	4.34 [1.33, 14.17]
Rosenstock 2015C	21	324	3	170	2.0%	3.67 [1.11, 12.14]
Ross 2015	33	876	3	107	2.1%	1.34 [0.42, 4.31]
Tikkanen 2015	29	552	1	272	0.8%	14.29 [1.96, 104.35]
Zinman 2015	301	4687	42	2333	8.0%	3.57 [2.59, 4.91]
**Subtotal (95% CI)**		**12046**		**5917**	**40.7%**	**3.14 [2.29, 4.30]**
Total events	719		104			
Heterogeneity: Tau² = 0.12; Chi² = 23.79, df = 15 (P = 0.07); I² = 37%						
Test for overall effect: Z = 7.12 (P < 0.00001)						
**2.3.5 Ipragliflozin**						
Fonseca 2013	11	273	3	138	1.8%	1.85 [0.53, 6.53]
Kashiwagi 2014	2	291	0	69	0.4%	1.20 [0.06, 24.69]
Kashiwagi 2015A	1	119	0	46	0.3%	1.18 [0.05, 28.33]
Kashiwagi 2015B	2	62	0	67	0.4%	5.40 [0.26, 110.25]
Kashiwagi 2015C	1	166	3	76	0.7%	0.15 [0.02, 1.44]
Kashiwagi 2015D	0	112	0	56		Not estimable
Kashiwagi 2015E	2	97	0	54	0.4%	2.81 [0.14, 57.41]
Wilding 2013A	5	276	1	66	0.7%	1.20 [0.14, 10.06]
**Subtotal (95% CI)**		**1396**		**572**	**4.7%**	**1.30 [0.57, 2.97]**
Total events	24		7			
Heterogeneity: Tau² = 0.00; Chi² = 4.91, df = 6 (P = 0.55); I² = 0%						
Test for overall effect: Z = 0.63 (P = 0.53)						
**2.3.6 Other agents**						
Amin 2015	7	219	1	109	0.8%	3.48 [0.43, 27.96]
Kaku 2014A	1	174	0	56	0.3%	0.98 [0.04, 23.65]
Rosenstock 2015A	8	236	0	60	0.4%	4.38 [0.26, 74.76]
Seino 2014A	0	223	1	57	0.3%	0.09 [0.00, 2.09]
Seino 2014B	1	79	1	79	0.4%	1.00 [0.06, 15.71]
Seino 2014C	2	182	0	54	0.4%	1.50 [0.07, 30.84]
Sykes 2015A	11	238	0	96	0.4%	9.33 [0.56, 156.85]
Sykes 2015B	8	179	0	71	0.4%	6.80 [0.40, 116.28]
**Subtotal (95% CI)**		**1530**		**582**	**3.5%**	**2.13 [0.80, 5.68]**
Total events	38		3			
Heterogeneity: Tau² = 0.00; Chi² = 6.82, df = 7 (P = 0.45); I² = 0%						
Test for overall effect: Z = 1.52 (P = 0.13)						
**Total (95% CI)**		**24017**		**12559**	**100.0%**	**3.30 [2.74, 3.99]**
Total events	1521		216			
Heterogeneity: Tau² = 0.09; Chi² = 69.66, df = 54 (P = 0.07); I² = 22%						
Test for overall effect: Z = 12.48 (P < 0.00001)						
Test for subgroup differences: Chi² = 10.82, df = 4 (P = 0.03), I² = 63.0%						

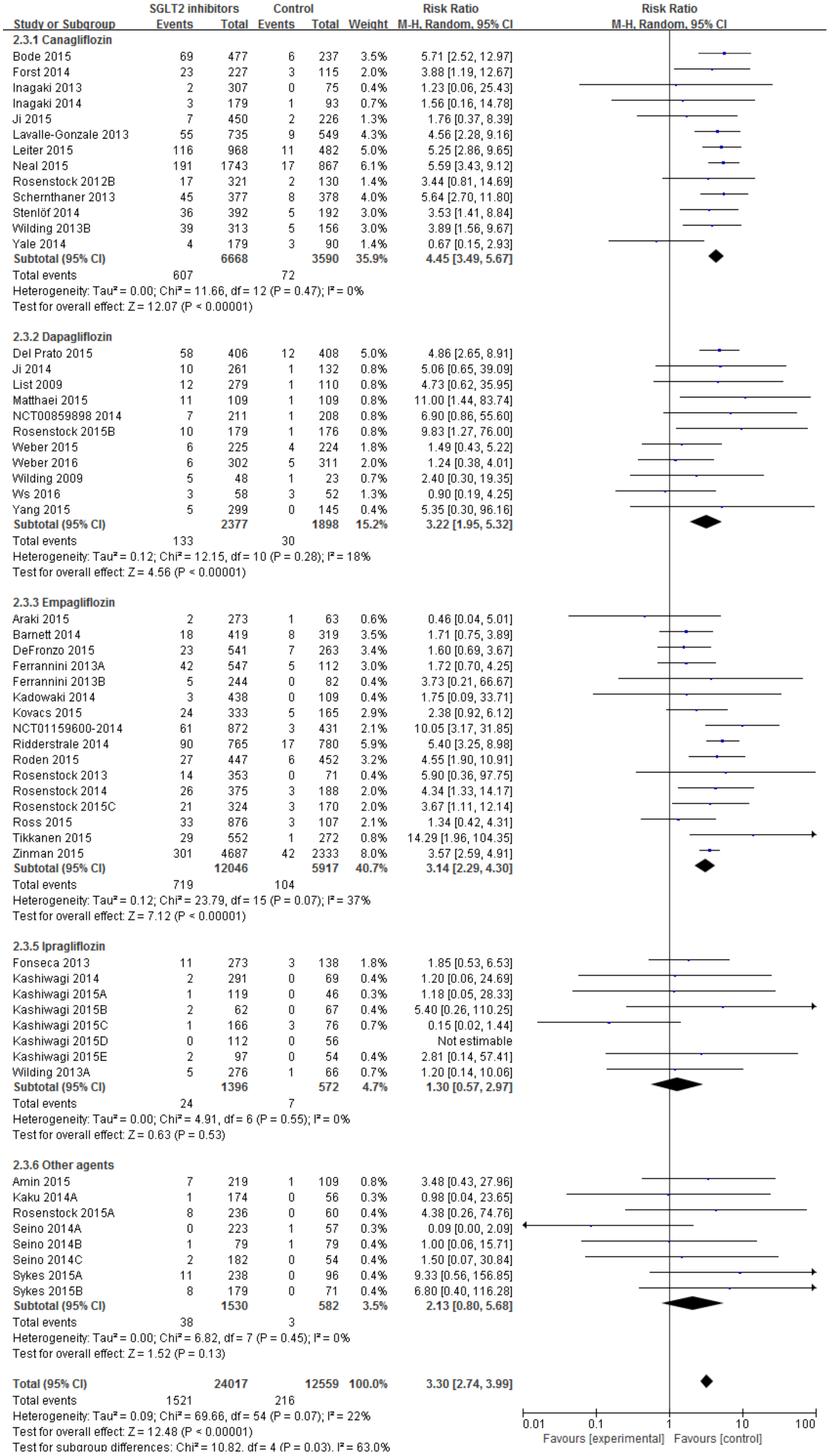

Figure 14S. Subgroup analysis of genital infections by individual SGLT2 inhibitors in randomized controlled trials

Study or Subgroup	Experimental Events	Experimental Total	Control Events	Control Total	Weight	Risk Ratio M-H, Random, 95% CI
**2.4.1 Male**						
Araki 2015	2	195	1	47	0.6%	0.48 [0.04, 5.20]
Barnett 2014	8	249	3	182	1.8%	1.95 [0.52, 7.25]
Bode 2015	21	253	2	143	1.6%	5.93 [1.41, 24.94]
DeFronzo 2015	10	298	3	139	1.9%	1.55 [0.43, 5.56]
Ferrannini 2013A	10	277	0	57	0.5%	4.38 [0.26, 73.72]
Forst 2014	6	140	0	76	0.4%	7.10 [0.41, 124.34]
Inagaki 2014	0	132	1	60	0.4%	0.15 [0.01, 3.70]
Ji 2015	2	237	0	125	0.4%	2.65 [0.13, 54.71]
Kadowaki 2014	3	330	0	80	0.4%	1.71 [0.09, 32.83]
Kashiwagi 2015A	0	93	0	36		Not estimable
Kashiwagi 2015C	0	111	1	47	0.4%	0.14 [0.01, 3.44]
Kovacs 2015	8	168	1	73	0.8%	3.48 [0.44, 27.29]
Lavalle-Gonzale 2013	13	339	3	266	2.0%	3.40 [0.98, 11.81]
Leiter 2015	46	493	5	263	3.2%	4.91 [1.97, 12.20]
Matthaei 2015	2	46	0	60	0.4%	6.49 [0.32, 131.97]
NCT01159600-2014	16	472	1	228	0.9%	7.73 [1.03, 57.92]
Neal 2015	101	1151	9	563	4.7%	5.49 [2.80, 10.77]
Ridderstrale 2014	41	432	5	421	3.2%	7.99 [3.19, 20.03]
Roden 2015	8	287	3	264	1.8%	2.45 [0.66, 9.15]
Rosenstock 2014	8	181	1	75	0.8%	3.31 [0.42, 26.04]
Rosenstock 2015C	11	186	0	90	0.5%	11.19 [0.67, 187.84]
Ross 2015	9	465	1	55	0.8%	1.06 [0.14, 8.24]
Schernthaner 2013	19	207	1	215	0.9%	19.73 [2.67, 146.08]
Stenlöf 2014	13	170	0	88	0.5%	14.05 [0.85, 233.65]
Tikkanen 2015	14	327	1	168	0.9%	7.19 [0.95, 54.23]
Wilding 2013B	11	163	1	76	0.8%	5.13 [0.67, 39.01]
Yale 2014	2	106	2	57	0.9%	0.54 [0.08, 3.72]
Zinman 2015	166	3336	25	1680	7.3%	3.34 [2.21, 5.07]
**Subtotal (95% CI)**		**10844**		**5634**	**38.8%**	**3.62 [2.66, 4.93]**
Total events	550		70			
Heterogeneity: Tau² = 0.08; Chi² = 30.43, df = 26 (P = 0.25); I² = 15%						
Test for overall effect: Z = 8.15 (P < 0.00001)						
**2.4.3 Female**						
Araki 2015	0	78	0	16		Not estimable
Barnett 2014	10	170	5	138	2.6%	1.62 [0.57, 4.64]
Bode 2015	48	224	4	94	2.8%	5.04 [1.87, 13.57]
DeFronzo 2015	13	240	4	122	2.4%	1.65 [0.55, 4.96]
Ferrannini 2013A	12	270	1	55	0.9%	2.44 [0.32, 18.41]
Forst 2014	17	87	3	39	2.2%	2.54 [0.79, 8.17]
Inagaki 2014	3	47	0	33	0.4%	4.96 [0.26, 92.90]
Ji 2015	5	213	2	101	1.3%	1.19 [0.23, 6.01]
Kadowaki 2014	0	108	0	29		Not estimable
Kashiwagi 2015A	1	26	2	10	0.7%	0.19 [0.02, 1.89]
Kashiwagi 2015C	1	54	2	28	0.6%	0.26 [0.02, 2.74]
Kovacs 2015	16	184	4	92	2.6%	2.00 [0.69, 5.81]
Lavalle-Gonzale 2013	42	396	6	283	3.6%	5.00 [2.16, 11.61]
Leiter 2015	70	475	6	219	3.7%	5.38 [2.37, 12.19]
Matthaei 2015	9	63	1	49	0.8%	7.00 [0.92, 53.40]
NCT01159600-2014	45	399	2	204	1.6%	11.50 [2.82, 46.94]
Neal 2015	90	592	8	304	4.5%	5.78 [2.84, 11.75]
Ridderstrale 2014	49	333	12	359	5.2%	4.40 [2.38, 8.13]
Roden 2015	19	161	3	287	2.1%	11.29 [3.39, 37.57]
Rosenstock 2014	18	194	2	113	1.6%	5.24 [1.24, 22.18]
Rosenstock 2015A	10	130	1	34	0.9%	2.62 [0.35, 19.73]
Rosenstock 2015C	10	138	3	80	2.0%	1.93 [0.55, 6.82]
Ross 2015	24	390	2	52	1.6%	1.60 [0.39, 6.57]
Schernthaner 2013	26	170	7	163	3.8%	3.56 [1.59, 7.98]
Stenlöf 2014	23	222	5	104	3.1%	2.15 [0.84, 5.51]
Tikkanen 2015	15	225	0	103	0.5%	14.27 [0.86, 236.14]
Wilding 2013B	28	150	4	80	2.8%	3.73 [1.36, 10.27]
Yale 2014	2	73	1	33	0.6%	0.90 [0.08, 9.62]
Zinman 2015	135	1351	17	653	6.4%	3.84 [2.34, 6.30]
**Subtotal (95% CI)**		**7163**		**3877**	**61.2%**	**3.38 [2.61, 4.37]**
Total events	741		107			
Heterogeneity: Tau² = 0.11; Chi² = 35.91, df = 26 (P = 0.09); I² = 28%						
Test for overall effect: Z = 9.24 (P < 0.00001)						
**Total (95% CI)**		**18007**		**9511**	**100.0%**	**3.48 [2.87, 4.23]**
Total events	1291		177			
Heterogeneity: Tau² = 0.09; Chi² = 66.40, df = 53 (P = 0.10); I² = 20%						
Test for overall effect: Z = 12.57 (P < 0.00001)						
Test for subgroup differences: Chi² = 0.11, df = 1 (P = 0.74), I² = 0%						

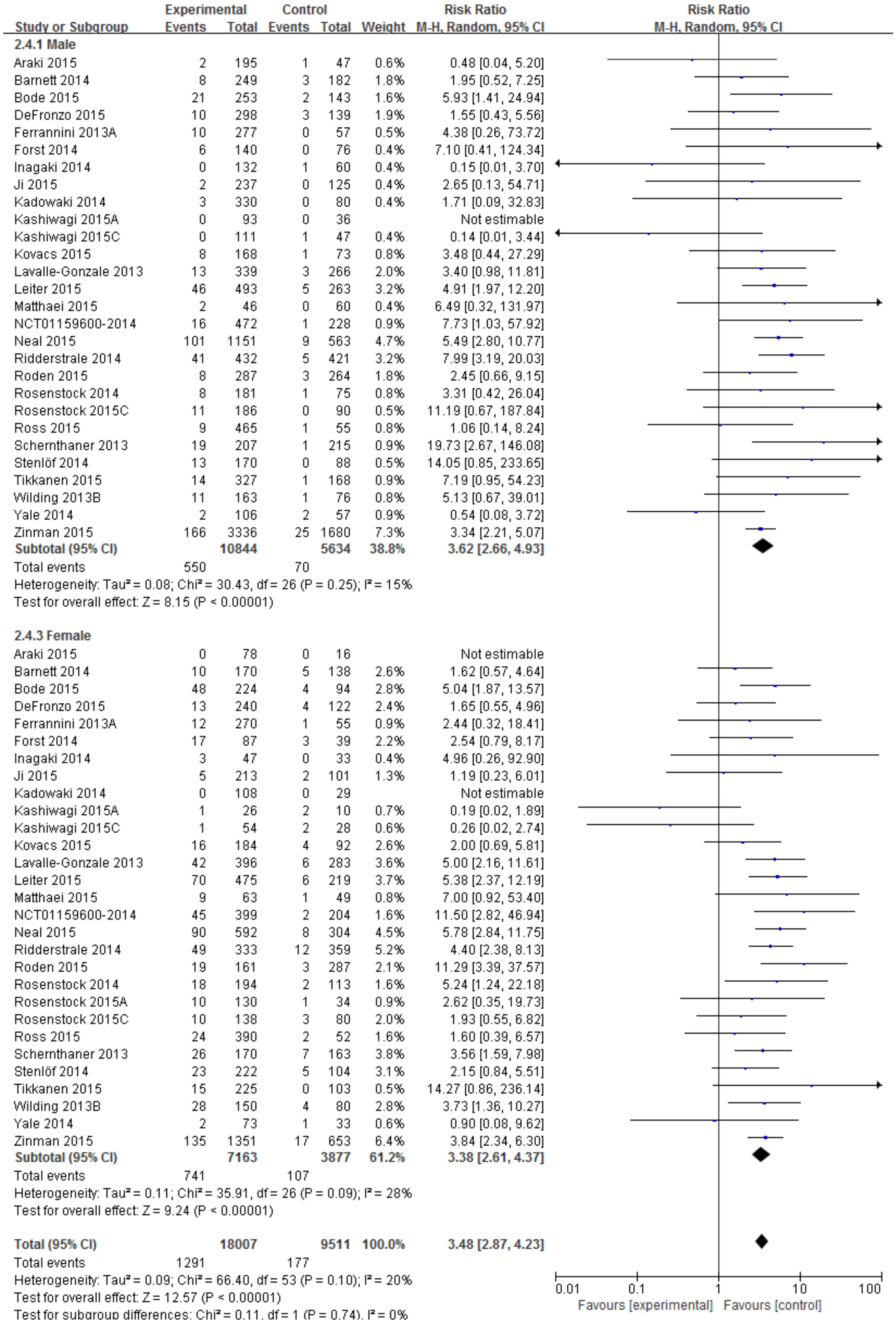

Figure 15S. Subgroup analysis of genital infections by gender in randomized controlled trials

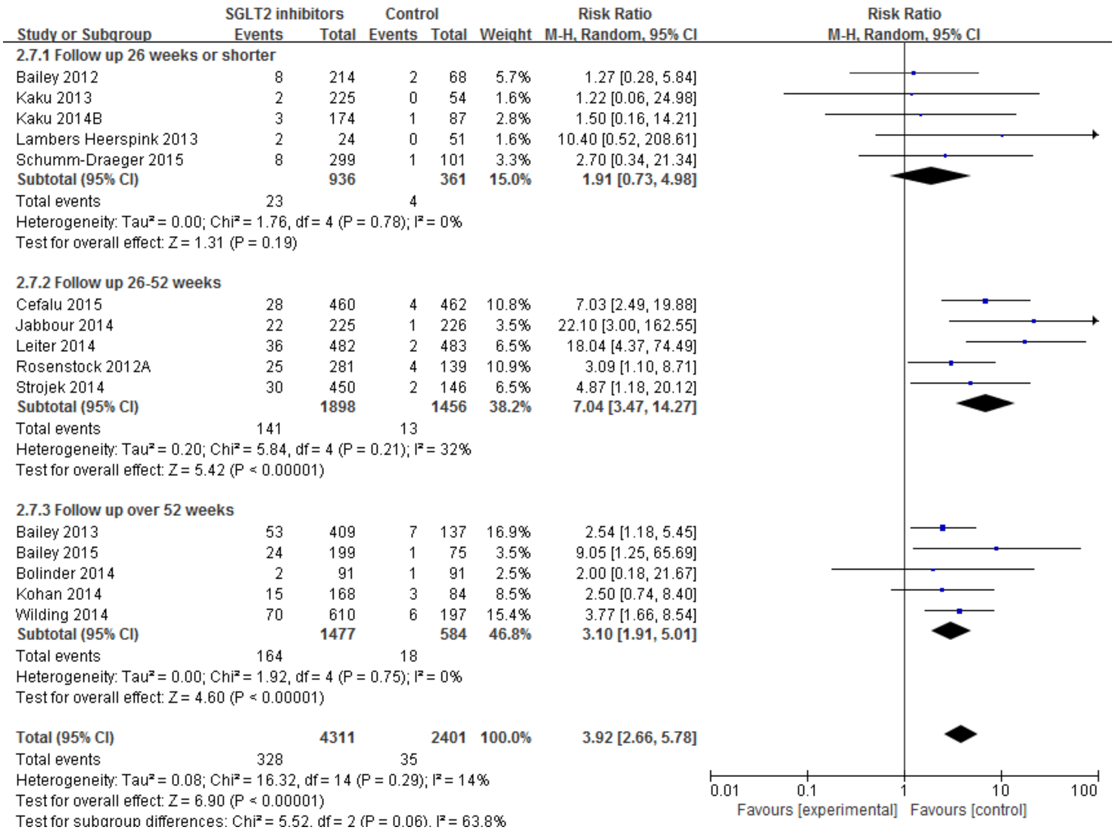

Figure 16S. Subgroup analysis of events suggestive of genital infections by length of follow up in randomized controlled trials

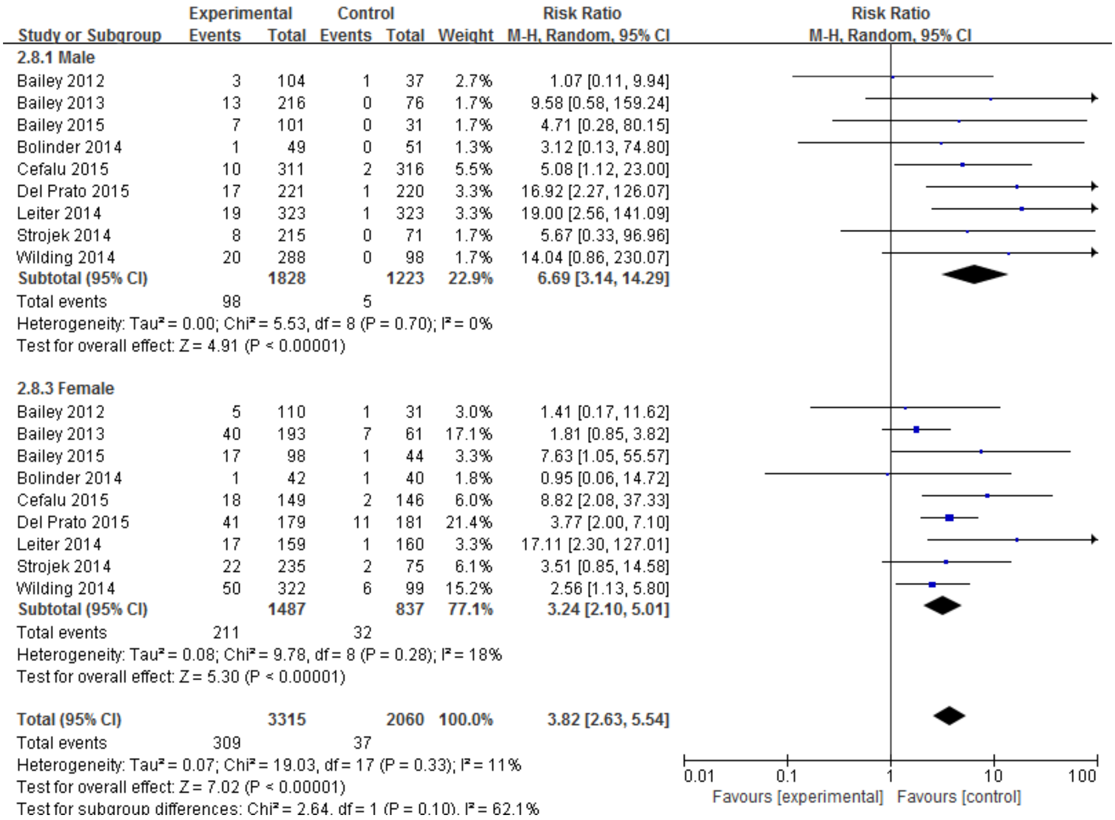

Figure 17S. Subgroup analysis of events suggestive of genital infections by gender in randomized controlled trials

Study or Subgroup	SGLT2 inhibitors Events	SGLT2 inhibitors Total	Control Events	Control Total	Weight	Risk Ratio M-H, Random, 95% CI
**2.9.1 SGLT-2 vs. placebo**						
Bailey 2012	8	214	2	68	5.3%	1.27 [0.28, 5.84]
Bailey 2013	53	409	7	137	18.2%	2.54 [1.18, 5.45]
Bailey 2015	24	199	1	75	3.2%	9.05 [1.25, 65.69]
Bolinder 2014	2	91	1	91	2.2%	2.00 [0.18, 21.67]
Cefalu 2015	28	460	4	462	10.7%	7.03 [2.49, 19.88]
Jabbour 2014	22	225	1	226	3.2%	22.10 [3.00, 162.55]
Kaku 2013	2	225	0	54	1.4%	1.22 [0.06, 24.98]
Kaku 2014B	3	174	1	87	2.5%	1.50 [0.16, 14.21]
Kohan 2014	15	168	3	84	8.1%	2.50 [0.74, 8.40]
Lambers Heerspink 2013	2	24	0	25	1.4%	5.20 [0.26, 103.03]
Leiter 2014	36	482	2	483	6.1%	18.04 [4.37, 74.49]
Rosenstock 2012A	25	281	4	139	10.8%	3.09 [1.10, 8.71]
Schumm-Draeger 2015	8	299	1	101	3.0%	2.70 [0.34, 21.34]
Strojek 2014	30	450	2	146	6.1%	4.87 [1.18, 20.12]
Wilding 2014	70	610	6	197	16.3%	3.77 [1.66, 8.54]
**Subtotal (95% CI)**		**4311**		**2375**	**98.6%**	**3.87 [2.64, 5.66]**
Total events	328		35			
Heterogeneity: Tau² = 0.07; Chi² = 15.96, df = 14 (P = 0.32); I² = 12%						
Test for overall effect: Z = 6.95 (P < 0.00001)						
**2.9.2 SGLT-2 vs. active drugs**						
Lambers Heerspink 2013	2	24	0	26	1.4%	5.40 [0.27, 107.09]
**Subtotal (95% CI)**		**24**		**26**	**1.4%**	**5.40 [0.27, 107.09]**
Total events	2		0			
Heterogeneity: Not applicable						
Test for overall effect: Z = 1.11 (P = 0.27)						
**Total (95% CI)**		**4335**		**2401**	**100.0%**	**3.86 [2.69, 5.54]**
Total events	330		35			
Heterogeneity: Tau² = 0.03; Chi² = 15.99, df = 15 (P = 0.38); I² = 6%						
Test for overall effect: Z = 7.34 (P < 0.00001)						
Test for subgroup differences: Chi² = 0.05, df = 1 (P = 0.83), I² = 0%						

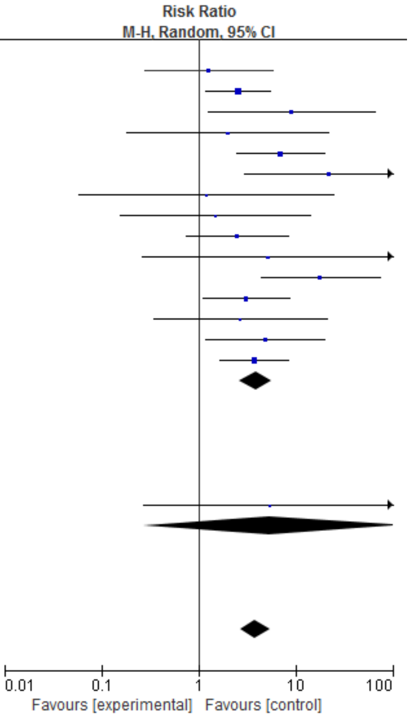

Figure 18S. Subgroup analysis of events suggestive of genital infections by type of control in randomized controlled trials

Supplementary material: Search strategies

PubMed

#1 Diabetes Mellitus, Type 2 [mh]
#2 Type 2 Diabetes Mellitus [tw] OR niddm* [tw] OR mody* [tw] OR DMT2 [tw] OR T2DM [tw] OR stable diabet* [tw]
#3 #1 OR #2
#4 sglt [tw]
#5 sglt2 [tw]
#6 Sodium-Glucose Transport Proteins [mh]
#7 “Sodium-Glucose Transport Protein*” OR "Sodium-glucose transporter" OR “Sodium-glucose co-transporter” OR “Sodium glucose cotransporter”
#8 canagliflozin
#9 canagliflozin[nm]
#10 TA-7284[tw]
#11 TA7284[tw]
#12 JNJ-28431754[tw]
#13 JNJ28431754[tw]
#14 dapagliflozin
#15 dapagliflozin [nm]
#16 BMS-512148[tw]
#17 BMS512148[tw]
#18 empagliflozin
#19 empagliflozin[nm]
#20 BI-10773[tw]
#21 BI10773[tw]
#22 ertugliflozin
#23 ertugliflozin[nm]
#24 PF-04971729[tw]
#25 PF04971729[tw]
#26 henagliflozin
#27 henagliflozin[nm]
#28 SHR-3824[tw]
#29 SHR3824[tw]
#30 ipragliflozin
#31 ipragliflozin[nm]
#32 ASP-1941[tw]
#33 ASP1941[tw]
#34 luseogliflozin
#35 luseogliflozin[nm]
#36 TS-071[tw]
#37 TS071[tw]
#38 remogliflozin
#39 remogliflozin[nm]
#40 sergliflozin
#41 sergliflozin[nm]
#42 sotagliflozin
#43 sotagliflozin[nm]
#44 LX-4211[tw]
#45 LX4211[tw]
#46 tofogliflozin

#47 tofogliflozin[nm]
#48 CSG-452[tw]
#49 CSG452[tw]
#50 RG-7201[tw]
#51 RG7201[tw]
#52 EGT-1442[tw]
#53 EGT1442[tw]
#54 Phlorizin
#55 Phlorizin[nm]
#56 OR/#4-55
#57 randomized controlled trial[pt] OR controlled clinical trial[pt] OR clinical trials as topic [mesh: noexp] OR randomized[tiab] OR randomised [tiab] OR placebo [tiab] OR randomly [tiab] OR trial [ti] OR drug therapy [sh] OR groups [tiab]
#58 “case-control studies”[mh]
#59 “retrospective studies”[mh]
#60 “cohort studies”[mh]
#61 “follow-up studies”[mh]
#62 “longitudinal studies”[mh]
#63 “prospective studies”[mh]
#64 cohort*[tiab]
#65 case control*[tiab]
#66 longitudinal[tiab]
#67 follow up[tiab]
#68 prospective*[tiab]
#69 retrospective*[tiab]
#70 nonrandom*[tiab]
#71 comparison group*[tiab]
#72 control group*[tiab]
#73 database*[tiab]
#74 registries[mh]
#75 registr*[tiab]
#76 OR/#57-75
#77 Animals [mh] NOT humans [mh]
#78 #76 NOT #77
#79 #3 AND #56 AND #78

EMBASE (via OVID)
1. exp non insulin dependent diabetes mellitus/
2.(Diabetes Mellitus, Type 2 or Type 2 Diabetes Mellitus or niddm* or mody* or DMT2 or T2DM or stable diabete*).mp.
3. 1 or 2
4. exp sodium glucose cotransporter 2/ or exp sodium glucose cotransporter 2 inhibitor/
5. sglt.mp.
6. sglt-2.mp.
7. sglt2.mp.
8. Sodium-Glucose Transport Protein$.mp.
9. Sodium-glucose transporter$.mp.
10. Sodium-glucose co-transporter$.mp.
11. Sodium glucose cotransporter$.mp.
12. canagliflozin.mp. or exp canagliflozin/
13. TA-7284.mp.
14. TA7284.mp.

15. JNJ-28431754.mp.
16. JNJ28431754.mp.
17. dapagliflozin.mp. or exp dapagliflozin/
18. BMS-512148.mp.
19. BMS512148.mp.
20. empagliflozin.mp. or exp empagliflozin/
21. BI-10773.mp.
22. BI10773.mp.
23. BI-44847.mp.
24. BI44847.mp.
25. ertugliflozin.mp. or exp ertugliflozin/
26. PF-04971729.mp.
27. PF04971729.mp.
28. henagliflozin.mp. or exp henagliflozin/
29. SHR-3824.mp.
30. SHR3824.mp.
31. ipragliflozin.mp. or exp ipragliflozin/
32. ASP-1941.mp.
33. ASP1941.mp.
34. luseogliflozin.mp. or exp luseogliflozin/
35. TS-071.mp.
36. TS071.mp.
37. remogliflozin.mp. or exp remogliflozin etabonate/
38. KGT-1681.mp.
39 KGT1681.mp.
40. sergliflozin.mp. or exp sergliflozin etabonate/
41. KGT-1251.mp.
42. KGT1251.mp.
43. GW-869682.mp.
44. GW869682.mp.
45. sotagliflozin.mp. or exp sotagliflozin/
46. LX-4211.mp.
47. LX4211.mp.
48. tofogliflozin.mp. or exp tofogliflozin/
49. CSG-452.mp.
50. CSG452.mp.
51. RG-7201.mp.
52. RG7201.mp.
53. R-7201.mp.
54. R7201.mp.
55. T-1095.mp.
56. T1095.mp.
57. ISIS-SGLT2Rx.mp.
58. ISISSGLT2Rx.mp.
59. YM-543.mp.
60. YM543.mp.
61. AVE-2268.mp.
62. AVE2268.mp.
63. EGT-1442.mp.
64. EGT1442.mp.
65. EGT0001442.mp.
66. EGT-0001442.mp.

67. phlorizin.mp. or exp phlorizin/
68. 4 or 5 or 6 or 7 or 8 or 9 or 10 or 11 or 12 or 13 or 14 or 15 or 16 or 17 or 18 or 19 or 20 or 21 or 22 or 23 or 24 or 25 or 26 or 27 or 28 or 29 or 30 or 31 or 32 or 33 or 34 or 35 or 36 or 37 or 38 or 39 or 40 or 41 or 42 or 43 or 44 or 45 or 46 or 47 or 48 or 49 or 50 or 51 or 52 or 53 or 54 or 55 or 56 or 57 or 58 or 59 or 60 or 61 or 62 or 63 or 64 or 65 or 66 or 67
69. Clinical trial/ or Randomized controlled trial/ or Randomization/ or Single blind procedure/ or Double blind procedure/ or Crossover procedure/ or Placebo/
70 Randomi?ed controlled trial$.tw.
71 Rct.tw.
72 Random allocation.tw.
73 Randomly allocated.tw.
74 Allocated randomly.tw.
75 (allocated adj2 random).tw.
76 Single blind$.tw.
77 Double blind$.tw.
78 ((treble or triple) adj blind$).tw.
79 Placebo$.tw.
80 Prospective study/
81. 69 or 70 or 71 or 72 or 73 or 74 or 75 or 76 or 77 or 78 or 79 or 80
82 Case control study/ or Family study/ or Longitudinal study/ or Retrospective study/ or Cohort analysis/
83 (Cohort adj (study or studies)).mp.
84 (Case control adj (study or studies)).tw.
85 (follow up adj (study or studies)).tw.
86 (observational adj (study or studies)).tw.
87 (epidemiologic$ adj (study or studies)).tw.
88 (cross sectional adj (study or studies)).tw.
89. 82 or 83 or 84 or 85 or 86 or 87 or 88
90 81 or 89
91 3 and 68 and 90

Cochrane Central Register of Controlled Trials
1.Diabetes Mellitus, Type 2/
2.(niddm* or mody* or DMT2 or T2DM or stable diabet*).mp.
3.1 or 2
4. exp Sodium-Glucose Transporter 2/
5. sglt.mp.
6. sglt2.mp.
7. sglt next 2.mp.
8. exp Sodium-Glucose Transport Proteins/
9. sodium-glucose co-transporter.mp.
10. sodium-glucose cotransporter.mp.
11. (bexagliflozin or canagliflozin or dapagliflozin or empagliflozin or ertugliflozin or henagliflozin or ipragliflozin or luseogliflozin or remogliflozin or sergliflozin or sotagliflozin or tofogliflozin).mp.
12. 4 or 5 or 6 or 7 or 8 or 9 or 10 or 11
13. 3 and 12

ClinicalTrials.gov
SGLT2 OR bexagliflozin OR canagliflozin OR dapagliflozin OR empagliflozin OR ertugliflozin OR henagliflozin OR ipragliflozin OR luseogliflozin OR remogliflozin

OR sergliflozin OR sotagliflozin OR tofogliflozin | Studies With Results
